# Molecular Characterization and Expression Profiling of the Protein Disulfide Isomerase Gene Family in *Brachypodium distachyon* L

**DOI:** 10.1371/journal.pone.0094704

**Published:** 2014-04-18

**Authors:** Chong Zhu, Nana Luo, Miao He, Guanxing Chen, Jiantang Zhu, Guangjun Yin, Xiaohui Li, Yingkao Hu, Jiarui Li, Yueming Yan

**Affiliations:** 1 College of Life Science, Capital Normal University, Beijing, China; 2 Department of Plant Pathology, Kansas State University, Manhattan, Kansas, United States of America; National Taiwan University, Taiwan

## Abstract

Protein disulfide isomerases (PDI) are involved in catalyzing protein disulfide bonding and isomerization in the endoplasmic reticulum and functions as a chaperone to inhibit the aggregation of misfolded proteins. *Brachypodium distachyon* is a widely used model plant for temperate grass species such as wheat and barley. In this work, we report the first molecular characterization, phylogenies, and expression profiles of PDI and PDI-like (PDIL) genes in *B. distachyon* in different tissues under various abiotic stresses. Eleven PDI and PDIL genes in the *B. distachyon* genome by *in silico* identification were evenly distributed across all five chromosomes. The plant PDI family has three conserved motifs that are involved in catalyzing protein disulfide bonding and isomerization, but a different exon/intron structural organization showed a high degree of structural differentiation. Two pairs of genes (*BdPDIL4-1* and *BdPDIL4-2*; *BdPDIL7-1* and *BdPDIL7-2*) contained segmental duplications, indicating each pair originated from one progenitor. Promoter analysis showed that *Brachypodium* PDI family members contained important *cis*-acting regulatory elements involved in seed storage protein synthesis and diverse stress response. All *Brachypodium* PDI genes investigated were ubiquitously expressed in different organs, but differentiation in expression levels among different genes and organs was clear. *BdPDIL1-1* and *BdPDIL5-1* were expressed abundantly in developing grains, suggesting that they have important roles in synthesis and accumulation of seed storage proteins. Diverse treatments (drought, salt, ABA, and H_2_O_2_) induced up- and down-regulated expression of *Brachypodium* PDI genes in seedling leaves. Interestingly, *BdPDIL1-1* displayed significantly up-regulated expression following all abiotic stress treatments, indicating that it could be involved in multiple stress responses. Our results provide new insights into the structural and functional characteristics of the plant PDI gene family.

## Introduction

Three types of aid proteins are found in the endoplasmic reticulum (ER): peptidyl-prolylcis-transisomerase (PPI), protein disulfide isomerase (PDI), and molecular chaperone binding protein (BiP, also called calnexin/calreticulin proteins). These proteins play a dominant role in assisting nascent polypeptides in folding to form specific three-dimensional conformation of functional proteins by rate-limiting isomerization steps [1]–[3]. Various PDIs function in catalyzing, isomerizing, and reducing/oxidizing the formation of both intra-chain and inter-chain disulfide bonds. This non-specific binding of peptides that assists correct folding is viewed as a chaperone activity and includes calnexin/calreticulin proteins and BiP, but understanding the detailed molecular mechanisms require more in-depth research [4], [5]. PDIs are also the beta-subunit of human prolyl 4-hydroxylase and a component of the microsomal triglyceride transfer protein complex in mammalian cells [6], [7]. The PDI gene family encodes several PDI and PDI-like (PDIL) proteins and is a member of the thioredoxin superfamily that includes glutaredoxins, thioredoxins, ferredoxins, and peroxidoxins [8].

Typical well-known PDIs are composed of two approximately 57 kDa subunits of about 510 amino acids in length and are present in most eukaryotic species [9]. Molecular structures show that PDI family proteins have five discrete domains: a, b, b′, a′, and c [10]. The a and a′ domains have high sequence similarity to thioredoxin, and each contains an active -Cys-Gly-His-Cys- motif embraced by the sequence structures of α-helices and β-strands (β-α-β-α-β-α-β-β-α) that are essential for polypeptide redox and isomerization [11]. The b and b′ domains have low similarity, and the b′ domain provides the principal substrate binding site during isomerisation reactions, but the b domain plays only a structural role without catalytic activity [12]. The C-terminal has a high-capacity Ca2^+^-binding site and ends with a rich acidic amino acid KDEL sequence that functions in ER retention based on a signal peptide sequence at the N-terminal for guiding the polypeptide into the ER lumen [13]–[15].

Many PDI family members, such as ERP57, PDIp, P5, ERP72, PDIR, and PDI-D proteins, have been well studied in higher eukaryotes, especially in mammals. These proteins not only act as redox catalysts and isomerases, but also share other functions including peptide binding, cell adhesion, and chaperone activities [16]. Phylogenetic analyses of these sequences with thioredoxin (TRX) domains in higher plants placed the PDIL multi-gene family members into at least eight subfamilies that can be merged into three major categories on the basis of different compositions of proteins with thioredoxin (TRX) domains [9], [17]. The I, II, III, and VII subfamilies in particular were included in clade I, and in addition to group VII, all had two thioredoxin active domains. Clade II, including subfamilies IV and V with VI and VIII as outliers, demonstrated a high degree of differentiation [17].

PDIs are ubiquitously expressed in almost all tissues and catalyze the formation of intra- and inter-chain disulfide bonds [18], [19]. They play important roles in the maturation of secreted plasma membrane and storage proteins [9]. Twelve, 12, and 21 different PDI genes have been annotated in *Oryza sativa*, *Zea mays*, and *Glycine max*, respectively [17]. Investigations of rice mutant esp2 suggested that *OsPDIL1-1* retains proglutelin to prevent heterotypic interactions with prolamine polypeptides within the ER lumen [20], perhaps an important step in starch synthesis [21]. Over-expression of PDIs and BiPs in developing maize endosperm leads to structural changes in storage proteins; therefore, it is possible that PDIs play important roles in the synthesis and association of protein bodies, or in the interaction of β- and γ-zeins with α-zeins [22]. Different expression levels of PDI family proteins in developing cotyledons of soybeans indicate that they may play specific roles at different stages [23], [24]. GmPDIL-1 and GmPDIL-2 serve as molecular chaperones in supporting the correct folding of glycinin or β-conglycinin in the ER of cotyledon cells [24]. Peak expression of *GmPDIL-3a* and *GmPDIL-3b* during seed maturation indicates that they might be involved in folding or accumulation of storage proteins [25]. *AtPDIL2-1* in *Arabidopsis* affects ovule structure and promotes embryo sac development through correct guidance of pollen tube growth [26]. In addition, AtPDI5 (AtPDIL1-1) chaperones and inhibits Cys proteases during programmed cell death (PCD) of the endothelium in developing seeds [27]. A new function of PDI in *Arabidopsis* was found in biogenesis of transitory starch [28]. The PDI and PDIL proteins also play important roles in the chloroplast, and achloroplast-localized disulfide isomerase (cPDI) provides redox potential to regulate activator proteins binding to the 5′UTR of *psb*A mRNA in chloroplast transcripts [29]. In wheat, nine PDI and PDIL genes were cloned, and their transcriptional levels in endosperm cellularization demonstrated that they were associated with storage protein synthesis and deposition, which is highly related to wheat gluten quality [17].

Abiotic stresses, such as response to chemical reagents, hormones, drought, and high salinity significantly affect plant growth and development. Some PDI family members are induced to improve protein folding and transport during stress. In *Arabidopsis*, 6 genes (*AtPDI1-1*, *AtPDI1-2*, *AtPDI2-1*, *AtPDI4-1*, *AtPDI5-1*, and *AtPDI5-2*) were up-regulated in response to well-documented chemical inducers of UPR, viz.: DTT, Tm, and Me [30]. Maize PDI genes are also more highly expressed under dehydration, cold, salt, and abscisic acid (ABA) stresses [31]. In wheat, *TaPDI1*, *TaPDI2*, and *TaPDI3* showed highly up-regulated expression under drought, heat, and cold stresses in roots and other tissues [32].

As a member of the *Pooideae* subfamily and a temperate wild annual grass endemic to the Mediterranean and Middle East, *B. distachyon* has rapidly become a new model plant for temperate grass species such as wheat and barley, and in recent years it has been studied extensively. Although PDI proteins play crucial roles in plant growth and development, little has been reported about this family in *B. distachyon*. Here we report the first molecular characterization, phylogenetic evolution, and expression profiles of PDI genes in *B. distachyon* in different tissues and under various stresses. Our results provide new insights into the molecular structures, phylogenetic evolution, and functions of the PDI gene family in *B. distachyon*.

## Results

### *In silico* identification of PDI and PDIL genes in *B. distachyon*

**Figure 1:**
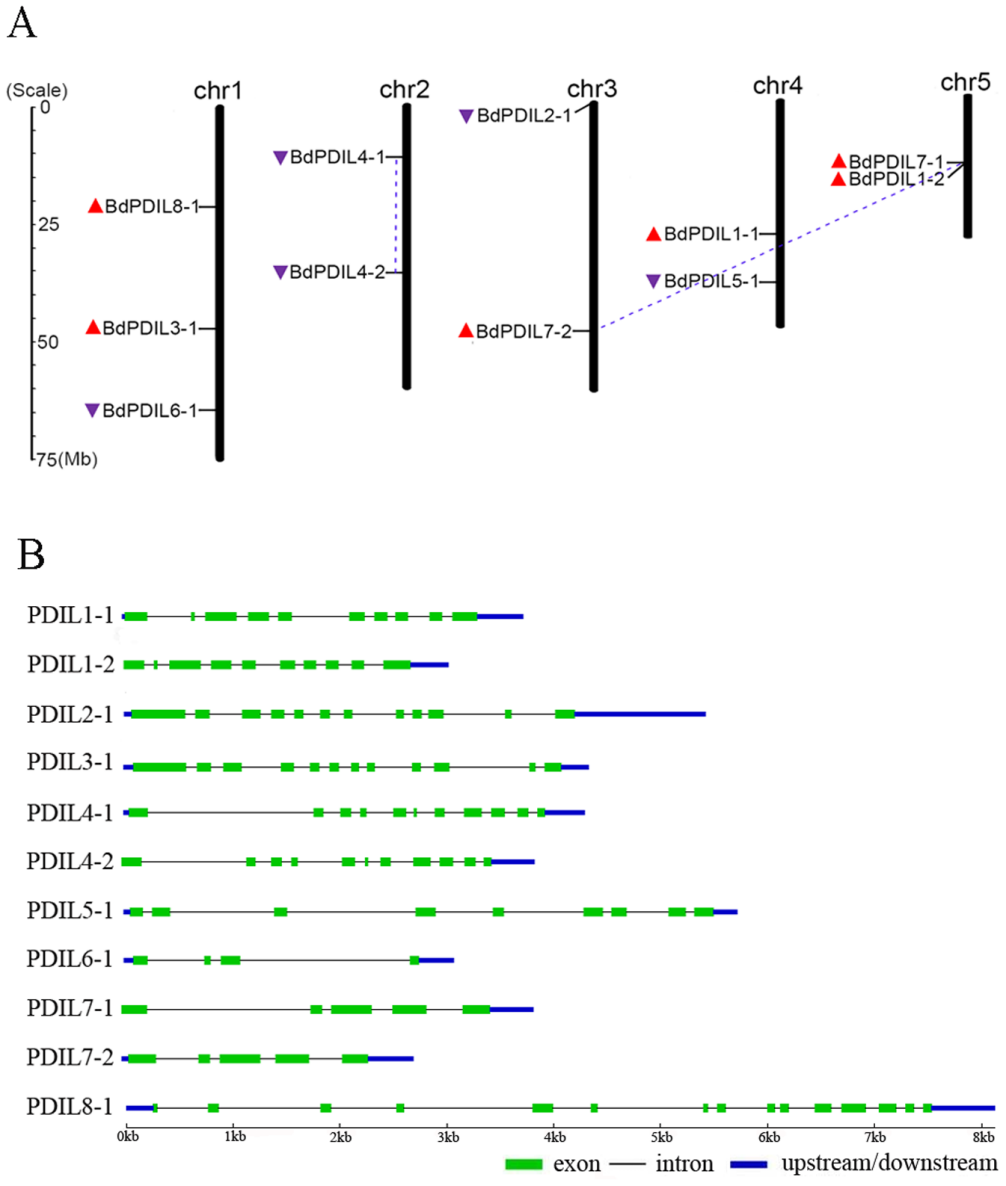
Chromosome distribution and exon-intron structures of 11 *B. distachyon* PDI and PDI-like genes. (A) Chromosome numbers are indicated at the top of each bar; the scales show their size (Mb). Red and purple triangles indicate the upward and downward direction of transcription, respectively. Blue dotted lines connect the PDI genes present duplicate chromosomal segments. (B) Green boxes represent exons. The black solid lines connecting two exons represent introns, and the blue boxes represent upstream/downstream sequences. The grid scales show the gene sizes (kb).

A total of 11 *B. distachyon* PDI genes were confirmed and assigned to 5 different *Brachypodium* chromosomes on the basis of the database search results (Figure 1A). Each chromosome had at least two PDI genes regardless of size. Plant Genome Duplication Database (PGDD) analysis confirmed that the *BdPDIL4-1* and *BdPDIL4-2*, and *BdPDIL7-1* and *BdPDIL7-2* pairs were duplicated sequences. *BdPDIL4-1* and *BdPDIL4-2* were located on the same chromosome but were physically well separated, whereas *BdPDIL7-1* and *BdPDIL7-2* were located on different chromosomes. These genes might have originated from duplication of chromosomal segments. *BdPDIL1-2* was tightly linked with *BdPDIL7-2* on chromosome 5, but both shared higher similarity in amino acid sequences with *BdPDIL1-1* than with each other. Later phylogenetic analysis placed *BdPDIL1-2* and *BdPDIL1-1* in the same subfamily. Interestingly, *BdPDIL1-2* had no duplicated sequences, suggesting that these genes originated from different progenitors. Exon/intron analysis also showed that three pairs of *BdPDIL* genes (*BdPDIL1-1* and *BdPDIL1-2*; *BdPDIL4-1* and *BdPDIL4-2*; and *BdPDIL7-1* and *BdPDIL7-2*) shared similar exon/intron structures (Figure 1B).

The corresponding locus and genetic characteristics of *BdPDI* and *BdPDIL* genes are shown in Table 1. *BdPDIL1-1* had 518 amino acids and molecular mass of 56.79 kDa; *BdPDIL3-1* (543 aa) was the largest PDIL protein, whereas *BdPDIL6-1* (151 aa) was the smallest.

**Table 1:** Characteristics of 11 *Brachypodium distachyon* PDI and PDI-like proteins.

Name	Nomenclature	Genomic (bp)	Length (aa)	CDS	MW	*pI*
Bradi4g23180	BdPDIL1-1	3705	518	1557	56.79	4.86
Bradi5g10610	BdPDIL1-2	3022	520	1563	57.35	4.76
Bradi3g00210	BdPDIL2-1	5420	559	1680	62.08	4.72
Bradi1g48460	BdPDIL3-1	4307	543	1632	59.98	4.96
Bradi2g12560	BdPDIL4-1	4275	367	1104	40.23	6.63
Bradi2g35020	BdPDIL4-2	3804	369	1110	40.07	6.08
Bradi4g31830	BdPDIL5-1	5679	440	1323	47.36	5.34
Bradi1g65710	BdPDIL6-1	3058	151	456	16.97	5.68
Bradi5g10380	BdPDIL7-1	3777	421	1266	46.68	4.77
Bradi3g45540	BdPDIL7-2	2667	423	1272	46.74	4.94
Bradi1g25977	BdPDIL8-1	8119	485	1458	54.37	6.88

### Phylogenetic analysis

**Figure 2:**
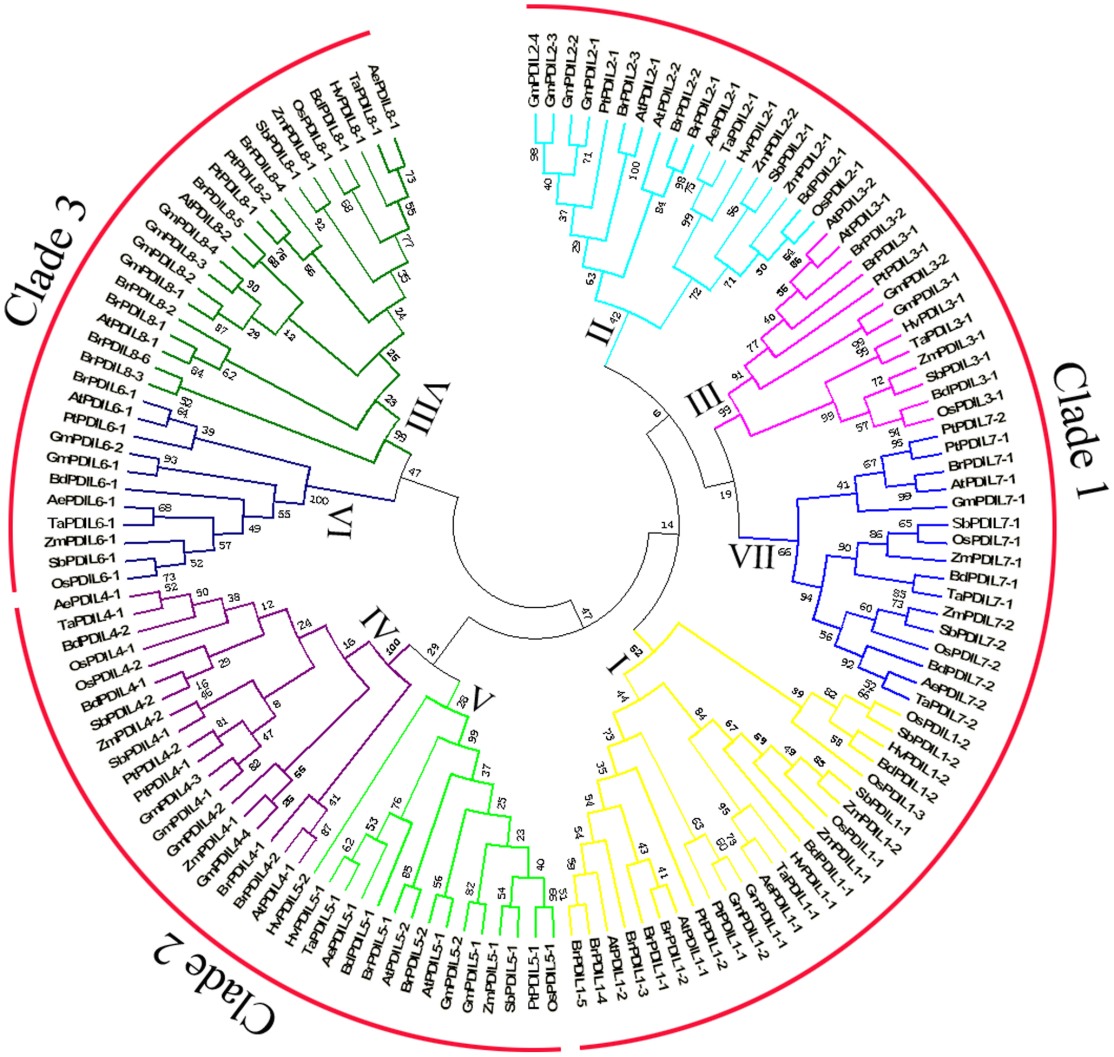
Phylogenetic tree showing relationships between the deduced amino acid sequences of 137 PDI and PDI-like genes from different plant species. 11 from *Brachypodium distachyon* (Bd), 9 from *Triticum aestivum* (Ta), 7 from *Hordeum vulgare* (Hv), 7 from *Aegilops tauschii* (Ae), 12 from *Oryza sativa* (Os), 12 from *Zea mays* (Zm), 21 from *Glycine max* (Gm), 13 from *Arabidopsis thaliana* (At), 11 from *Sorghum bicolor* (Sb), 22 from *Brassica campestris* (Bc),and 12 from *Populus trichocarpa* (Pt). Multiple alignments of sequences were performed by ClusalW, and the phylogenetic tree was constructed by the neighbour-joining (NJ) method and evaluated by bootstrap analysis. Numbers on the main branches indicate bootstrap percentages for 1,000 replicates. The three major clades (1–3) and eight phylogenetic groups (I–VIII) identified in the plant PDI family are highlighted with a red arc and the same color branch, respectively.

To investigate the phylogenic relationships of PDI and PDIL genes between *B. distachyon* and 10 other plant species and to generate an evolutionary framework, an unrooted phylogenetic tree was constructed from alignment of 137 PDI and PDIL amino acid sequences (File S1), including 11 from *B. distachyon* (Bd), 9 from *Triticum aestivum* (Ta), 7 from *Hordeum vulgare* (Hv), 7 from *Aegilops tauschii* (Ae), 12 from *Oryza sativa* (Os), 12 from *Zea mays* (Zm), 21 from *Glycine max* (Gm), 13 from *Arabidopsis thaliana* (At), 11 from *Sorghum bicolor* (Sb), 22 from *Brassica campestris* (Bc), and 12 from *Populus trichocarpa* (Pt). As shown in Figure 2, three major clades and eight phylogenetic groups (I–VIII) were clearly separated among the plant PDI family. The largest, clade 1, included subfamilies I, II, III, and VII and 71 PDI and PDIL genes, whose members contained two active TRX domains at the N- and C-terminal ends. Subfamily I included the typical PDI, which had been published in several plant species. In addition, the VII subfamily proteins possessed only an N-terminal active domain. Evolutionary relationships indicated that these subfamily members may originate from a common progenitor gene, in which the VII subfamily would have emerged by loss of the C-terminal active domain. Clade 2 contained subfamilies IV and V, consisting of 34 PDI and PDIL genes whose proteins possessed two tandem thioredoxin active domains at the N-terminal end. As outgroups, subfamilies VI and VIII with 32 PDI and PDIL genes were included in clade 3; these had a single active thioredoxin domain, suggesting that they underwent greater divergence during evolution.

### BdPDI and BdPDIL protein structure and motif analysis

**Figure 3:**
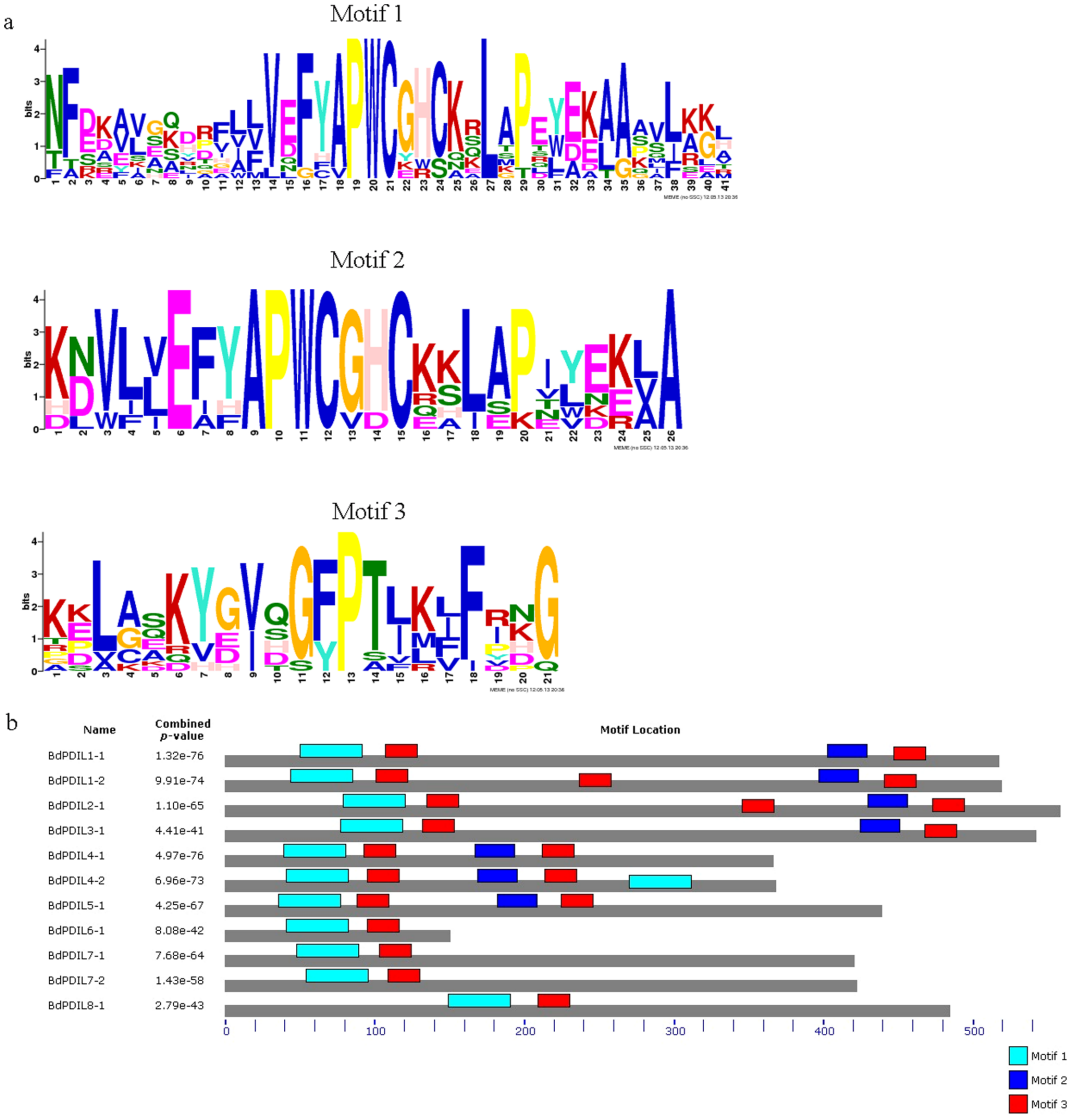
Motif analysis was performed using MEME 4.90 software as described in the methods. (a) Three kinds of motifs were included in the a and a′ domains, which were homologous to thioredoxin (TRX) domains. Motifs 1 and 2 contained single CxxC catalytic motifs. (b) Motif 3 was closely linked with motifs 1 and 2 and contained *cis* pralines (P) near each active site that might be crucial for the catalytic activity of thioredoxin or other functions.

**Figure 4:**
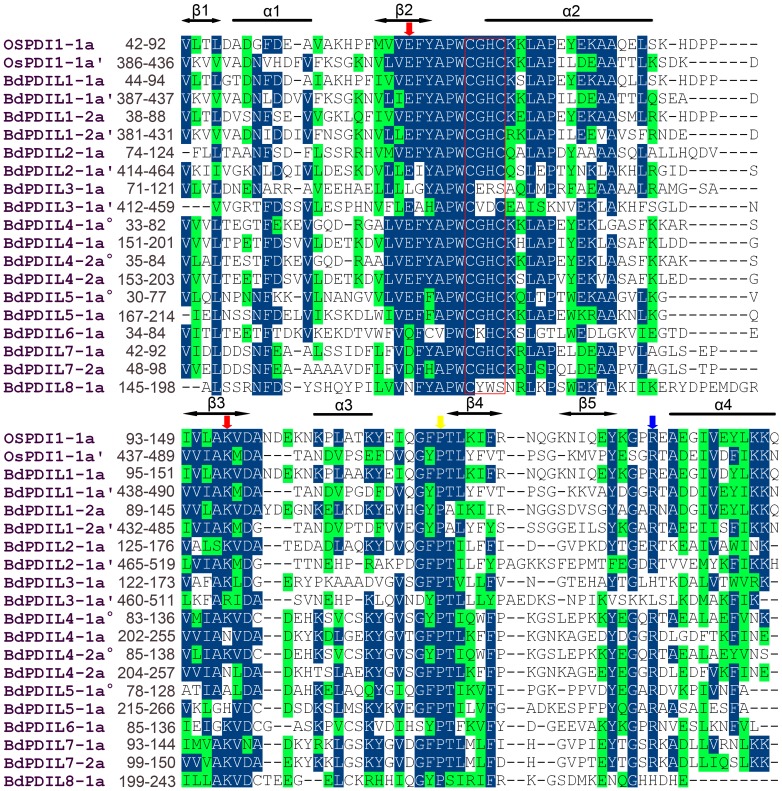
Multiple sequence alignment of *a*-type domains of *B. distachyon* PDI and PDI-like proteins and a typical rice PDI. These thioredoxin-like domains of the *B. distachyon* were annotated in Phytozome database, and comparative analysis used BioEdit software. Residues highlighted in deep blue and green show they were identical and similar, respectively. Open bars and arrowheads represent the α helices and β strands, respectively. The red box indicates the -CxxC- catalytic site, and red arrows indicate the glutamicacid–lysine charged pair. Blue and yellow arrows represent the conserved arginine (R) and the *cis* pralines (P) near the active site, respectively.

Structural characteristics of the 11 BdPDI and BdPDIL proteins (Table 2) were searched using different protein databases; the multiple sequence alignments of thioredoxin-like domains are shown in Figure 3. The conserved motifs of PDI family members were investigated using Meme v4.9 as shown in Figure 4. All BdPDI and BdPDIL proteins had at least a thioredoxin-like domain with the -CXXC- catalytic site necessary for isomerase and redox activities. Motifs 1 and 3 are a fundamental structural combination in all BdPDI family members. In *Arabidopsis* and rice, the homologous domains (motifs1-3) involved in catalyzing protein disulfide bond formation and isomerization are contained in the homologous PDI and PDIL proteins, and their locations are similar to *B. distachyon* (Figure A and B in File S2). In addition, a high-affinity substrate-binding site in the b′ domain modulating the pKa of the two conserved cysteine active sites and a glutamic acid-lysine charged pair involved in proton transfer reactions are prominent determinants of the enzymatic activity of PDI family members [33].

**Table 2:** Structural and functional characteristics of 11 *B. distachyon* PDI and PDI-like proteins.

Name	Signal peptide	Trans-membrane	Domain composition	Active site sequence	Conserved pair charge sequence	Conserved arginine	O-glycosylation sites(putative)	N-glycisilation sites(putative)	C-terminal signal
BdPDIL1-1	1–26	7–24	s-t-a-b-b′-a′	CGHC,CGHC	E65-K99 E409-K442	R139,R478	T254,T511	N286	-KDEL
BdPDIL1-2	1–26	NO	s-a-b-b′-a′	CGHC,CGHC	E59-K93 E403-K436	R133,R473	T507,T509,S515	N45,N305,N344,N363	-KDEL
BdPDIL2-1	1–24	7–24	s-t-c-a-b-b′-a′	CGHC,CGHC	E94-K129 E436-K469	R165,R507	T536	N80,N184,N313	-KDEL
BdPDIL3-1	1–23	7–24	s-t-c-a-b-b′-a′	CERS,CVDC	L92-K126 E431-R464	H162,L501	0	N152	-KDEL
BdPDIL4-1	1–28	11–28	s-t-a°-a-D	CGHC,CGHC	E54-K87 E173-N206	R125,R244	0	0	-TFSS
BdPDIL4-2	1–30	13–30	s-t-a°-a-D	CGHC,CGHC	E56-K89 E175-N208	R127,R246	0	0	-IFSS
BdPDIL5-1	1–22	5–27	s-t-a°-a-b	CGHC,CGHC	E51-A82 E188-H219	R119,R257	0	N164,N170	-NDEL
BdPDIL6-1	1–27	13–32	s-t-a	CKHC	Q56-K89	R126	0	0	-QDEL
BdPDIL7-1	1–24	7–29 384–406	s-t-a-b-b′-t	CGHC	D63-K97	R133	T3	N179	-IHDR
BdPDIL7-2	1–31	12–34 385–407	s-t-a-b-b′-t	CGHC	D69-K103	R139	0	0	-AHQE
BdPDIL8-1	NO	20–42 447–469	t-a-t	CYWS	N164-K208	H240	ND	ND	-GKDI

A, active site containing thioredoxin-like domain; b, inactive thioredoxin-like domain (superscript is included to distinguish between domains of proteins containing more than one a and b domain on the basis of their positions and not on the basis of sequence homology); c, acidic segment; D, Erp29c domain; t, transmembrane domain. The position of conserved charge pair sequence and arginine residues that are considered to be important for the catalytic activity are determined on the basis of multiple alignments of the ***a*** type domains of *Brachypodium distachyon* PDI-like proteins and the classical PDI of *Oryza sativa* [LOC_Os11g09280.1] (Figure 4). ND, not determined because BdPDIL8-1 lacks a putative N-terminal signal peptide. Proteins without signal peptides are unlikely to be exposed to O/N-glycosilation machinery and thus may not be glycosylated *in vivo* even though they contain potential motifs.

**Figure 5:**
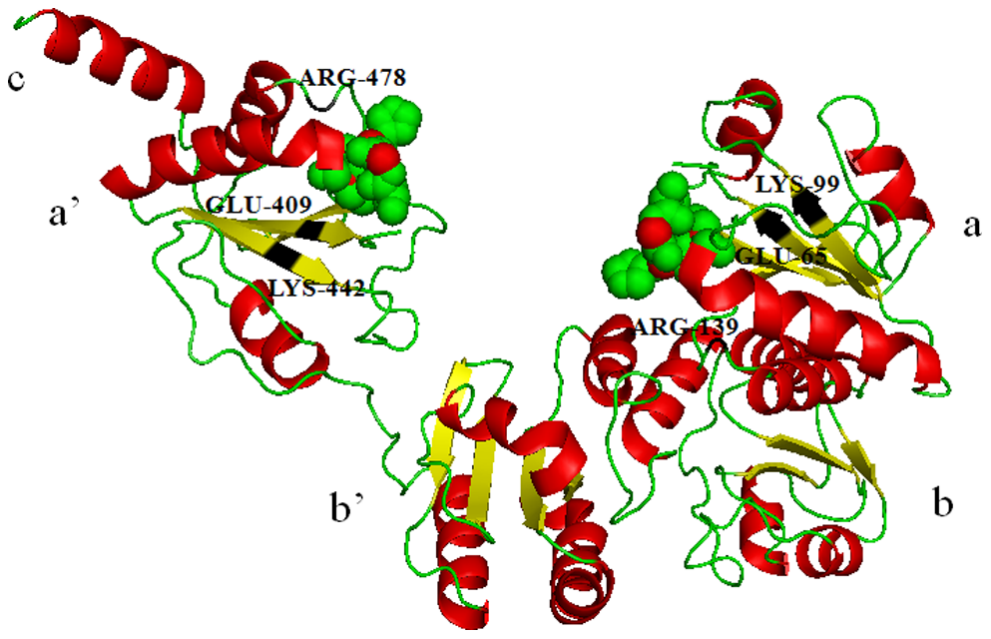
Overall structure of *B. distachyon* PDIL1-1 protein. The tertiary structure was predicted by the Phyre2 server, and the abb′xa′c (five domains and one linker) structure composition indicated similarity with the structure of human and yeast PDI. The -CXXC- catalytic sites are indicated by green and red spheres. The secondary structure is shown with α helixes in red, β sheets in yellow, and loops in green. Some important amino acid (glutamicacid–lysine charged pair and conserved arginine) are marked near each active site on the basis of the characteristics of human PDI.

To better understand structural characteristics, the tertiary structure of a typical *B. distachyon* PDI protein BdPDIL1-1 was predicted by the Phyre2 server and some important amino acids were indicated (Figure 5). The overall structure of BdPDIL1-1 consisted of five domains and one linker (abb′xa′c) in the shape of a twisted “**U**”, similar to the structure of yeast PDI, in which two pairs of active sites facing each other across the long sides and the inside surface of the “**U**” are enriched in hydrophobic residues that would benefit interaction with unfolded or misfolded proteins [34], [35].

Two homologous genes (*BdPDIL1-1*and *BdPDIL1-2*) were included in subfamily I. They encode typical PDI proteins with a multi-domain, including the a-b-b′-a′ thioredoxin-like domain, the 26 aa N-terminal signal peptide, and the C-terminal KDEL signal that guides polypeptide translocation and retention in the ER. In addition, both PDI proteins also contain the -CGHC- motif of the active site (motifs 1 and 2), two charged glutamic acid-lysine pairs (E65-K99 and E409-K442 in BdPDIL1-1; E59-K93 and E403-K436 in BdPDIL1-2), and the conserved arginine residues (R139 and R478 in BdPDIL1-1, R133 and R473 in BdPDIL1-2) involved in modulating the pK_a_ of the cysteine residue active-site as shown in Figure 4.

Subfamilies II and III had only family members BdPDIL2-1 and BdPDIL3-1, respectively. In addition to similar multi-domains, an acidic amino acid–rich sequence named c was present in the N-terminus in both subfamilies. In *Chlamydomonas reinhardtii*, the PDI-like protein (RB60) depended on the N-terminal acidic domain in the chloroplast, which regulated light-induced translation of chloroplast mRNAs through its redox activity [36], [37]. However, BdPDIL3-1 showed some different characteristics: the thioredoxin-box-CGHC- motif was replaced by -CERS- and -CVDC- motifs. Besides, the glutamic acid in the ***a*** domain was changed into a leucine residue and arginine was replaced by histidine, whereas the charged lysine in the ***a′*** thioredoxin domain became arginine and the conserved arginine residues were replaced by leucine. These amino acid changes may result in changes in biochemical properties and functions.

The members of subfamilies IV and V contained BdPDIL4-1, BdPDIL4-2, and BdPDIL5-1, and both had N-terminal signal sequences and two tandem thioredoxin domains (***a°***-***a***). In the a thioredoxin domain of BdPDIL4-1 and BdPDIL4-2, one of the lysines in two charged glutamic acid-lysine pairs was replaced by L-asparagine, and two conserved arginine residues were present (R125 and R244 in BdPDIL4-1; R127 and R246 in BdPDIL4-2). In addition, an about 100 aa ***D*** domain was close to the C-terminus, and the C-terminal retrieval sequence -H/KDEL became -TFSS and -IFSS in BdPDIL4-1 and BdPDIL4-2, respectively. In the ***D*** domain of *D. discoideum* PDI proteins, the 57-residue sequence is necessary and sufficient for ER localization, implying that the ***D*** domain might play a ubiquitous role in retrieval or retention of ER residues to make up for the lack of an H/KDEL-type retrieval sequence [38]. *BdPDIL5-1* had two conserved arginine residues (R119 and R257), an inactive thioredoxin b domain at the C-terminus, and an -NDEL retrieval sequence for its ER localization; however, glutamic acid-lysine charged pairs near the -CGHC- active site in the ***a°*** *-* ***a*** thioredoxin domain was replaced by alanine and histidine, respectively.

In subfamily VII, two homologous PDI members with similar structural features, BdPDIL7-1 and BdPDIL7-2, were included in clade 1, but they retained only single thioredoxin domains and lacked the H/KDEL-type retrieval sequence. The glutamic acid-lysine charged pair was replaced by D-aspartic acid (D63-K97 and D69-K103), and the conserved arginine (R133 and R139) was retained near the -CGHC- active site. Although they lack the H/KDEL-type retrieval sequence, the PDI proteins could be retained in the ER depending on the C-terminal transmembrane segment.

BdPDIL6-1 and BdPDIL8-1 were classified into clade 3 as the outlying subfamily. As the smallest subfamily member in *B. distachyon*, BdPDIL6-1 had a peptide sequence and transmembrane segment in the N-terminus, a single thioredoxin domain, and a -QDEL retrieval sequence for retention in the ER. In addition, the charged glutamic acids were replaced by glutamine. BdPDIL8-1 showed a different structure than other subfamily members, including a non-characteristic tetra-peptide active site -CYWS sequence and two trans-membrane region sequences. An endoplasmic reticulum-Golgi intermediate compartment (ERGIC) domain was located in the N-terminus, and an endoplasmic reticulum vesicle transporter domain (COPII coated_ERV) was located in the C-terminus. These domains are also present in several other proteins, such as ERGIC-32 in mouse and Erv41p and Erv46p in yeast, and play important roles in protein sorting, folding, and glycoprotein processing in the ER and early Golgi complex [39], [40]. The functions of two trans-membrane regions of the BdPDIL8-1 are assumed to replace the function of the N-terminal signal peptide and C-terminal KDEL retrieval sequence, both of which are involved in the early protein secretory pathway between ER, ERGIC, and the Golgi complex.

According to the type of glycosyl chain, two possible types of glycosylation were predicted: O-linked glycosylation and N-linked glycosylation. In yeast and several mammalian species, the PDI might serve as glycoproteins. More credible evidence confirmed that wheat PDIL1-1 is a glycoprotein that retains a glycosylation site in the amino acid sequence [41], [42], suggesting that some BdPDI proteins with putative O/N-glycosylation sites also could be glycosylated.

### BdPDI and BdPDIL gene promoter analysis

Regulation of *PDI* and *PDIL* gene transcription levels in diverse metabolic activities depends on interaction of their *cis*-acting regulatory elements in upstream promoter sequences with different transcription factors [43]. The 2000 bp promoter sequences upstream of the initiation codon of the 11 *BdPDI* and *BdPDIL* genes were searched for *cis*-acting regulatory elements by the Plant CARE database (File S3). These *cis*-acting regulatory elements are mainly related to three important physiological processes: the light cycle, hormonal/environment responses, and seed-specific gene expression. A large number of light-responsive elements were present in the promoter region, suggesting that the *PDI* and *PDIL* genes play important roles in photosynthesis or carbohydrate metabolism. Some other important *cis*-acting regulatory elements are shown in Table 3. Three important seed-specific expression *cis*-motifs (Skn-1_motif, GCN4_motif, and RY-element) were conserved in the promoter regions of some *BdPDI* and *BdPDIL* genes; these are involved in regulating gene expression of cereal grain storage proteins [44], [45]. Transcription levels of PDI genes with these regulatory motifs were higher in immature caryopses than in other tissues. Some stress-related *cis*-acting elements, such as ABRE, MBS, TC-rich repeats, G-Box, and 5′UTRPy-rich stretch, were also present in the PDI promoter region. In *Oryza sativa*, the frequent presence of five *cis*-motifs in the promoter region of oxidative defense pathway genes suggests that *PDI* genes with these motifs are involved in stress defense [46]. All *cis*-acting regulatory elements could individually or interactively regulate expression of *BdPDI* and *BdPDIL* genes in plant growth and development or against various environmental stresses.

**Table 3:** Functional motif numbers of *cis*-regulatory elements identified in *PDI* and *PDI-like* genes of *B. distachyon*.

Motif	Skn-1_motif	GCN4_motif	RY-element	ABRE	MBS	TC-rich repeats	G-Box	5′ UTR Py-rich stretch
function	*cis*-acting regulatory element required for endosperm expression	*cis*-regulatory element involved in endosperm expression	*cis*-acting regulatory element involved in seed-specific regulation	*cis*-acting element involved in abscisic acid responsiveness	MYB binding site involved in drought-inducibility	*cis*-acting element involved in defense and stress responsiveness	*cis*-acting regulatory element involved in light responsiveness	*cis*-acting element conferring high transcription levels
*PDIL1-1*	2			8	1		3	1
*PDIL1-2*	5	2	1		2			1
*PDIL2-1*	4	1		2	3	1	1	5
*PDIL3-1*	3			3	3	1	2	1
*PDIL4-1*	3	1			2	3		1
*PDIL4-2*	4	1		1	2	1		
*PDIL5-1*	2			3		2	3	
*PDIL6-1*	4	1	1	6			2	
*PDIL7-1*	3	1		1			2	1
*PDIL7-2*	2			3	1		2	
*PDIL8-1*	3			1	1		3	

### Differential expression of *PDI* and *PDIL* genes in different organs of *B. distachyon*

PDIs are types of ubiquitously expressed proteins that occur in a variety of tissues [47]. To understand the organ-specificity of PDI family members in *B. distachyon*, expression levels of the 11 *BdPDI* and *BdPDIL* genes in different organs, including roots, stems, and leaves at the two-leaf and heading stages; paleas; lemmas; seeds; and developing caryopses were investigated by qRT-PCR (Figure a, b, c in File S4). The normal expression level of *BdPDIL1-2* was too low to be detected effectively, but it showed higher expression levels under salt and drought treatments.

**Figure 6:**
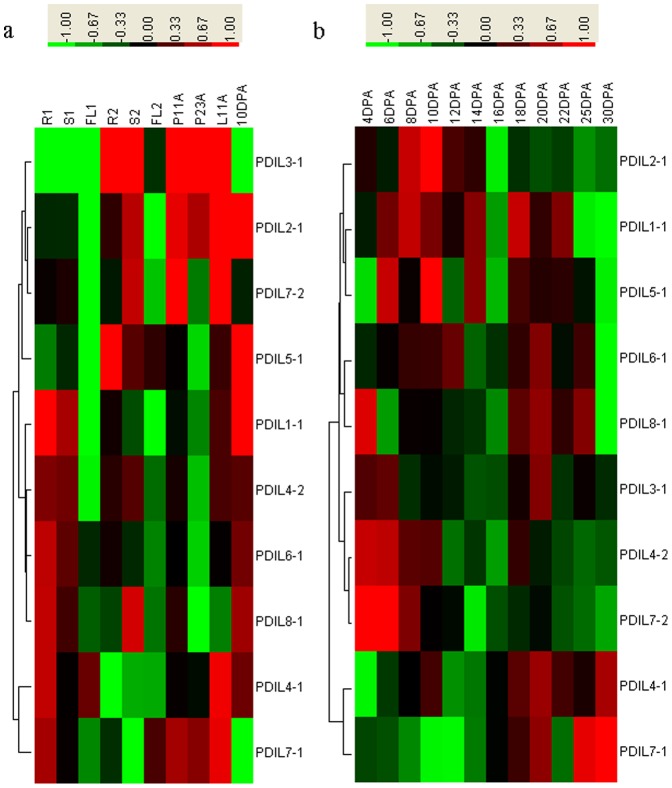
Expression profiling of PDI and PDIL genes in different *Brachypodium distachyon* organs. (**a**) Comparative expression levels of 10 PDI and PDI-like genes in different *Brachypodium distachyon* organs, including roots (R1), stems (S1), and flag leaves (FL1) at the two-leaf stage, stem (S2) and flag leaf (FL2) at heading with 1 cm section of panicle from the leaf sheath; seed palea at 11 and 23 days after anthesis (P11A and P23A); seed lemma at 11 days after anthesis (L11A), and caryopses at 10 days post-anthesis (10 DPA). (**b**) Dynamic expression profiles of 10 PDI and PDI-like genes during seed development in Bd21. Relative quantification of the expression levels in developing caryopses was collected between 4 and 30 DPA. Expression data were obtained from three biological replicates. The relative expression levels at 10 DAP were set to value 1 as the calibrator in the pictures a and b.

As shown in Figure 6 and Figure a, b, c in File S4, all *BdPDI* and *BdPDIL* genes were detected in six different tissues, but with significantly different expression levels at different developmental stages and tissues. *BdPDIL2-1*, *BdPDIL3-1*, and *BdPDIL7-2* showed higher expression levels in roots, stems, paleas, and lemmas at the heading stage, whereas *BdPDIL4-2* was abundant in roots, stems at the two-leaf stage, and early stages of seed development. *BdPDIL1-1*and *BdPDIL5-1* showed higher expression levels in caryopses compared with other organs. *BdPDIL6-1* and *BdPDIL8-1* displayed continuously high expression patterns in caryopses, whereas *BdPDIL4-1* and *BdPDIL7-1* exhibited similar expression profiles in all tissues.

Dynamic transcriptional expression levels of 10 *BdPDI* and *BdPDIL* genes from the *B. distachyon* PDI family at 12 different grain developmental stages were investigated by qRT-PCR, and their expression profiles are shown as heatmaps in Figure 6b. The sampled caryopses covered stages of endosperm development from cellularisation to desiccation. Ten *BdPDI* and *BdPDIL* genes displayed three main expression patterns. The first involved five PDIL genes (*PDIL1-1*, *PDIL2-1*, *PDIL5-1*, *PDIL6-1*, and *PDIL8-1*) and generally showed up-to-down expression trends, although slight expression differences were present; for example, expression of typical PDI gene *PDIL1-1* increased gradually from 4 DPA to its highest value at 8 DPA, then gradually decreased until 16 DPA, after which its expression level increased dramatically again and reached its second highest peak at 18 DPA. Along with seed dehydration, its expression level gradually decreased and reached its lowest level at 30 DPA. Three *BdPDIL* genes (*BdPDIL3-1*, *BdPDIL4-2*, and *BdPDIL7-2*) displayed a different expression pattern with much higher expression levels during early grain development stages (4–6 DPA), then decreased slightly or remained at relatively low levels until 14 DPA. In the following grain development stages, the genes exhibited up-down expression patterns from 16 to 30 DPA, with the highest level at 20 DPA. The remaining *BdPDIL4-1* and *BdPDIL7-1* showed a third expression pattern: gradual up-regulation with the highest level at 30 DPA.

### Expression profiles of PDI and PDIL genes under abiotic stresses

Under natural conditions, a variety of adverse environments, such as drought, salinity, high temperature, and chilling, affect plant growth and development. Artificial stress environments help explore how plants adapt to environmental stress by adjusting morphological, physiological, and molecular mechanisms. The transcriptional expression profiles were obtained for the 11 *BdPDI* and *BdPDL* genes in seedling leaves subjected to various abiotic stress environments, including treatment with 200 mM PEG6000, 160 mM salinity, 0.1 mM ABA, and 20 mM H_2_O_2_.

**Figure 7:**
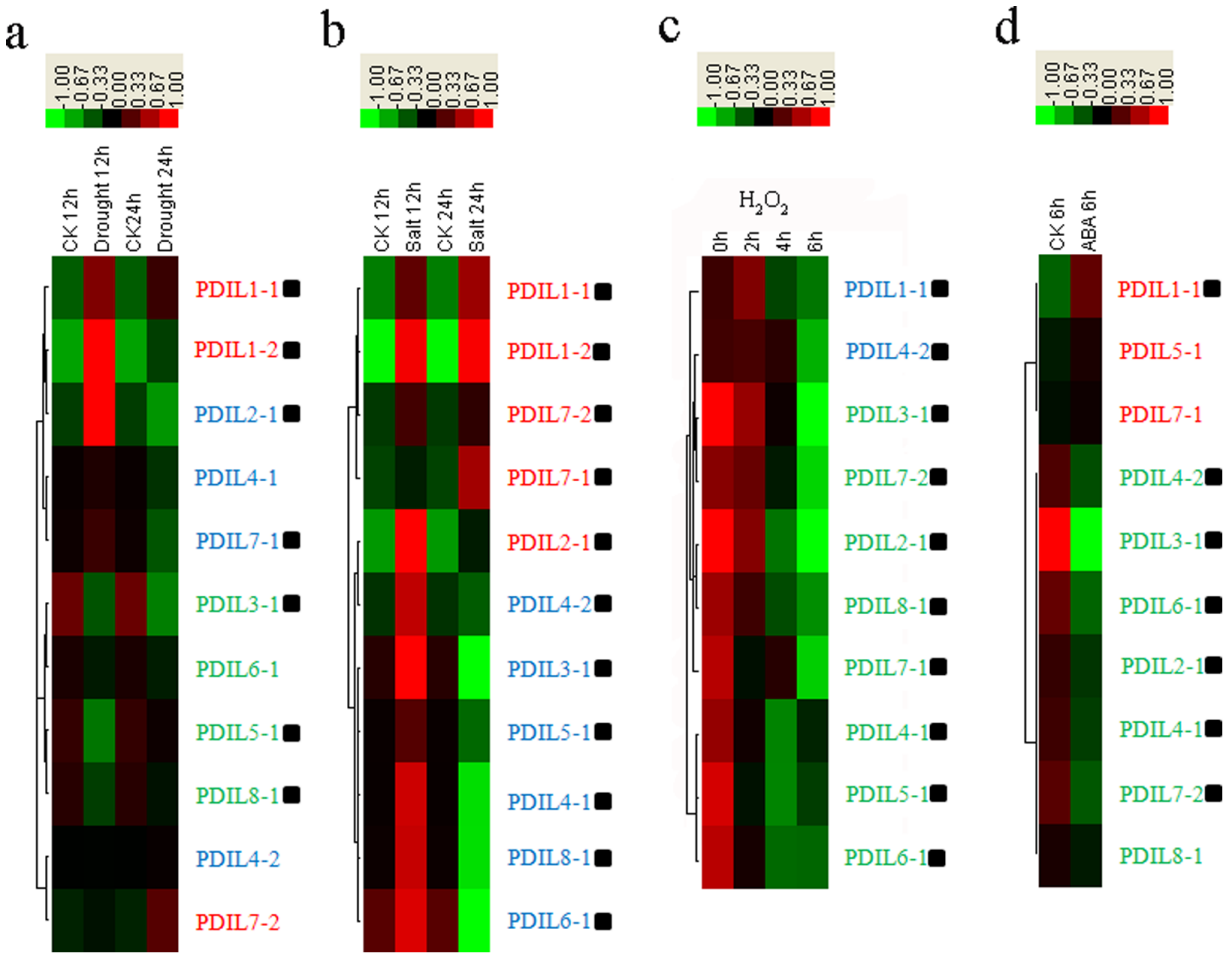
Expression profiles of BdPDI family members in the leaves of *B. distachyon* in response to drought, salt, H_2_O_2_ and ABA, treatments using real-time quantitative RT-PCR and Cluster 3.0 and Java Tree View programs. Blocks with colors indicated decreased (green) or increased (red) transcript accumulation relative to the respective control. The gene expression patterns were labeled in red (up-regulated), green (down-regulated), and blue (up- or down-regulated in different times). Filled squares indicated a significant difference from the control (P<0.05) using SPSS (Statistical Product and Service Solutions) software. Expression profiles of the BdPDI and PDIL genes under: **a**. drought stress for 12 h and 24 h; **b**. salinity stress for 12 h and 24 h; **c**. H_2_O_2_ stress for 0, 2, 4 and 6 h; **d**. ABA stress for 6 h.

Under drought stress induced by PEG 6000, 4 genes (*BdPDIL1-1*, *BdPDIL1-2*, *BdPDIL7-2*, and *BdPDIL2-1*) were significantly up-regulated, whereas 3 genes (*BdPDIL3-1*, *BdPDIL5-1* and *BdPDIL8-1*) were significantly down-regulated (Figure 7a and Figure a in File S5). All *BdPDI* and *BdPDIL* genes were significantly up-regulated at 12 h under salinity treatment. Expression of 6 genes (*BdPDIL3-1*, *BdPDIL4-1*, *BdPDIL4-2*, *BdPDIL5-1*, *BdPDIL6-1*, and *BdPDIL8-1*) began to down-regulate at 24 h under salinity treatment (Figure 7b and Figure b in File S5). Only *BdPDIL1-1* expression was significantly up-regulated at 2 h under H_2_O_2_ treatment; the others were significantly down-regulated until 6 h (Figure 7c and Figure c in File S5). Under ABA treatment, the expression of *BdPDIL1-1* was significantly up-regulated, whereas 6 genes (*BdPDIL2-1*, *BdPDIL3-1*, *BdPDIL4-1*, *BdPDIL4-2*, *BdPDIL6-1*, and *BdPDIL7-2*) showed significantly down-regulated expression (Figure 7d and Figure d in File S5). Interestingly, the typical PDI gene *BdPDIL1-1* was significantly up-regulated under all four stress treatments (Figure 7 a–d and Figure a-d in File S5).

## Discussion

### Molecular characterization and phylogenetic relationships of the PDI and PDIL family of *B. distachyon* compared with each other and with other plant species

Eleven *PDI* and *PDIL* genes were evenly distributed across the five chromosomes of *Brachypodium*; the pairs *BdPDIL4-1* and *BdPDIL4-2* as well as *BdPDIL7-1* and *BdPDIL7-2* shared similar exon/intron structure and motif organizations (Figures 1 and 4). PGDD analysis also confirmed that the two pairs were segmentally duplicated sequence pairs that likely originated from the same progenitor genes; thus, the 11 *BdPDI* and *BdPDIL* genes likely evolved from at least 8 progenitor genes. Protein structure and motif analyses displayed the structure and sequence changes of subfamily members I-VIII. Open reading frames of different size and partial motif deletions and mutations of some important amino acids showed that they had likely undergone a complex evolutionary process involving unequal recombination, duplication, and deletion of gene fragments. These changes influenced their respective functions significantly.

Estimates of divergence times of *Brachypodium* and cultivated crops are 32–39 million years ago (MYA) for wheat, 40–53 MYA for rice, and 45–60 MYA for sorghum [48]; hence, the genetic relationship of *B. distachyon* is much closer to wheat than to rice and sorghum. The phylogenetic tree also showed that *BdPDIL1-1*, *BdPDIL4-2*, *BdPDIL5-1*, *BdPDIL6-1*, and *BdPDIL7-1* genes are much closer to those of wheat than rice or sorghum (Figure 2). PDI and PDIL members from monocots (wheat, rice and maize, barley, sorghum, *B. distachyon*, and *Ae. tauschii*) and dicots (*Arabidopsis*, soybean, poplar, and *Brassica campestris*) generally form two distinct subgroups (Figure 2). Two dicot genes (*PtPDIL4-1* and *PtPDIL4-2*) from *Populus trichocarpa* in subfamily IV, however, were very close to 5 monocots (*ZmPDIL5-1*, *OsPDIL5-1*, *GmPDIL5-1*, *GmPDIL5-2*, and *SbPDIL5-1*) in subfamily V from maize, rice, soybean, and sorghum. This result indicates that a few plant *PDI* genes in subfamilies IV and V are highly conserved in both monocots and dicots; thus, we hypothesize a common evolutionary origin for the plant PDI family in which the PDI genes from monocots and dicots diverged from a single ancestral gene long before divergence through a series of genome segmental duplications and deletions, chromosome fusions and translocations, and ultimately generation of multi-gene PDI families.

### Organ-specificity and dynamic expression profiling of PDI and PDIL genes during grain development and their effects on storage protein synthesis

Transcript expression analysis of PDI family members in six different tissues as revealed by qRT-PCR confirmed that their expression was not organ-specific, which is consistent with most reports [18], [19]. The PDIs play different roles in the maturation of secreted plasma membrane and storage proteins [9]; however, the expression levels of individual PDI family members display greater differentiation. Some members, such as *BdPDIL1-1*and *BdPDIL5-1*, showed higher expression in grains, whereas others, such as *BdPDIL3-1*, showed higher expression levels in other organs (Figure 6), implying that they have specific roles in different organs or different stages of plant development.

The dynamic expression patterns of *BdPDI* and *BdPDIL* genes during seed development are useful for further understanding the molecular mechanisms of the PDI family in protein folding networks, synthesis, and deposition of polymers. As in other cereal crops, grain development from flowering to harvest in *B. distachyon* goes through three stages: embryogenesis, maturation, and dehydration. In this process, PDI and PDI-like proteins play important roles in the correct assembly of both intra-chain and inter-chain disulphide bonds, protein folding, and assembling and accumulating protein bodies. Initial studies in *B. distachyon* showed very low amounts of prolamins in the grains and the main storage proteins were 11S and 7S globulins [49], [50]. Recent studies have found that *Brachypodium* seeds nevertheless contain LMW-GS as well as much lower amounts of HMW-GS [51], [52].

During embryo genesis, seed metabolism is very active as are many metabolic enzymes, such as β-amylase, xyloglucan endotransgly cosidase, and hydrolase [53]. At an early embryogenesis stage (4–6 DPA), four *BdPDIL* genes (*BdPDIL3-1*, *BdPDIL4-2*, *BdPDIL7-2*, and *BdPDIL8-1*) showed high expression levels, with maxima occurring earlier than some amylase genes (Figure 6b), implying that they could be involved in folding and assemby of these functional proteins in preparation for starch synthesis or other metabolic activities. At about 13 DPA, accumulation of storage protein, such as 11S globulin [53], was detected. Expression levels of *BdPDIL1-1*, *BdPDIL2-1*, *BdPDIL4-1*, and *BdPDIL5-1* increased rapidly and reached the first maximum at 8 and 10 DPA, before synthesis of storage proteins, indicating that these PDI proteins play important roles in assisting the formation of intra-chain disulfide bonds in storage proteins. The second peaks of transcription expression occurred at about 20 DPA, when protein synthesis and accumulation increased dramatically and when much larger protein vesicles were distributed in the cytoplasm, suggesting that they were involved in the mass formation of intra-chain and inter-chain disulfide bonds. Promoter analyses also showed that some *cis*-acting regulatory elements required for endosperm expression were present in these genes (Table 3); moreover, four orthologous soybean and wheat PDI genes had similar functions in the folding of seed storage proteins [54], [55].

*BdPDIL4-1* and *BdPDIL7-1* displayed their highest expression levels at the late stage of grain development (about 30 DPA), coinciding with the beginning of seed desiccation and coalescence of large protein vesicles, suggesting that these PDI proteins may be involved in formation or rearrangement of inter-chain disulfide bonds. *TaPDIL7-1* in wheat shared a similar expression pattern and was shown to play an important role in disulphide bond formation at later stages of grain development [55]. Even though *BdPDIL4-1* and *BdPDIL4-2* as well as *BdPDIL7-1* and *BdPDIL7-2* were paired duplication sequences with high similarities in amino acid and exon/intron structural organization, their different expression patterns within pairs indicate that functional differentiation occurred during the evolutionary process.

### Expression features and potential functions of PDI and PDIL genes under different abiotic stresses

**Figure 8:**
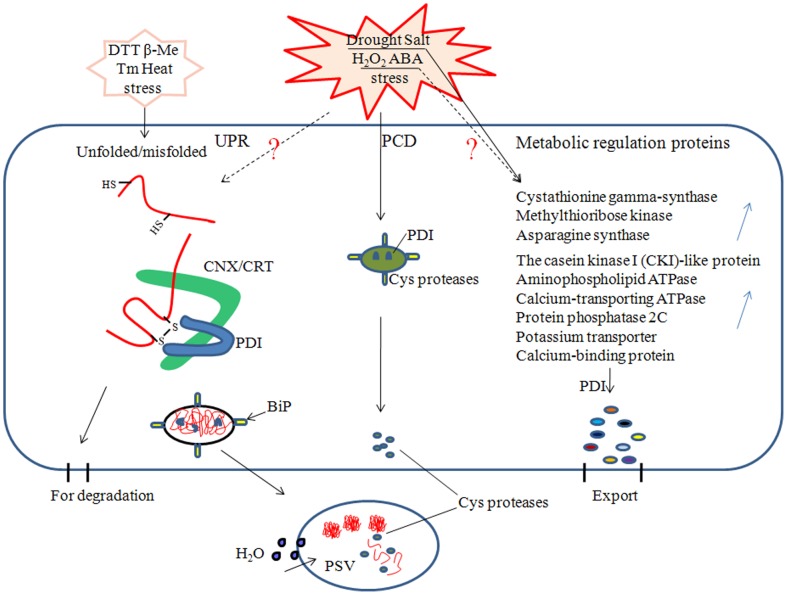
Schematic indicating that the PDI proteins might be involved in three major metabolic pathways (UPR, PCD, and protein folding) in the ER under various adverse stresses. Unfolded proteins response (UPR) and programmed cell death (PCD) induced by the various stresses (cited from reference 56). The PDI proteins catalyzed the formation and rearrangement of disulfide bonds of unfolded or misfolded proteins, but some misfolded proteins still need to be degraded. In *Arabidopsis*, PDIL1-1 protein as the chaperone inhibits Cys proteases from entering the protein storage vacuoles (cited from reference 27). Dashed arrows indicate incompletely understood relationships that need to be further verified. Upward-pointing blue arrows represent metabolic regulation proteins up-regulated under drought and salt stresses to enhance plant tolerance (cited from references 64–66). CNX/CRT, calnexin/calreticulin proteins; PSV, protein storage vacuoles.

Synthesis, folding, and sorting of proteins in the ER are dependent on a series of regulatory gene networks. Adverse environments easily disturb the complex physiological processes leading to unfolding or misfolding of ER proteins [56]. In *Arabidopsis*, 6 genes were up-regulated when induced by dithiothreitol (DTT), beta-mercaptoethanol (β-Me), and tunicamycin (Tm) [30]. Likewise, BiP, SIL1 homolog, peptidyl-prolyl-cis-trans isomerases (cyclophilin), and GRP94 were induced by ER stress in *Arabidopsis* [57]. These folding-related proteins could play different roles in ensuring the correct folding and assembly of nascent polypeptide chains in the ER (Figure 8).

Up-regulated PDI genes such as *BdPDIL1-1* and *BdPDIL2-1* in *B. distachyon* under drought and salt stress contain two active sites, and a C-terminal ER-retention signal could be involved in enhancing protein-folding capacity in the ER. Different *BdPDI* and *BdPDIL* genes that displayed apparently different expression patterns under four different stress treatments imply that their functional differentiation might have occurred during a long-term evolutionary process.

Environmental stresses such as salt, drought, abscisic acid, and H_2_O_2_ also lead to the accumulation of reactive oxygen species (ROS) in plant cells [58]–[60]. PDI has an important function in the thioredoxin-based redox pathway and forms part of the antioxidative defense system [61]. Promoter analysis showed that five *cis*-motifs that occur at high frequencies in the promoter regions of oxidative defense pathway genes are also present in the *BdPDIL1-1*, *BdPDIL2-1*, and *BdPDIL3-1* gene promoters. Up-regulated expression of these PDI and PDIL genes could be involved in stress defense mechanisms. The different numbers of five *cis*-motifs in the promoter and structure characteristics may lead to the different expression levels of *BdPDIL1-1*, *BdPDIL2-1*, and *BdPDIL3-1* under environmental stresses.

Up-regulated expression of *BdPDIL1-1* under H_2_O_2_ treatment suggests that it could be involved in antioxidative defense. A signal pathway that results in programmed cell death (PCD) is initiated and propagated via increasing accumulation of ROS [62], [63]. ROS-induced PCD is regulated by cysteine proteases. In *Arabidopsis*, AtPDIL1-1 protein interacts with three different Cys proteases *in vitro*, suggesting that AtPDIL1-1 as the chaperone inhibits Cys proteases from leaving the ER through the Golgi to vacuoles before the proteases are activated [27], thus delaying the protease entry into the protein storage vacuoles (PSVs), where it degrades the storage proteins (Figure 8).

In wheat, many proteins involved in different metabolic regulation events respond to drought stress, including photosynthesis and the Calvin cycle, cell redox homeostasis, glycolysis and gluconeogenesis, transport and protein-folding mechanisms, protein degradation, and amino acid metabolism [64]. In *B. distachyon*, some proteins were also involved in drought and salt stress responsive mechanisms, such as cystathionine gamma-synthase, methylthioribose kinase, and asparagines synthase, all of which were strongly up-regulated under drought stress (Figure 7a). Casein kinase I (CKI)-like protein, aminophospholipid ATPase, calcium-transporting ATPase, protein phosphatase 2C, potassium transporter, and calcium-binding protein were also up-regulated in response to salt stress [65], [66]. The PDI proteins are necessary to assist folding of nascent polypeptides to form specific functional proteins that regulate metabolic activities and subsequently result in an increase in plant resistance (Figure 8).

*BdPDIL1-1* in *B. distachyon* is homologous to *AtPDIL1-1*, *ZmPDIL1-1*, *OsPDIL1-1*, and *TaPDIL1-1*, which are induced by various stress conditions [21], [30]–[32]. These results provide an important foundation for further functional studies on *BdPDIL1-1*; however, under direct H_2_O_2_ stress treatment, more PDI genes showed down-regulated expression patterns. Low PDI protein expression levels under H_2_O_2_ stress indicated that more reduced enzymes (e.g., reduced glutathione) could effectively be involved in rapid detoxification of O_2_^-^ and H_2_O_2_, leading to reduced hydrogen peroxide damage [58]. Even though these stresses have similar negative impacts on plant growth, the *BdPDI* and *BdPDIL* genes might play different roles in different pathways or in cooperating with other genes to form networks that resist adverse environmental impacts.

## Conclusions

In this study, we identified 11 PDI and PDIL genes in the *B. distachyon* genome by *in silico* analysis. Phylogenetic analysis classified them into three clades and eight subfamilies. Different exon/intron organizations, partial motif deletions, and important amino acid mutations showed that they underwent a high degree of differentiation through unequal homologous recombination, gene duplication, and deletion of gene fragments. The different expression patterns of the *BdPDI* and *BdPDIL* genes in developing caryopses and under various stress conditions further confirmed that their functions were differentiated due to the changed structures. These observations provide a better understanding of the structures and functions of *BdPDI* and *BdPDIL* genes.

## Material and Methods

### Database searches for identifying PDI family members in *B. distachyon* and other plant species

To obtain the *B. distachyon* PDI and PDIL genes, previously cloned sequences of PDI and PDIL genes from rice (12 sequences) and *Arabidopsis* (13 sequences) were used for a BLAST search of two public databases: Species in Phytozomev9.0 (http://www.phytozome.org/) and NCBI (http://www.ncbi.nlm.nih.gov/). In addition, 11 orthologous PDI genes from *Sorghum bicolor*, 22 from *Brassica campestris*, and some of *Hordeum vulgare* and *Aegilops tauschii* PDI genes were also collected from the two public databases. Other plant species PDI genes, such as soybean (*Glycine max*), poplar (*Populus trichocarpa*), and wheat (*Triticum aestivum*) were from published results [17].

### Chromosomal locations, gene duplication, exon/intron organization analyses of PDI genes, and characteristics of PDI proteins in *B. distachyon*

The putative PDI protein sequences were further confirmed and analyzed using the PFAM HMMs [67], CDD (http://www.ncbi.nlm.nih.gov/Structure/cdd/cdd.shtml) from NCBI, InterPro (http://www.ebi.ac.uk/interpro/), and ExPASy (http://www.expasy.org/) databases. The gene locations were based on the Phytozome v9.1 datebase and mapped by Map Inspect software. Identification and cataloging of *B. distachyon* PDI genes in terms of intra-genome or cross-genome syntenic relationships were conducted using the Plant Genome Duplication Database (PGDD) (http://chibba.agtec.uga.edu/duplication/index/locus). Gene transcription directions were searched from Gramene (http://www.gramene.org/Brachypodium_distachyon/Info/Index). The exon/intron organization was analyzed by Gene Structure Display Server (GSDS) (http://gsds.cbi.pku.edu.cn/). The protein pI/Mw were obtained with the Compute pI/Mw tool (http://web.expasy.org/compute_pi/), the presence of signal peptides was confirmed by Signal P4.1 (http://www.cbs.dtu.dk/services/SignalP/), and transmembrane regions were determined by TMHMM ver 2.0 (http://cbs.dtu.dk/services/TMHMM-2.0/) and SMART databases (http://smart.emblheidelberg.de/). N-/O-glycisilation sites and phosphorylation sites were predicted by NetNGlyc 1.0 (http://www.cbs.dtu.dk/services/NetNGlyc/), NetOGlyc 3.1 (http://www.cbs.dtu.dk/services/NetOGlyc/), and NetPhos 2.0 Server (http://www.cbs.dtu.dk/services/NetPhos).

### Phylogenetic analysis

A total of 137 PDI and PDIL sequences from 11 plant species were used to construct a phylogenetic tree. The sequences and respective protein ID or transcript names are displayed in File S1 and S2, and the corresponding nomenclatures were composed of two letters for genus and species, followed by PDIL and an Arabic number. The PDI and PDIL amino acid sequences of the whole coding regions were aligned by Clustal W parameters using the Gonnet series as the protein weight matrix and parameters set to a 5-gap open penalty, 0.55-gap extension penalty, 4-gap separation distance, negative matrix, and end-gap separation on and divergent sequences delay at 20%. The phylogenetic tree was constructed using MEGA software 5.10 [http://www.megasoftware.net/] with the neighbour-joining (NJ) method and 1,000 bootstrap replicates with the bootstrap method.

### Multiple sequence alignment, tertiary structure prediction, conserved motifs, and promoter analysis of *BdPDI* and *BdPDIL* genes

Protein sequence alignment was performed by BioEdit software (http://www.mbio.ncsu.edu/bioedit/bioedit.html). Conserved motifs of PDI family members were investigated using Meme v.4.9 (http://meme.nbcr.net/meme/cgi-bin/meme.cgi). The tertiary structure of *B. distachyon* PDIL1-1 protein was predicted by Phyre2 Server (http://www.sbg.bio.ic.ac.uk/phyre2/html/page.cgi?id=index). The promoter regions were analyzed according to 11 *BdPDI* and *BdPDIL* genes from the *B. distachyon* database with Phytozomev.9.0. Two thousand bp regions upstream of the PDI genes were used in promoter searches for *cis*-acting regulatory elements based on the Plant CARE database (File S3) (http://bioinformatics.psb.ugent.be/webtools/plantcare/html/).

### Plant growth, stress treatments, and sample harvest

*Brachypodium distachyon* 21 (Bd21) seeds were sterilized with 75% alcohol and 15% sodium hypochlorite, rinsed 4–5 times, and placed on moistened filter paper in Petridishes and germinated at 26°C for one week. Ten seedlings were transferred to pots in a growth room at 22°C and a 16 h day/8 h night photoperiod. When the seedlings reached the two-leaf stage, drought, high salinity, ABA, and H_2_O_2_ treatments were initiated. Drought treatment was conducted with 200 mM polyethylene glycol 6000 for 12 and 24 h; salt treatment was 160 mM sodium chloride treatment for 12 and 24 h; 0.1 mM ABA treatment was for 6 h; and 20 mM H_2_O_2_ treatment was for 2, 4, and 6 h. Control and treated leaves were harvested for assays. Developing caryopses were sampled from 4 to 30 days after anthesis (DAA) at 2–5-day intervals (12 sampling times). All samples were immediately frozen in liquid nitrogen and kept at −80°C prior to RNA isolation.

### qRT-PCR

Total RNAs were extracted using TRIzol reagent (Invitrogen) according to the manufacturer's instructions. The Prime Script RT Reagent Kit with gDNA Eraser was used for RNA purification and reverse transcription following the manufacturer's instructions. Transcription levels of PDI family genes in three biological replicates for all treatments were quantified by real-time quantitative reverse transcriptional PCR (qRT-PCR) with a CFX96 Real-Time PCR Detection System (Bio-Rad) using SYBR-green as the intercalating dye and the 2(-Delta Delta C(T)) method [68]. Primer pairs for qRT-PCR analysis (Table a in File S6) were designed by the Primer3Plus program (http://www.bioinformatics.nl/cgi-bin/primer3plus/primer3plus.cgi). PDI primer pairs were checked by blasting primer sequences in the *NCBI* database (http://www.ncbi.nlm.nih.gov/tools/primer-blast/index.cgi?LINK_LOC=BlastHome), and all primers were specifically consistent with the respective sequence of its targeted PDI gene. Real-time melting temperature curves for each *BdPDI* gene showed single peaks and were confirmed by agarose gel electrophoresis (Figure b in File S6). qRT-PCR efficiency was determined by five serial five-fold dilutions of cDNA, and the standard curve confirmed them at high RT-PCR efficiency rates (Figure a, b, c in File S7). *Ubi4* (Bradi3g04730) was used as the reference gene according to previous report [69].

## Supporting Information

File S1 One hundred and thirty-seven PDI and PDI-like amino acid sequences from 11 species used for constructing phylogenetic tree. 11 from *Brachypodium distachyon* (Bd), 9 from *Triticum aestivum* (Ta), 7 from *Hordeum vulgare* (Hv), 7 from *Aegilops tauschii* (Ae), 12 from *Oryza sativa* (Os), 12 from *Zea mays* (Zm), 21 from *Glycine max* (Gm), 13 from *Arabidopsis thaliana* (At), 11 from *Sorghum bicolor* (Sb), 22 from *Brassica campestris* (Bc), and 12 from *Populus trichocarpa* (Pt).
(DOCX)

File S2 Motif, exon/intron structure organizations, and locations in respective sequences of PDI and PDIL family members in *Arabidopsis thaliana* (A) and *Oryza sativa* (B).
(TIF)

File S3 Functional motif numbers of identified *cis*-regulatory elements in PDI and PDIL genes of *Brachypodium distachyon*.
(XLSX)

File S4 Organ-specific expression of *Brachypodium* PDI and PDIL genes in roots, stems, leaves, paleas, lemmas and developing caryopses. The relative expression levels at 10 DAP were set to value 1 as the calibrator in the pictures a and b.
(TIF)

File S5 Relative expression levels of BdPDI family members in the leaves of *B. distachyon* in response to drought (a), salt (b), H_2_O_2_ (c) and ABA (d) stress were analyzed using quantitative RT-PCR and compared with well-watered control plants. The right side of illustrations indicated the treatment time (hour) under corresponding abiotic stresses. Error bars represented the standard deviations of three biological replicates.
(TIF)

File S6 Primer sequences used for real-time quantitative RT-PCR (qRT-PCR).
(DOCX)

File S7 qRT-PCR optimization design: double standard curve and dissolution curve of *BdPDI* and *BdPDIL* genes in different developing organs (a) developing caryopses (b) and leaves under drought, salt, H_2_O_2_ and ABA treatments (c). One of the red standard curves represented BdPDI genes and other blue standard curve represented the reference gene. The dissolution curves of different genes were indicated.
(TIF)
