# Supplementary material for: Molecular Characterization and Expression Profiling of the Protein Disulfide Isomerase Gene Family in Brachypodium distachyon L
Source: PLoS One. 2014 Apr 18;9(4):e94704. doi: 10.1371/journal.pone.0094704 (PMC3991636; doi:10.1371/journal.pone.0094704)
Supplement: File S1 — One hundred and thirty-seven PDI and PDI-like amino acid sequences from 11 species used for constructing phylogenetic tree. 11 from Brachypodium distachyon (Bd), 9 from Triticum aestivum (Ta), 7 from Hordeum vulgare (Hv), 7 from Aegilops tauschii (Ae), 12 from Oryza sativa (Os), 12 from Zea mays (Zm), 21 from Glycine max (Gm), 13 from Arabidopsis thaliana (At), 11 from Sorghum bicolor (Sb), 22 from Brassica campestris (Bc), and 12 from Populus trichocarpa (Pt). (DOCX) [file pone.0094704.s001.docx]

**File S1.** A total of 137 PDI and PDIL amino acid sequences from 11 species used for constructing phylogenetic tree, including 9 from *Triticum aestivum* (Ta), 12 from *Oryza sativa* (Os), 7 from *Hordeum vulgare* (Hv), 11 from *Sorghum bicolor* (Sb), 13 from [*Arabidopsis*](app:ds:arabidopsis) [*thaliana*](app:ds:thaliana) (At), 21 from *Glycine max* (Gm), 12 from *Populus trichocarpa* (Pt), 22 from *Brassica campestris* (Br), 11 from *Brachypodium distachyon* (Bd), 12 from *Zea mays* (Zm) and 7 from *Aegilops tauschii* (Ae).

>TaPDIL1-1 gi|67508771|emb|CAI30635.1|

MAISKVWISLLLALAVVLSAPAARAEEAAAAEEAAAAPEAVLTLHADNFDDAIAKHPFILVEFYAPWCGHCKSLAPEYEKAAQLLSKHDPAIVLAKVDANDEKNKPLAGKYEVQGFPTLKIFRNGGKNIQEYKGPREAEGIVEYLKKQVGPASKEIKAPEDATYLEDGKIHIVGVFTEFSGTEFTNFLELAEKLRCDYDFGHTVHANHLPRGDAAVERPLVRLFKPFDELVVDSKDFDVSALEKFIDASSTPKVVTFDKNPDNHPYLLKYFQSNAPKAMLFLNFSTGPFESFKSAYYGAVEEFSGKDVKFLIGDIEASQGAFQYFGLKEDQAPLILIQDSDSKKFLKEQVEAGQIVAWLKDYFDGKLTPFRKSEPIPEANNEPVKVVVADNIHDVVFKSGKNVLIEFYAPWCGHCKKLAPILDEAAATLQSEEDVVIAKIDATANDVPGEFDVQGYPTLYFVTPSGKKVSYEGGRTADEIVDYIKKNKETAGQAAAAATEKAAEPAATEPLKDEL*

>TaPDIL2-1 gi|299469356|emb|CBG76696.1|

MAAMPMPRSLLLILLLATPLLILPLAAAAVPTSNPDIDLEYLIKNAGLDDPTPATTATDPEDDGAPDFPGLDADYDDEVLFGDDDGPEEDSSHPSAADEAHVLLLTAANFTPVLAARRHVMVEFYAPWCGHCRALAPHYAAAASALAEQGVDVALAKVDATEDHDLAQAHGVQGYPTLLFFIDGVPRDYAGERTKDAIVAWTSKKLGPAVQNLTTADEAEKIVTGDDVAVLAYLDHLSGAHSDELAAASRLEDTISFYQTTSPDVAKLFHIDPEAKRPSVVLLKKEEEKLTVFDGEFRASAIAEFVSANKIPLITTLTQETAPAIFDNPIKKQILLFAVAKESPQFLPIIKETAKSFKGKLLFVFVERDNEEVGEPVANYFGIAGQETTVLAYTGNEDAKKFFFSGEISLDTIKEFAQGFLEDKLTPSYKSDPVPESNDEDVKVVVGKSLDQIVLDESKDVLLEVYAPWCGHCQSLEPIYNKLAKYLRGIDSLVIAKMDGTNNEHPRAKPDGFPTILFYPAGKKSFEPITFEGDRTVVEMYKFLKKHAAIPFKLKRPDSSAARTDGADGSGSTTEGEKSSGSNPKDEL*

>TaPDIL3-1 gi|299469358|emb|CBG91897.1|

MRATRLLAAAALLAALLAVSAAAAKLDLDEVDDSEVLEALLAVDEEEEDAAPPGGGGGAEAVRRTQSMVLVLDNDNAARAVRDHPELLLLGYAPWCERSAKLMPRFAEAAAALRAMGSAVAFAKLDGERFPKAASTVGVNGFPSVLLFVNGTEHAYTGLHTKDAIVTWVRKKTGTPVIRIESKDSAEELLKKGQTFALGLFKNYEGTDHEEFMKAATAENEVQFVETNDRNVAKILFPGIASEEQFLGLVKSEPEKFEKFDGAFEEKEILQFVELNKFPLITVFTDLNSAKVYSSPIKLQVFTFAEAYDFEDLESIVQEVARGFKTKIMFIYVDTAEENLAKPFLTLYGLEGDKPTVTAFDTSKGAKYVLEADINAKNLKEFSLSLLDGTLPPYFRSEPVPQEEGLVEKVVGRTFDSSVLQSPHNILLEAHAPWCVDCEAISKNIEKLAKHFSGLDNLKFARIDASVNEHPKLQVNNYPTLLLYPAEDKTNPIKLSKKLSLKDMARFLKEKLQISDVEIKEKLQTPNIETVAAADNVKDEL*

>TaPDIL4-1 gi|299469360|emb|CBG91898.1|

MATPQIYRKTLLPVLLLLAAAALYPAAADGDEVLALTESTFEKEVGQDRGALVEFYAPWCGHCKKLAPEYEKLAASFKKAKSVLIAKVDCDEHKSVCSKYGVSGYPTIQWFPKGSLEPKKYEGQRTAEALTEYVNSEAATNVKIAAVPSSVVVLTEETFDSVVLDETKDVLVEFYAPWCGHCKSLAPIYEKVASVFKQDEGVVIANLDADKYTSLAEEYGVSGFPTLKFFPKGNKAGEEYESGRELDDFVKFINEKSGTSRDSKGQLTSEAGLVASLDALVKEFHSAADDKRREILSKIEEEAAKLSGPAVKHGKIYVNVAKKILQKGSDYTKKETERLHRLLEKPISPSKADEFAIKKNILSAFSS*

>TaPDIL5-1 gi|299469362|emb|CBG91899.1|

MRPAILAAILPLLAAAASPAAALYSAGSPVLQLNPNNFKKVLNANGVVLVEFFAPWCGLCKQLTPIWEKAAGVLKGVATVAALDADAHKELAQQYGIRGFPTIKVFLPGKPPVDYEGARDVKPIVNFALSQVQGLLRDRLDGKTSGGSSGKTSGGSSEKKNEPNESVELNSSNFDELVVRSKDLWIVEFFAPWCGHCKKLAPEWKRAAKNLKGQVKLGHVDCDSDKSLMSKYKVEGFPTILVFGADKESPFPYQGARAASAIEPFALEQLEANAAPPEVSELTSADVMEEKCASAAICFVSFLPDILDSKAEGRNKYLELLLSVAEKFKKSPYSFVWAGAGKQADLEKQVGVGGYGYPAMVALNVKKGAYAPLRSAFELAEITEFVKEAGRGGKGNLPLEGAPTVVESEPWDGKDGEVIE

EDEFSLEELMADSSAPNDEL*

>TaPDIL6-1 gi|299469364|emb|CBG91900.1|

MDPARRSRLPTHLLLVAVTLLAALAARSGAEVITLTEETFSDKIKEKDTVWFVQFCVPWCKHCKSLGTLWEDLGKVIEGTDEIEIGKVDCGASKPVCSKVDIHSYPTFKVFYDGEEVAKYKGPRDVEALKTFVLKEAEKAGEVRLEDEL*

>TaPDIL7-1 gi|299469366|emb|CBG91901.1|

MAPPPPPPPLPLLLLLLPLLLAPFSATAAAEEFPRDGKVIDLDDSNFEAALSSIDFLFVDFYAPWCGHCKRLAPELDEAAPVLAGLSEPIMVAKVNADKYRKLGSKYGVDGFPTLMLFIHGVPIEYTGSRKADLLVRNLKKFVAPDVSTLESDSAIKSFVENAGTSFPMFIGFGVNESLIAEYGGKYKKRAWFAVAKDFSEDWMATYDFNKIPALVAVHPKYNEQSVFYGPFEGRFLEDFVRQSLLPLTVPINTETLKLLDDDDRKVVLAILEDDSDVNSTQLVKILRSAAHANRDLVFGYVGVKQWEEFVETFDVSKSSQLPKLLVWDRNEEYELVEGSEKLEEGDQASQLSQFLEGYRAGRTIKKKVSGPSFMGFMHSLVSMNSLYILMFVVALLGVMIYFTGQDDTQPRRVHDE*

>TaPDIL7-2 gi|299469368|emb|CBG91902.1|

MPAMAVDKQRLLPLFVLALVTPACLASGGEEPARFQIPQDGSVVELDEGNFEAALAAVDFLFVDFHAPWCGHCKRLSPQLDEAAPVLAGLSTPIVVAKVNAEKYKKLGSKYGVDGFPTLMLFDHGVPTEYTGSREAGQLVESLRKLVAPDVSVLKSDAAIKSFLQEAGVGFPLFIGFGVDESSIAEYGARYKKKAWFSTANDFSEDLMAVYDFDKIPALVSLNPKYNEQSVFYGPFEGTFLEDFIRQSLLPITVPINEETVKMLKDDDRKVVLAILQDESDETSMQLIKVLRSAANANHDLVFGYVGVNQWEEFTEPFHDSKSSQLPKLVVWDKDEEYEVVEGLESLEEGDHGSQISRFLEAYRAGRTIKKTLGRRSPTLLGVNASYILLFLVAVLVVLMFYSGQGEEDRQPTRAHQE*

>TaPDIL8-1 gi|299469370|emb|CBG91903.1|

MISSSKLKSVDFYRKIPRDLTEASLSGAGLSIFAALAMVFLFGMELSSYLAVNTTTSVIVDRSSDGEFLRIDFNLSFPALSCEFASVDVSDVLGTNRLNITKTVRKFSIDRNLVPTGSEFHAGPIPTVNKHGDDVEEYHGDGSVALSSRNFDSYSHLYPVLVVNFYAPWCYWSNRLKPSWEKAAQIIRERYDPEMDGRILLGKVDCTEEVELCKRHHIQGYPSIRIFHKGSDMKENQGHHDHDSYYGERDTESLVAAMETYVANIPKEAHVLALEDKSNRTVDPAKRPAPMTGGCRIEGFVRVKKVPGSVVISARSGSHSFDPSQINVSHYVTTFSFGKRLSSKMFNELKRLFPYVGGHHDRLAGQSYIVKHGDVNANVTIEHYLQIVKTELVTLRYAKELKVLEEYEYTAHSSLVHSFYVPVVKFHFEPSPMQVLVTELPKSFSHFITNVCAIIGGVFTVAGILDSILHNTLRLVKKVELGKDI*

>OsPDIL1-1 LOC_Os11g09280.1

MAISKAWISLLLALAVVLSAPAARAEEAAAAEEGGDAAAEAVLTLDADGFDEAVAKHPFMVVEFYAPWCGHCKKLAPEYEKAAQELSKHDPPIVLAKVDANDEKNKPLATKYEIQGFPTLKIFRNQGKNIQEYKGPREAEGIVEYLKKQVGPASKEIKSPEDATNLIDDKKIYIVGIFSELSGTEYTNFIEVAEKLRSDYDFGHTLHANHLPRGDAAVERPLVRLFKPFDELVVDSKDFDVTALEKFIDASSTPKVVTFDKNPDNHPYLLKFFQSSAAKAMLFLNFSTGPFESFKSVYYGAAEEFKDKEIKFLIGDIEASQGAFQYFGLREDQVPLIIIQDGESKKFLKAHVEPDQIVSWLKEYFDGKLSPFRKSEPIPEVNDEPVKVVVADNVHDFVFKSGKNVLVEFYAPWCGHCKKLAPILDEAATTLKSDKDVVIAKMDATANDVPSEFDVQGYPTLYFVTPSGKMVPYESGRTADEIVDFIKKNKETAGQAKEKAESAPAEPLKDEL*

>OsPDIL1-2 LOC_Os04g35600.1

MAVNLVLSFALAILISSSPTAVGVDATEELKEAVLTLDAGNFSEVVAKHPFIVVKFYAPWCGHCKQLAPEYEKAASILRKNELPVVLAKVDAYNERNKELKDKYGVYSYPTIKIMKNGGSDVRGYGGPREADGIVEYLKRQVGPASLKLESAEEAAHSVVDKGVILVGVFPEFAGMEYENFMVVAEKMRADYDFFHTSDASILPRGDQSVKGPIVRLFKPFDELFVDSEDFGKDALEKFIEVSGFPMVVTYDADPTNHKFLERYYSTPSSKAMLFVSFGDDRIESFKSQIHEAARKFSGNNISFLIGDVADADRVFQYFGLRESDVPLLFVIASTGKYLNPTMDPDQIIPWLKQYIVEYGNLTPYVKSEPIPKVNDQPVKVVVADNIDDIVFNSGKNVLLEFYAPWCGHCRKFALILEEIAVSLQDDQDIVIAKMDGTVNDIPTDFTVEGYPTIYFYSSSGNLLSYDGARTAEEIISFINENRGPKAGAAAAVDEKTQIDAVEEEVTSSSEPVKDEL*

>OsPDIL1-3 LOC_Os02g34940.1

MWPRAPATPPPPPWPSKPSAASRSALRRLDLDDGRRQGTEGEENHAPLLCSPAMASSTAFAAAFALLLLASSAAAEGEAVLTLDAGNFTEVVGAHDFIVVEFYAPWCGHCNQLAPEYEAAAAALRSHDPPVVLAKVDASADLNRGLAGEHGVQGYPTIRILRDRGARSHNYAGPRDAAGIVAYLKRQAGPASVEIAASASPPAADSIANDGVVVVGVFPELSGSEFESFMAVAEKMRADYDFRHTTDAGVLPRGDRTVRGPLVRLFKPFDELFVDSQDFDRDALEKFIESSGFPTVVTFDTSPANQKYLLKYFDNAGTKAMLFLSFSDDRAEEFRTQFHEAANQYSANNISFLIGDVTASQGAFQYFGLKESEVPLVFILASKSKYIKPTVEPDQILPYLKEFTEGTLAPHVKSEPIPEVNDQPVKTVVADNLREVVFNSGKNVLLEFYAPWCGHCQKLAPILEEVAVSLKDDEDVVIAKMDGTANDVPSDFAVEGYPSMYFYSSGGNLLPYDGRTAEEIIDFITKNKGSRPGEATTTESVKDEL*

>OsPDIL2-1 LOC_Os02g01010.1

MRSRSLLLVALATLLLHASASASDDDLDYLIDNADDIPANDPDGWLQEGSPDDDDDDDLFHHGQAQDHPIDETHVFLLSAANFSDFLASHRHVMVEFYAPWCAHCQALAPDYAAAAADLSPLAHQVALAKVDATEDTDLAQKYDVQGFPTILFFIDGVPKDYNGARTKEAIVSWVNKKLAPGVQNITTVDEAEKILTGEDKAILAVLDSLSGAHSDEIAAASRLEDAINFYQTSNPDVAKLFHLDPAAKRPSLVLLKKQEEEKLTFYDGPFKASAIADFVSANKLPLVNTLTQETAPSIFDNPIKKQILLFVVANESSKFLPIFKEASKSFKGKLLFVFVERDNEEVGEPVANYFGITGQETTVLAYTGNEDARNFFLDGEISVENIKRFAEDFLEEKLTPFYKSEPVPESNEGDVKIVVGKNLDQIVLDESKDALLEIYAPWCGHCQELEPTYNKLGKHLRGIDSLVIAKMDGTANEHPRAKPDGFPTILFYPAGKKSFEPITFEGDRTVVEMYKFIKKHASIPFKLKRPDSSATKTEKDQSTASTNLRGERSSGTNFKDEL*

>OsPDIL3-1 LOC_Os06g06790.1

MRARRVVAAAAVLLLFAVVAVARLDLDDDGDDSEVLDELLAVDEEEERGELGGGGEAAAAEAVRRAQSMVLVLDNDNARRAVEENAEVLLLGYAPWCERSAQLMPRFAEAAAALRAMGSAVAFAKLDGERYPKAASAVGVKGFPTVLLFVNGTEHQFTGLHTKDAIVTWVRKKTGAPASRIQSKDSAEEFLKKDQTFAVGLFKNFEGAEYEEFVKAATSENEVQFVETNDRNVAKILFPGIASEEQFLGLVKSEPEKFEKFNGAFEEKEIIQFVELNKFPLITVFTDLNSGKVYGSPIKL

QVFTFAEAYDFEDLESMIQEVARGFKTKIMLIYVDTAEEKLAKPFLTLYGLEPEKPTVTAFDTSKGTKYLMEAEINAKNLQDFCLSLLEGTLPPYFRSEPVPEEKGPIEKVVGRTFDSSVLESPQNVFLEVHAPWCVDCEAISKNVEKLAKHFNDLGQTNLKFARIDASVNEHPKLQINNYPTLLLYPAQDKSNPIKLSKKSNLKDMAKFVKEKLQIADVETVAAGDIVKDEL*

>OsPDIL4-1 LOC_Os05g06430.1

MATPQISRKALASLLLLVAAAAAVSTASADDVLALTESTFEKEVGQDRAALVEFYAPWCGHCKKLAPEYEKLGASFKKAKSVLIAKVDCDEHKSVCSKYGVSGYPTIQWFPKGSLEPKKYEGQRTAEALAEYVNSEAATNVKIAAVPSSVVVLTPETFDSVVLDETKDVLVEFYAPWCGHCKHLAPIYEKLASVYKQDEGVVIANLDADKHTALAEKYGVSGFPTLKFFPKGNKAGEDYDGGRELDDFVKFINEKCGTSRDSKGQLTSEAGIVESLAPLVKEFLGAANDKRKEALSKMEEDVAKLTGPAANRYGKIYVNSAKKIMEKGSEYTKKESERLQRMLEKSISPSKADEFVIKKNILSTFSS*

>OsPDIL4-2 LOC_Os01g23740.1

MAIPRISPRKTLPLFAALALALAWAFAAPAFADGDDVVALTESTFEKEVGQDRGALVEFYAPWCGHCKKLAPEYEKLGASFKKAKSVFIAKVDCDEHKSVCSKYGVSGYPTIQWFPKGSLEPKKYEGQRSAEALAEFVNTEGGTNVKLATIPSSVVVLGPDNFDSIVLDENKDILVEFYAPWCGHCKHLAPIYEKLASVYKLDDGVVIANLDADKHKDLAEKYGVSGYPTLKFFPKGNKAGEDYDGGRELDDFVKFINEKCGTSRDTKGQLTSEAGRIASLDALAKEFLGAANDKRKEILSNMEEEVVKLSGSAAKHGKVYIAIAKKILDKGHDYTKKETERLERMLEKSISPSKADEFIIKKNVLSTFSS*

>OsPDIL5-1 LOC_Os09g27830.1

MRPAVAAALLLVAAAVAASPVSALYSAGSPVLQFNPNNFKSKVLNSNGVVLVEFFAPWCGHCQQLTPIWEKAAGVLKGVATVAALDADAHKELAQEYGIRGFPTIKVFVPGKPPVDYQGARDVKPIVEFALSQVKALLRDRLNGKTSAGSGGKKSGGSSEKTEPSASIELNSQNFDKLVTKSKDLWIVEFFAPWCGHCKKLAPEWKKAAKNLKGQVKLGHVDCDAEKSLMSKYKVEGFPTILVFGADKESPFPYQGARVASAIESFALEQLEANAAPPEVSELTGPDAMEEKCASAAICF

VSFLPDILDSKAEGRNKYLELLLSVAEKFKKSPYSFVWTAAGKQADLEKQVGVGGYGYPAMVALNVKKGAYAPLRSAFQLDEITEFVKEAGRGGKGNLPLDGTPTIVQSEPWDGKDGEVIEEDEFSLEELMADNSPVNDEL*

>OsPDIL6-1 LOC_Os03g17860.1

MDLAPGRRARLLVALALVVLVALAARSGAEVITLTEETFSDKIKEKDTVWFVKFCVPWCKHCKNLGTLWEDLGKVMEGADEIEIGQVDCGVSKPVCSKVDIHSYPTFKVFYEGEEVAKYKGPRNVESLKNFVSDEAEKAGEAKLQDS*

>OsPDIL7-1 LOC_Os04g35290.1

MAATTTRPLPLLLLLLLPPLLLLLLSFHAAAAAAAEEFPRDGRVIELDESSFEAALGAIDYLFVDFYAPWCGHCKRLAPELDEAAPVLAGLSEPIIVAKVNADKYRKLGSKYGVDGFPTLMLFIHGVPIEYTGSRKADLLVRNLNKFVAPDVSILESDSAIKSFVENAGTSFPMFIGFGVNESLIAGYGGKYKKRAWFAVAKDFSEDFMVTYDFDKVPALVSLHPKYKEQSVFYGPFEGSFLEDFIRQSLLPLTVPINTETLKMLDDDDRKVVLAILEDDSDETSSQLVKVLRSAANANRDLVFGYVGIKQWDEFVETFDISKSSQLPKLIVWDRNEEYEVVEGSEKLEEGDQASQISQFLEGYRAGRTTKKKVSGPSFMGFLNSLVSLNSLYILICVFALLGVMIYFTGQDDTPQVRRAHEE*

>OsPDIL7-2 LOC_Os02g34530.1

MGKPTLPPVVVVVVLLLLVVVLPATTCGADAGGGGEAEEFQIPRDGRVLELDDGNFDAAVRAAGLLFVDFYAPWCGHCKRLAPQLDEAAPVLAGLSTPIVVAKVNADKYKKLGSKYGVDGFPTLMLFDHGTPTEYTGSRKADLLVENLKKLVAPDVSVLESDSAIKSFVEDAGMGFPLFLGFGVDESLIVEYGAKYKNRAWFSVAKDFSEDMMVFYDFDKVPALVSVNPKYREQSIFYGPFDDGAFLEDFIRNSLLPLVVPMNRETVKMLNDDGRKVVLMILQDDESDENSPRLIKVLRSAASANRDLVFGYVGVNQWEEFTETFDVKSSELPTMIVWDKKEEYEIVEGSERLEEGDYGSQISRFLEGYRAGRTIKKKVGDRSPTLLGVNAVYILVFLVAVLVLLMYFSGQGEEDQRPRQRAHED*

>OsPDIL8-1 LOC_Os07g34030.1

MISSSKLKSVDFYRKIPRDLTEASLSGAGLSIVAALAMVFLFGMELSNYLAVNTSTSVIVDRSSDGEFLRIDFNLSFPALSCEFASVDVSDVLGTNRLNITKTVRKYSIDRNLVPTGSEFHPGPIPTVSKHGDDVEENHDDGSVPLSSRNFDSYSHQYPVLVVNFYAPWCYWSNRLKPSWEKTAKIMRERYDPEMDGRIILAKVDCTEEIDLCRRHHIQGYPSIRIFRKGSDLKENQGHHDHESYYGDRDTESLVAAMETYVANIPKDAHVLALEDKSNKTVDPAKRPAPLTSGCRIEGFVRVKKVPGSVVISARSGSHSFDPSQINVSHYVTQFSFGKRLSAKMFNELKRLTPYVGGHHDRLAGQSYIVKHGDVNANVTIEHYLQIVKTELVTLRSSKELKLVEEYEYTAHSSLVHSFYVPVVKFHFEPSPMQVLVTELPKSFSHFITNVCAIIGGVFTVAGILDSIFHNTLRLVKKVELGKNI*

>HvPDIL1-1 BAJ89205

MAISKVWISLLLALAVVLSAPAARAEEAAAAEEAAAPEAVLTLHADNFDDAIAQHPFILVEFYAPWCGHCKSLAPEYEKAAQLLSKHDPAIVLAKVDANDEKNKPLAGKYEVQGFPTLKIFRNGGKSIQEYKGPREAEGIVEYLKKQVGPASKEIKAPEDATYLEDGKIHIVGVFTEFSGPEFTNFLEVAEKLRSDYDFGHTVHANHLPRGDAAVERPVVRLFKPFDELVVDSKDFDVSALEKFIDASSTPKVVIFDKNPDNHPYLLKFFQSNAPKAMLFLNFSTGPFESFKSAYYGAVEEFSGKDVKFLIGDIESSQGAFQYFGLKVDQAPLILIQDGDSKKFLKEHVEAGQIVAWLKDYFDGKLTPFRKSEPIPEANNEPVKVVVADNVHDVVFKSGKNVLIEFYAPWCGHCKKLAPILDEAAATLQSEEDVVIAKMDATENDVPGEFDVQGYPTLYFVTPSGKKVSYEGGRTADEIVDYIRKNKETAGQAAAATEKAAEPAATEPLKDEL*

>HvPDIL1-2 BAJ99269

MYVPLALPFATLALVLLLSSGSIAAEVDATAMPGEAVLTLDAGNFSEVVTKHEFIVVEFYAPWCGHCKELAPEYEKAASVLRKRDPPVVLAKVDAYDESNKELKDKYKVHGYPAIKIIRKGGSDVSAYGGPRDAEGIVEYLMRQVGPASLEIKSAVDASRSIGDKGVVLVGVFPEFAGIEYENFMAVANKMRTDYDFFHTLDASILPRGDLTVKGPLIRLFKPFDELFVDSQDFDSDAIKKFIEVSGFPTVVTFNADPTNHKFIERYYSTPSAKAMLFLRFNDDRVETFKSQMHEAARQLSGNNISFLIGDVSTADRAFEYFGLKESDVPLLLVLASTGKYLNPTMEPDQLIPWMKQYIYGNLTPYVKSESIPKVNDQPVKVVVADNIDEIVFNSGKNVLLEFYAPWCGHCRKLAPILEEVAVLLQDDKDVVIAKMDGTANDIPTDFSVEGYPALYFYSSSGGNLLLYDGPRKADEIISFIKKNRGAKAAAAEVTQMDDVEEEVTSSTPSESVRDEL*

>HvPDIL2-1 BAK01309

MAAMSMTRSLLLLLLLATPCLLASSTPTSNPDIDLDYLIKNAGLDDTTTEDAAPDFPGLDADYDDDEEDLFDDDDGPEAESSSAASQDQEAVDEAHVLLLTAANFTSVLAARRHVMVEFYAPWCGHCRALAPHYAAAAAHLALDQPGLDVALAKVDATEDHDLAQAHDVQGYPTLLFFIDGVPRDYAGERTKDAIVAWITKKLGPAVQNLTAVDEAEKIVTGDDVAVLAYLHHLSGAHSDELAAASRLEDTVSFYQTTSPDVAKLFHIDPEAKRPSVVLLKKEEEKLTVFDGEFRASAIAEFVSANKIPLITTLTQETAPAIFDNPIKKQILLFAVAKESSKFLPILKETAKSFKGKLLFVFVERDNEEVGEPVADYFGITGQETTVLAYTGNEDAKKFFFSGEISLDSIKAFAQDFLEDKLTPFYKSDPVPESNDEDVKVVVGKSLDQIVLDESKDVLLEIYAPWCGHCQSLEPIYNKLAKFLHGIDSLVIAKMDGTNNEHPRAKPDGFPTILFYPAGKKSFEPITFEGDRTVVEMYKFLKKHAAIPFKLKRPGSYSSATQTDSTDGPGSSTEAEKSSGSNPKDEL*

>HvPDIL3-1 BAJ98166

MRATRRLLAAAALLAALLAVSAAAKLDLDDVDDSEVLEALLAVDDEEEAAPPGSGGGGGGGAEAVRRTQSMVLVLDNDNAARAVQDHPELLLLGYAPWCERSAQLMPRFAEAAAALRAMGSAVSFAKLDGERFPKAAAAVGVNGFPSVLLFVNGTEHPYTGLHTKDAIVTWVRKKTGTPVIRLESRDSAEEFLKKGQTFALGVFKDYEGADHEEFVKAATAENEVQFVETNDRNVAKILFPGIASEEQFLGLVKNEPEMFEKFDGSFEEKEIIQFVELNKFPLITVFTDLNSAKVYSSPIKLQVFTFAEAYDFEDLESIVQEVARGFKTKIMFIYVDTAEENLAKPFLTLYGLEGDKPTVTAFDTSKGTKYLLEADINTKNLKEFCLSLLDGTLPPYFRSEPVPQEKGLVEKVVGRTLDSSVLQSPHNVLLEAYAPWCVDCEAISKNIEKLAKHFSGLDNLKFARIDASVNEHPKLQVNNYPTLLLYPAEDKTNPIKLSKKLSLKDMARFIKEKLQISDVEIKEKLQTPDVETVAAADNVKDEL*

>HvPDIL5-1 BAJ84858

MRPAILAALLLLLAAAASPAAALYSAGSPVLQLNPNNFKKVLNANGVVLVEFFAPWCGHCKQLTPIWEKAAGVLKGVATVAALDADAHKELAQQYGIRGFPTIKVFLPGKPPVDYEGARDVKPIVNFALSQVKGLLRDRLDGKASGGSSSKTSGGSSEKKNEPNESVELNSSNFDELVIKSKDLWIVEFFAPWCGHCKKLAPEWKRAAKNLKGQVKLGHVDCDSDKSLMSKYKVEGFPTILVFGADKDSPFPYQGARAASAIESFALEQLEANAAPPEVSELTSADVMEEKCASAAICFVSFLPDILDSKAEGRNKYLELLLSVAEKFKKSPYSFVWAGAGKQADLEKQVGVGGYGYPAMVALNVKKGAYAPLRSAFELAEITEFVKEAGRGGKGNLPLEGAPTVVQSEPWDGKDGEVIEEDEFSLEELMADSSAPNDEL*

>HvPDIL5-2 BAJ90795

MNPLWCALLVALANSAVAIYPSNSDVIELTDDNFNQVLQSVEIWVVEFYAPWCGHCQRLVPEYTKAAKALKGIVKVAAIDADKYPSFAGRYGVQGFPTVKIFVDKNKPQDFTGDRTAVGITDEVIKAIKNAISANLQGVPYGSSKSSKKSSSGDDVVELTDSNFDKLVLNSDDIWLVEFFAPWCGHCKNLAPHWAAAASELKGKVKLGALDATVHSSKAQEFNIRGYPTIKFFPSGTSSSSGAEEYTGGRTSSDIVSWAMQKHQENVPPPDIIEIVNEDTFKAGCSEHALCVVSVLPHILDCQASCRNEYLNTLRSLGDKFKQKLWGWLWAEAGKQPELESTLEIGGFGYPALAVLNVKKMKYSILRGSFSEDGIKEFLRDLSYGRGTTAPVKGAALPEIQATEPWDGKDGELPTADDIDLSDVDLDELPKEEL*

>HvPDIL8-1 BAJ86285

MISSSKLKSVDFYRKIPRDLTEASLSGAGLSIFAALAMVFLFGMELSSYLAVNTTTSVIVDRSSDGEFLRMDFNLSFPALSCEFASVDVSDVLGTNRLNITKTVRKFSIDRNLVPTGSEFHAGPIPTVNKHGDDVEEYHGDGSVALSSRNFDSYSHQYPVLVVNFYAPWCYWSNRLKPSWEKAAQIIRERYDPEMDGRILLGKVDCTEEVELCKRHHIQGYPSIRIFHKGSDMKENQGHHEHDSYYGERDTESLVAAMETYVANIPKEAHVLALEDKSNKTVDPAKRPAPMTGGCRIEGFVRVKKVPGSVVISARSGSHSFDPSQINVSHYVTTFSFGKRLSSKMFNELKRLFPYVGGHHDRLAGQSYVVKHGDVNANVTIEHYLQIVKTELVTLRYSKELKVLEEYEYTAHSSLVHSFYVPVVKFHFEPSPMQVLVTELPKSFSHFITNVCAIIGGVFTVAGILDSILHNTLRLVKKVELGKDI*

>SbPDIL1-1 Sb05g006150.1

MAIRSSKACWISLLLALALSAVARAEEPAAEGAAEAVLTLDVDSFDEAVAKHPFMVVEFYAPWCGHCKKLAPEYETAAKELSKHDPPIVLAKVDANEEKNRPLATKYEIQGFPTLKIFRNQGKNIQEYKGPREADGIVDYLKKQVGPASKELKSQEDVATHYDDKNIYIVGVFTEFSGTEFTNFMEVAEKLRSDYDFGHTLHANHLPRGDAAVERPLVRVLKPFDELVVDTKDFDVAALLKFIDATTVPRVVTFDKNPDNHPYLMKFFQSSAPKAMLFLNFSTGPFDSFKSVYYAAAEEFQNKEIKFLIGDLESSQGALQYFGLKEDQAPLILIQDGDSKKFLKDQIEADQIVSWLKEYFDGKLTPFKKSEPIPEVNNEPVKVVVADNIHDFVFKSGKNVLIEFYAPWCGHCKKLAPILEEAATTLQSDEEVVIAKMDATANDVPSEFEVQGYPTMYFVTPSGKVTAYDSGRTADDIVDFIKKSKETAGATQATTTTSEKAADAAEKAEPVKDEL*

>SbPDIL1-2 Sb06g017160.1

MAVSLVLPFTILLAAVLLSGPVLAEVETAVELGEVVLTLDASNFSEVVAKHQFIVVEFYAPWCGHCKQLAPEYEKAAAVLRNHDPPLVLAKVDAYDERNKEIKDKYQVHAYPTIKIIENGGKDVRGYGGPRDADGIVGYLKKQVGPASIELSSAEAAQSSIGDKGVVLVGVFPEFAGVEYENFMAVAEKKRSDYDFFHTSDASILPRGDQTIKGPVVRLFKPFDELFADSRDFDTDALEKFIDVSGFPAVVTFDADPTNHKFLERYYSTPSAKAMLFLNFSDDRVEAFKNQIQEAAKKFSANNISFLIGDVEAADRAFQYGNLTPYVKSEPIPKVNDQPVKVVVADSIDDVVFNSGKNVLLEFYAPWCGHCRKLAPILEEVAVSLQDDEDVVIAKMDGTANDIPTDLAVEGYPTIYFYSTTGDLYSYNGGRTAEDIISFIKKNKGPRAGAVDEVTQTDAGAVEEGTAPSSTSELPKDEL*

>SbPDIL2-1 Sb04g000230.1

MGSTTMSLPSFPAVLMLLLLATAAAAAAGSNKAEEELDDLQYLIDNSHDIPANDPDGWPEGGGGGDDDDDDDLLFQDQDEDLLGHQPQIDETHVVVLTAANFSSFLSATRHVMVEFYAPWCGHCQELAPEYAAAAAHLAAHPHQADLALAKVDATEETDLAQRYDVQGFPTILFFIDGVPKDYNGARTKDAIVDWINKKLGPAVQNVTSVDEAERILTGDDKAVLAFLDTLSGAHSDELAAASRLEDSINFYQTLTPDVAKLFHIDAATKRPSIVLLKKEEEKLTFYDGEFKASAIADFVSANKLPLVTTLTQETSPSIFGNPIKKQILLFAIASESSKFLPIFKEAAKPFKGKLLFVFVERDNEEVGEPVADYFGITGQETTVLAYTGNEDAKKFFLDGEVSLEAIKDFAEGFLEDKLTPFYKSEPVPESNDGDVKMVVGKNLDLIVLDESKDVLLEIYAPWCGHCQSLEPTYNKLARHLRGVDSLVIAKMDGTANEHPRAKSDGYPTILFYPAGKKSFEPITFEGERTVVDMYKFIKKHASIPFKLKRQESSTQMEEGVKSSDTNLKDEL*

>SbPDIL3-1 Sb10g004440.1

MRARRVGPLLLLAVLALAAASSSARLDLDDDDDSGVLDDLLAIDEEPDRAGLDAAAGGAAEAVRRAQSMVLVLDNDNARRAVEDHAELLLLGYAPWCERSAQLMPRFAEAAAALRAMGSAVAFAKLDGERYPKAAAAVGVRGFPTVLLFVNGTEHAYQGLHTKDAIVTWVRKKTGVPVIRLQSKESAEEFLKKDQTFVIGLFKNFEGAEHEEFVKAATTDNEVQFVETSDTSVAKVLFPGITSVEKFVGLVKSEPEKFEKFDGEFEEKAILRFVELNKFPLITVFTELNSGKVYSSPIKLQVFTFSEAYDFEDLESMVEEIARAFKTKIMFIYVDTAEENLAKPFLTLYGLESEKRPTVTAFDTSNGAKYLMEADINAKNLREFCLSLLDGTLPPYHKSEPVPQEKGLVEKVVGRTFDSSVLESHQNVFLEVHTPWCVDCEAISKNVEKLAKHFNGLDNLKFARIDASVNEHPKLKVNNYPGLFLFLAEDKSKPIKLSKKSSVKDMAKLIKEKLQISDVETVAAPDNVKDDVETVAAPDSVKDEL*

>SbPDIL4-1 Sb09g004370.1

MASPQISRRALGLLLVLATAAAAVVSPAAADDVVALTEADFEKEVGQDRGALVEFYAPWCGHCKKLAPEYEKLGASFKKAKSVLIAKVDCDEHKGLCSKYGVSGYPTIQWFPKGSLEPKKYEGQRSVEALAEYVNSEAGTNVKIVAIPSSVVVLTPETFDSIVLDETKDVLVEFYAPWCGHCKHLAPVYEKLASVFKQDDGVVIANLDADKHTDLAEKYGVSGFPTLKFFPKGNKAGEDYDGGRDLDDFVKFINEKCGTSRDSKGQLNSEAGLVASLNPLVKEFLNAAADKRKEVISKIEEDVAKLSGSAAKHGKIYVTAAKKIMDKGSDYTKKETERLHRLLEKSISPSKADEFIIKKNILSTFSS*

>SbPDIL4-2 Sb03g013630.1

MAISQISRGTLSILLLLAAAFAAAPAALADGDDVVALTESTFEKEVGQDRGALVEFYAPWCGHCKKLAPEYERLGASFKKAKSVLIAKIDCDEHKSLCSKYGVSGYPTIQWFPKGSLEPKKYEGQRTAEALAEFVNTEGGTNVKLATIPSSVVVLTPETFDSIVLDEAKDVLVEFYAPWCGHCKSLAPTYEKVASVFKLDEGVVIANLDADKYRDLAEKYGVTGFPTLKFFPKGNKAGEDYDGGRDLGDFVKFINEKSGTSRDTKGQLTSEAGRIASLDVLAKEFLGASSDKRKEVLSSM

EEEAAKLSGPSARHGKVYVNIAKKILEKGNEYTKKETERLDRMLEKSINPSKADEFIIKKNVLSTFSS*

>SbPDIL5-1 Sb02g026300.1

MRSAVVAALLLVAAVASPAAALYSAGSPVLQLNPNNFKSKVLNSNGVVLVEFFAPWCGHCKQLAPAWEKAAGVLKGVATVAALDADAHQALAQEYGIRGFPTIKVFSPGKPPVDYQGARDVKPIVEFALSQVKSLLRERLSGKASAGSNGKTSGGSSEKSEPSASVELNSRNFDELVVKSKDLWIVEFFAPWCGHCKKLAPEWKKAAKNLKGQVKLGHVDCDAEKSLMSKYKVEGFPTILVFGADKESPFLYQGARVSSAIESFALEQLEANSGPAEVSELTGPDVMEEKCASAAICFVSFLPDILDSKAEGRNKYLELLLSVAEKFKKSPYSFVWTAAGKQANLENQVGVGGYGYPAMVALNVKKGAYTPLRSAFQRDEIIEFVKEAGRGGKGNLPLNGAPTVVTSEPWDGKDGEVIEEDEFSLDELMGDSSSVNDEL*

>SbPDIL6-1 Sb01g038630.1

MDLGATRRGRLPIHLLLASLTVLVVLTVRSSAEVITLTEETFSDKIKEKDTIWFVKFCVPWCKHCKNLGTLWEDLGKVMEGEDEIEIGQVDCGVSKPVCSKVDIHSYPTFKVFYEGEEVAKYKGHRDVESLKNFVLSEAEKAGEAKLQAD*

>SbPDIL7-1 Sb06g016990.1

MATRVLPPALLSLILLPLLLLSARDTVAAGEDFPRDGRVIDLDESNFEAALGAIDFLFVDFYAPWCGHCKRLAPELDEAAPVLAGLSEPIVVAKVNADKYRKLGSKYGVDGFPTLMLFIHGVPIEYTGSRKADQLVRNLKKFVSPDVSILESDSAIKTFVENAGTSFPMFLGFGVNDSLIAEYGRKYKKRAWFAVAKEFSEDIMVAYEFDKVPALVAIHPKYKEQSLFYGPFEENFLEDFVRQSLLPLVVPINTETLKMLNDDQRKVVLTILEDDSDENSTQLVKILRSAANANRDLVFGYVGIKQWDEFVETFDVSKSSQLPKLLVWDRNEEYELVDGSERLEEGDQASQISQFLEGYRAGRTTKKKISGPSFMGFLNSLVSLSSLYILIFVIALLVVMVYFAGQDDTPQPRRIHEE*

>SbPDIL7-2 Sb04g022420.1

MAMALRRRLLLLLPLLFLVVLVQRPHNCVASGGGGGEPAEFEIPRDGSVLELDESNFEAAVRAAEFLFVDFYAPWCGHCKRLAPQLDEAAVVLAGLSTPVVVAKVNADKYRKLGSKYGVDGFPTLMLFDHGVPSEYTGSRKADLLVENLKKLVAPDVSVLESDSSIKGFVEAAGINFPLFIGFGMDESLIVEYGAKYKKKAWFSTAKDFSEDMMVVYDFDKFPALVSVNPKYNEQSVFYDPFEVRNFSSLMVTPIVVLDTGTFLEDFIRQSLLPVTVPVDRETVKLLKDDGRKVVLTILE

DESDENSPQLIKVLRSAANANHDLVFGYVGVKQWEEFSETFDVKVPQLPKIIVWDTKEEYEVVEGSESLREGDYGSQVSRFLEGYREGRTIKKKVGRGSPTLLGLNAIYILIFLVAVLVVLMYFSAQGEEDHQPRRGRAHED*

>SbPDIL8-1 Sb02g035000.1

MISSSKLKSVDFYRKIPRDLTEASLSGAGLSIVAALAMVFLFGMELSNYLAVNTTTSVIVDRSSDGEFLRIDFNISFPALSCEFVSVDVSDVLGTNRLNIRKTVRKYSIDRNFVPTGSEFHPGPIPTVNKHGDDVEEDHVDGAFSLSSRNFDSFSHQYPVLVVNFYAPWCYWSNRLKPSWEKTAKIIRERYDPEMDGRILLGKVDCTEEVDLCRRHHIQGYPSIRVFRKGSDIKENQGHHDHESYYGERDTESLVAAMETYVANIPKEAHVLALEDKSNKTADPAKRPAPMTSGCRIEGFVRVKRVPGSVIVAARSGSHSFDPSQINVSHYVTQFSFGKRLSHRMLDEFTRLTPYLSGYNDRLAGQSYIVKHGEVNANVTIEHYLQVVKTEIVTQRSSKELKVLEEYEYTAHSSLVHSFYVPVVKFHVEPSPMQVLVTEVPRSFSHFITNVCAIIGGVFTVAGILDSIFHNTLRMVKKVELGKNI*

>AtPDIL1-1 AT1G21750.1

MAMRGFTLFSILVLSLCASSIRSEETETKEFVLTLDHTNFTDTINKHDFIVVEFYAPWCGHCKQLAPEYEKAASALSSNVPPVVLAKIDASEETNREFATQYEVQGFPTIKIFRNGGKAVQEYNGPREAEGIVTYLKKQSGPASAEIKSADDASEVVSDKKVVVVGIFPKLSGSEFDSFMAIAEKLRSELDFAHTSDAKLLPRGESSVTGPVVRLFKPFDEQFVDSKDFDGEALEKFVKESSIPLITVFDKDPNNHPYVIKFFESTNTKAMLFINFTGEGAESLKSKYREVATSNKGQGLSFLLGDAENSQGAFQYFGLEESQVPLIIIQTADDKKYLKTNVEVDQIESWVKDFKDGKIAPHKKSQPIPAENNEPVKVVVSDSLDDIVLNSGKNVLLEFYAPWCGHCQKLAPILDEVAVSYQSDSSVVIAKLDATANDFPKDTFDVKGFPTIYFKSASGNVVVYEGDRTKEDFISFVDKNKDTVGEPKKEEETTEEVKDEL*

>AtPDIL1-2 AT1G77510.1

MAFKGFACFSILLLLSLFVSSIRSEETKEFVLTLDHSNFTETISKHDFIVVEFYAPWCGHCQKLAPEYEKAASELSSHNPPLALAKIDASEEANKEFANEYKIQGFPTLKILRNGGKSVQDYNGPREAEGIVTYLKKQSGPASVEIKSADSATEVVGEKNVVAVGVFPKLSGDEFDSFMALAEKLRADYDFAHTLDAKFLPRGESVEGPAVRLFKPFDELFVDSKDFNGEALEKFVKESSIPLVTVFDSDPNNHPYVAKFFESPATKAMMFVNFTGATAEALKSKYREVATSNKDQSLAFLVGDAESSQGAFQYFGLEESQVPLIIIQTPDNKKYLKVNVEVDQIESWFKDFQDGKVAVHKKSQPIPAENNEPVKVVVAESLDDIVFKSGKNVLIEFYAPWCGHCQKLAPILDEVALSFQNDPSVIIAKLDATANDIPSDTFDVKGFPTIYFRSASGNVVVYEGDRTKEDFINFVEKNSEKKPTSHGEESTKSEEPKKTEETAAKDEL*

>AtPDIL2-1 AT3G54960.1

MASSSTSISLLLFVSFILLLVNSRAENASSGSDLDEELAFLAAEESKEQSHGGGSYHEEEHDHQHRDFENYDDLEQGGGEFHHGDHGYEEEPLPPVDEKDVAVLTKDNFTEFVGNNSFAMVEFYAPWCGACQALTPEYAAAATELKGLAALAKIDATEEGDLAQKYEIQGFPTVFLFVDGEMRKTYEGERTKDGIVTWLKKKASPSIHNITTKEEAERVLSAEPKLVFGFLNSLVGSESEELAAASRLEDDLSFYQTASPDIAKLFEIETQVKRPALVLLKKEEEKLARFDGNFTKTAIAEFVSANKVPLVINFTREGASLIFESSVKNQLILFAKANESEKHLPTLREVAKSFKGKFVFVYVQMDNEDYGEAVSGFFGVTGAAPKVLVYTGNEDMRKFILDGELTVNNIKTLAEDFLADKLKPFYKSDPLPENNDGDVKVIVGNNFDEIVLDESKDVLLEIYAPWCGHCQSFEPIYNKLGKYLKGIDSLVVAKMDGTSNEHPRAKADGFPTILFFPGGNKSFDPIAVDVDRTVVELYKFLKKHASIPFKLEKPATPEPVISTMKSDEKIEGDSSKDEL*

>AtPDIL2-2 AT5G60640.1

MAFRVLLLFSLTALLIFSAVSPSFAASSSDDVDDEDLSFLEDLKEDDVPGADSLSSSTGFDEFEGGEEEDPDMYNDDDDEEGDFSDLGNPDSDPLPTPEIDEKDVVVIKERNFTDVIENNQYVLVEFYAPWCGHCQSLAPEYAAAATELKEDGVVLAKIDATEENELAQEYRVQGFPTLLFFVDGEHKPYTGGRTKETIVTWVKKKIGPGVYNLTTLDDAEKVLTSGNKVVLGYLNSLVGVEHDQLNAASKAEDDVNFYQTVNPDVAKMFHLDPESKRPALVLVKKEEEKISHFDGEFVKSALVSFVSANKLALVSVFTRETAPEIFESAIKKQLLLFVTKNESEKVLTEFQEAAKSFKGKLIFVSVDLDNEDYGKPVAEYFGVSGNGPKLIGYTGNEDPKKYFFDGEIQSDKIKIFGEDFLNDKLKPFYKSDPIPEKNDEDVKIVVGDNFDEIVLDDSKDVLLEVYAPWCGHCQALEPMYNKLAKHLRSIDSLVITKMDGTTNEHPKAKAEGFPTILFFPAGNKTSEPITVDTDRTVVAFYKFLRKHATIPFKLEKPASTESPKTAESTPKVETTETKESPDSTTKSSQSDSKDEL*

>AtPDIL3-1 AT1G52260.1

MSLIPKPISKVSTFTFILLILLSFTIIIAYSSPDSNVESNEPGFDSDLDQLLAVDEQLQEDRPEQQSEAETVSKAQRIVLELNGDYTKRVIDGNEFVMVLGYAPWCARSAELMPRFAEAATALKEIGSSVLMAKIDGDRYSKIASELEIKGFPTLLLFVNGTSLTYNGGSSAEDIVIWVQKKTGAPIITLNTVDEAPRFLDKYHTFVLGLFEKFEGSEHNEFVKAAKSDDEIQFIETRDSDVAKLLFPDLKSNNVFIGLVKPEAERYTVYDGSYKMEKILEFLGSNKFPLFTKLTETNTVWVYSSPVKLQVMLFSKADDFQKLAQPLEDIARKFKSKLMFIYVDITNENLAMPFLILFGIEAGNKTVVAAFDNNLNSKYLLESDPSPNSIEEFCSGLAHGTVSRYYRSEPVPDNENASIVTVVGKTFDGLVLNSRENVLLEVHTPWCVNCEALSKQIEKLAKHFKGFENLVFARIDASANEHTKLQVDDKYPIILLYKSGEKEKPLKLSTKLSAKDIAVFINEELLKPKNGSAKDEL*

>AtPDIL3-2 AT3G16110.1

MLTKPKPNSKFSILFTFLLLLSFLIFVARSSDVAVEAGSEEELDDLEQLLAVDEQLQEERPEQQSEAETVSKAQRIVVELNGDNTKRLIDGNEYVMVLGYAPWCARSAELMPRFAEAATDLKEIGSSVLMAKIDGERYSKVASQLEIKGFPTLLLFVNGTSQSYTGGFSSEEIVIWVQKKTGASTIKLDTVDEASGFLKKHHTFILGLFEKSEDSSGHDEFVKAASLDNEIQFVETSSIDVAKLLFPNLKTNNVFVGLVKTEAEKYTSYDGPCQAEKIVEFLNSNKFPLVTKLTESNTVRVYSSPVKLQVMVFSKTDDFESLAQPLEDIARKFKSKLMLIYIDISNENLAMPFLTLFGIEDAKKTVVAAFDNNLNSKYLLESDPSPSNIEEFCFGLAHGTVSAYYKSQPIPDNQNASVVAVVGRTFDEVVLRSSENVLLEVHTPWCINCEALSKQVEKLSQHFKGFENLVFARIDASANEHPKLTVDDYPTILLYKTGEKENPLKLSTKSSAKDMAVLINKELKWKDQSGKDEL*

>AtPDIL4-1 AT2G47470.1

MAKSQIWFGFALLALLLVSAVADDVVVLTDDSFEKEVGKDKGALVEFYAPWCGHCKKLAPEYEKLGASFKKAKSVLIAKVDCDEQKSVCTKYGVSGYPTIQWFPKGSLEPQKYEGPRNAEALAEYVNKEGGTNVKLAAVPQNVVVLTPDNFDEIVLDQNKDVLVEFYAPWCGHCKSLAPTYEKVATVFKQEEGVVIANLDADAHKALGEKYGVSGFPTLKFFPKDNKAGHDYDGGRDLDDFVSFINEKSGTSRDSKGQLTSKAGIVESLDALVKELVAASEDEKKAVLSRIEEEASTLKGSTTRYGKLYLKLAKSYIEKGSDYASKETERLGRVLGKSISPVKADELTLKRNILTTFVASS*

>AtPDIL5-1 AT1G04980.1

MERKMYKSTVFPICCLLFALFDRGNALYGSSSPVLQLTPSNFKSKVLNSNGVVLVEFFAPWCGHCQSLTPTWEKVASTLKGIATVAAIDADAHKSVSQDYGVRGFPTIKVFVPGKPPIDYQGARDAKSISQFAIKQIKALLKDRLDGKTSGTKNGGGSSEKKKSEPSASVELNSSNFDELVTESKELWIVEFFAPWCGHCKKLAPEWKKAANNLKGKVKLGHVNCDAEQSIKSRFKVQGFPTILVFGSDKSSPVPYEGARSASAIESFALEQLESNAGPAEVTELTGPDVMEDKCGSAAICFVSFLPDILDSKAEGRNKYLEMLLSVADKFKKDPYGFVWVAAGKQPDLEKRVGVGGYGYPAMVALNAKKGAYAPLKSGFEVKHLKDFVKEAAKGGKGNLPIDGTMEIVKTEAWDGKDGEVVDADEFSLEDLMGNDDEASTESKDDL*

>AtPDIL5-2 AT2G32920.1

MYKSPLTLLTLLTICFGFFDLSSALYGSSSPVVQLTASNFKSKVLNSNGVVLVEFFAPWCGHCKALTPTWEKVANILKGVATVAAIDADAHQSAAQDYGIKGFPTIKVFVPGKAPIDYQGARDAKSIANFAYKQIKGLLSDRLEGKSKPTGGGSKEKKSEPSASVELNASNFDDLVIESNELWIVEFFAPWCGHCKKLAPEWKRAAKNLQGKVKLGHVNCDVEQSIMSRFKVQGFPTILVFGPDKSSPYPYEGARSASAIESFASELVESSAGPVEVTELTGPDVMEKKCGSAAICFISFLPDILDSKAEGRNKYLEMLLSVAEKFKKQPYSFMWVAAVTQMDLEKRVNVGGYGYPAMVAMNVKKGVYAPLKSAFELQHLLEFVKDAGTGGKGNVPMNGTPEIVKTKEWDGKDGELIEEDEFSLDELMGGDDAVGSKDEL*

>AtPDIL6-1 AT1G07960.1

MTLGARLVAPMIILLLFIPIELVKAEVITLTPETFSDKIKEKDTAWFVKFCVPWCKHCKKLGNLWEDLGKAMEGDDEIEVGEVDCGTSRAVCTKVEIHSYPTFMLFYNGEEVSKYKGKRDVESLKAFVVEETEKAAEKAQLEDKEL*

>AtPDIL7-1 AT1G35620.1

MRSLKLLLCWISFLTLSISISASSDDQFTLDGTVLELTDSNFDSAISTFDCIFVDFYAPWCGHCKRLNPELDAAAPILAKLKQPIVIAKLNADKYSRLARKIEIDAFPTLMLYNHGVPMEYYGPRKADLLVRYLKKFVAPDVAVLESDSTVKEFVEDAGTFFPVFIGFGLNESIISGLGRKYKKKAWFAVSKEVSEDTMVSYDFDKAPALVANHPTYNEHSVFYGPFEDGFLEEFVKQSFLPLILPINHDTLKLLKDDERKIVLTIVEDETHESLEKLYKALRAAAHANRDLVFGYVGVKQFEEFVDSFHVDKKTNLPKIVVWDGDEEYDQVTGIETITQEEDHLTQVSRFLEGYREGRTEKKKINGPSFMGFINSMIGIRSVYILVFLVAVIMMLRSLGQVEEPTGVRTATAVRERVDQATTVPEDESSEHKPSDKKED*

>AtPDIL8-1 AT3G20560.1

MVSSTKLKSVDFYRKIPRDLTEASLSGAGLSIVAALFMMFLFGMELSSYLEVNTTTAVIVDKSSDGDFLRIDFNISFPALSCEFASVDVSDVLGTNRLNITKTVRKFPIDPHLRSTGAEFHSGLALHNINHGEETKEEFPDGAIPLTSASFEALSHHFPILVVNFNAPWCYWSNRLKPSWEKAANIIKQRYDPEADGRVLLGNVDCTEEPALCKRNHIQGYPSIRIFRKGSDLREDHGHHEHESYYGDRDTDSIVKMVEGLVAPIHPETHKVALDGKSNDTVKHLKKGPVTGGCRVEGYVRVKKVPGNLVISAHSGAHSFDSSQMNMSHVVSHFSFGRMISPRLLTDMKRLLPYLGLSHDRLDGKAFINQHEFGANVTIEHYLQTVKTEVITRRSGQEHSLIEEYEYTAHSSVAQTYYLPVAKFHFELSPMQILITENPKSFSHFITNLCAIIGGVFTVAGILDSIFHNTVRLVKKVELGKNI*

>AtPDIL8-2 AT4G27080.2

MVSTSKIKSVDFYSDPEVVGFASRVCALSLSFDLSDIELFRCYFLGFKMIVSHFDFGIVAKRVASKKIPRDLTEASLSGAGLSIIAALSMIFLFGMELNNYLAVSTSTSVIVDRSADGDFLRLDFNISFPSLSCEFASVDVSDVLGTNRLNVTKTIRKFSIDSNMRPTGSEFHAGEVLSLINHGDETGEEIVEDSVPLTGRNFDTFTHQFPILVVNFYAPWCYWCNLLKPSWEKAAKQIKERYDPEMDGRVILAKVDCTQEGDLCRRNHIQGYPSIRIFRKGSDLKDDNAHHDHESYYGDRDTESLVKMVVSLVEPIHLEPHNLALEDKSDNSSRTLKKAPSTGGCRVEGYMRVKKVPGNLMVSARSGSHSFDSSQMNMSHVVNHLSFGRRIMPQKFSEFKRLSPYLGLSHDRLDGRSFINQRDLGPNVTIEHYLQIVKTEVVKSNGQALVEAYEYTAHSSVAHSYYLPVAKFHFELSPMQVLITENSKSFSHFITNVCAIIGGVFTVAGILDSILHHSMTLMKKIELGKNF*

>GmPDIL1-1 Glyma04g42690.1

MPKFFHSIFRGTMAGRVSTCFFFVFALSLLLPFQISAEESSEKEFVLTLDHSNFHDTVSKHDFIVVEFYAPWCGHCKKLAPEYEKAASILSSHDPPVVLAKIDANEEKNKDLASQYDVRGYPTIKILRNGGKNVQEYKGPREADGIVDYLKKQSGPASTEIKSADEATAFIGENKVAIVGVFPKFSGEEFDNFSALAEKLRSDYDFGHTLNAKHLPRGESSVSGPVVRLFKPFDELFVDFQDFNVEALEKFVEESSTPVVTVFNNDPSNHPFVAKFFNSPNAKAMLFINFTAEGAESFKSKYREAAEQHKQQGVSFLVGDVESSQGAFQYFGLKEEQVPLIIIQHNDGKKFFKPNLEADHIPTWLKAYKDGNVAPFVKSEPIPEANDEPVKVVVGNSLEDIVFKSGKNVLLEFYAPWCGHCKQLAPILDEVAISYQSDADVVIAKLDATANDIPSETFDVQGYPTVYFRSASGKLSQYEGGRTKEDIIEFIEKNRDKPAQQEQGQDKPAQQEQGQDEQEKGKDEL*

>GmPDIL1-2 Glyma06g12090.1

MAGTVSTCFFFVFLLSLLLPFQISAEESSEKEFVLTLDHSNFHDTVSKHDFIVVEFYAPWCGHCKKLAPEYEKAASILSSHDPPIVLAKVDANEEKNKDLASQYDVKGFPTINILRNGGKNVQEYKGPREADGIVDYLKKQSGPASTEIKSADEATAFIGENKVAIVGVFPKFSGEEFDNFSALAEKLRSDYDFGHTLNAKLLPRGESSVSGPVVRLFKPFDELFVDFQDFNVEALEKFVEESSTPVVTVFNNEPSNHPFVVKFFNSPNAKAMLFINFTAEGAEAIKSKYREAAEQYKQQGVSFLVGDVESSQGAFQYFGLKEEQVPLIIIQHNDGKKFFKPNLEADHIPTWLKAYKDGHVAPFVKSEPIPETNDEPVKVVVGASLEDIVFKSGKNVLLEFYAPWCGHCKQLAPILDEVAISYQNEADVVIAKLDATANDIPSETFDVQGYPTVYFRSASGKLSQYDGGRTKEDIIEFIEKNRDKPAQQEQGKDEQEQGKDEL*

>GmPDIL2-1 Glyma12g29550.1

MRILVVLSLATLLLFSSLFLTLCDDLTDDEDLGFLDEPSAAPEHGHYHDDDANFGDFEEDPEAYKQPEVDEKDVVILKEKNFTDTVKSNRFVMVEFYAPWCGHCQALAPEYAAAATELKGEDVILAKVDATEENELAQQYDVQGFPTVYFFVDGIHKPYNGQRTKDAIMTWIKKKIGPGIYNLTTVEDAQRILTNETKVVLGFLNSLVGPESEELAAASRLEDDVNFYQTVDPDVAKLFHIDPDVKRPALILVKKEEEKLNHFDGKFEKSEIADFVFSNKLPLVTIFTRESAPSVFENPIKKQLLLFATSNDSEKLIPAFKEAAKSFKGKLIFVYVEMDNEDVGKPVSEYFGISGNAPKVLGYTGNDDGKKFVLDGEVTADKIKAFGDDFLEDKLKPFYKSDPVPESNDGDVKIVVGNNFDEIVLDESKDVLLEIYAPWCGHCQALEPIYDKLAKHLRNIESLVIAKMDGTTNEHPRAKPDGFPTLLFFPAGNKSFDPITVDTDRTVVAFYKFLKKHASIPFKLQKPTSTSDAKGSSDAKESQSSDVKDEL*

>GmPDIL2-2 Glyma13g40130.1

MRILVLLSLATLLLFSSFSPTFCDHLADDEDLSFLDEPSAAPEHDHHYGADDSNFGDFEDFEEDDAEAYKQPEVDEKDVVVLKEKNFTDAVKNNRFVMVEFYAPWCGHCQALAPEYAAAATELKGEDVILAKVDATEENELAQQYDVQGFPTVHFFVDGIHKPYNGQRTKDAIVTWIRKKIGPGIYNLTTVEEAQRILTNETKVVLGFLNSLVGPESEELAAASRLEDDVNFYQTVNPDVAKLFHIDQDVKRPALILIKKEEEKLNHFDGKFEKSAIADFVFSNKLPLVTIFTRESAPSVFENPIKKQLLLFATSNDSETLVPAFKEAAKSFKGKLIFVYVEMDNEDVGKPVSEYFGISGNAPKVLGYTGNDDGKKFVLDGEVTTDKIKAFGEDFVEDKLKPFYKSDPVPESNDGDVKIVVGNNFDEIVLDESKDVLLEIYAPWCGHCQSLEPIYNKLAKHLRNIDSLVIAKMDGTTNEHPRAKPDGFPTLLFFPAGNKSFDPITVDTDRTVVAFYKFLKKHASIPFKLQKPTSTSESDSKGSSDAKESQSSDVKDEL*

>GmPDIL2-3 Glyma12g07260.1

MLSRKRLIVSLSLATLLLFSSLSLTLCDKIPPQNDNNNDDDEDLSFLEEPDDAAATSHHGHFPDPDRFDEDGDDDGDFGDFSGFDHSTEEAFEVDDKDVVVLKERNFTTVVENNRFIMVEFYAPWCGHCQALAPEYAAAATELKPDGVVLAKVDATVENELANEYDVQGFPTVFFFVDGVHKPYTGQRTKDAIVTWIKKKIGPGVSNITTVDDAERILTAESKVVLGLLNSLVGTESDELAAASKLEDDVNFYQTVVADVAKLFHIDPSVKRPALILLKKEEEKLNHFDGQFVKAEIADF

VTSNKLPLVTIFTRESAPVIFESQIKKQLLLFVTSNDTEKFVPVFKEAAKKFKGKLIFVHVELDNEDVGKPVADYFGITGNGPKVLAYTGNDDGRKFLLDEELTVDTITAFGNDFLEEKLKPFLKSDPVPESNDGDVKIVVGNNFDEIVLDESKDVLLEIYAPWCGHCQALEPTYNKLAKHLRNIESIVIAKMDGTTNEHPRAKSDGFPTLLFFPAGNKSSDPIPVDVDHTVKAFYKFLRKHASIPFQLQKPTSTAKTGSESSYVKESQSSSTDVKDEL*

>GmPDIL2-4 Glyma11g20630.1

MLTRKRLVVSLSLATLLLFSSLSLSLCDKTPPQNDNKKNNNDDDEDLSFLEESDDAATTSHQGHFPDPDEFDEDDGDDEDDFGDFAGFDHSSEEAFKEPEVDDKDVVVLKERNFTTVVENNRFVMVEFYAPWCGHCQALAPEYAAAATELKPDGVVLAKVDATVENELANEYDVQGFPTVFFFVDGVHKPYTGQRTKDAIVTWIKKKIGPGVSNITTVEEAERVLTAGSKVVLGFLNSLVGAESDELAAASKLEDDVNFYQTVVADVAKLFHIDASVKRPALILLKKEEEKLNHFDGQFVKAEIADFVTSNKLPLVTTFTRESAPVIFESQIKKQLLLFVTSNDTEKFVPVFKEAAKIFKGKLIFVHVESDNEDVGKPVADYFGIAGNGPKVLAFTGNDDGRKFLLDGEVTIDTITAFGNDFLEDKLKPFLKSDPVPESNDGDVKIVVGNNFDEIVLDESKDVLLEIYAPWCGHCQALEPTYNKLAKHLRSIESIVIAKMDGTTNEHPRAKSDGFPTLLFFPAGNKSSDPIPVDVDRTVKDFYKFLRKHASIPFQLQKLASTTKTASESSDVKESQSSTTEVKDEL*

>GmPDIL3-1 Glyma15g01880.1

MCTMKPTLRFILVLLTLLLVLRFNVASEVEDELEELLAVDEEVEQEAEKGGEKLSEAEVLSKAQRIVIELNNDNTERVVNGNEFVLVLGYAPWCPRSAELMPHFAEAATSLKELGSPLVLAKLDADRYSKPASFLGVKGFPTLLLFVNGTSQPYSGGFAADDIVIWAQKKTSTPVIRIGSVTEAEKFLRKYQTFLIGRFDKFEGPDYEEFVSAAQSDNEIQFVETNQVELAQVLYPDIKPTDQFLGIVKSEPERYTAYDGAFTMNKILEFVDYNKFPLVTKLTEMNSIRVYSSPIKLQVLVFANIDDFKNLLETLQDVAKTFKSKIMFIYVDINDENLAKPFLTLFGLEESKNTVVAAFDNAMSSKYLLETKPTQSNIEEFCNNLVQGSLSPYFKSQPIPDNTESSVHVIVGKTFDDEILSSEKDVLLEVFTPWCINCEATSKQVEKLAKHYKGSSNLIFARIDASANEHPKLQVNDYPTLLLYRADDKANPIKLSTKSSLKELAASINKYVKVKNQVVKDEL*

>GmPDIL3-2 Glyma13g43430.1

MKPTLRFIVFLLTLLLVLRFNVATEVKDELEELLAVDEEVEREAEKGGEKLSEAEVLSKAQRIVIELKNENTERVVNGNEFVLVLGYAPWCPRSAELMPHFAEAATSLKELGSPLIMAKLDADRYPKPASFLGVKGFPTLLLFVNGTSQPYSGGFTADDIVIWAQKKTSTPVIRISSVAEAEKFLTKYQTFLIGRFENFEGPDYEEFVSAAKSDNEIQFVETSQVELAQVLYPDIKPTDRFLGIVKSEPERYSAYDGAFILNKILEFVDYNKFPLVTKLTEMNSVRVYSSPIKLQVLVFANIDDFKNLLDTLQDVAKTFKSKIMFIYVDINDENLAKPFLTLFGLEESKNTVVSAFDNSMSSKYLLESKPTQINIEEFCNNLMQGSLSPYFKSQPIPDNTEASVRAIVGKTFDDEILSSKKDVLLEVFTPWCMNCEATSKQVEKLAKHYKGSSNLIFARTDASANEHPKLQVNDYPTLLFYRADDKANPIKLSTKSSLKELAASINKYLKVKNQVLKDEL*

>GmPDIL4-1 Glyma02g01750.1

MEMYQIWSRRIALGAFAFVLLFLSASADDVVVLSEDNFEKEVGQDRGALVEFYAPWCGHCKKLAPEYEKLGSSFKKAKSVLIGKVDCDEHKSLCSKYGVSGYPTIQWFPKGSLEPKKYEGPRTADSLAEFVNTEGDLLAGTNVKIATAPSNVVVLTSENFNEVVLDETKDVLVEFYAPWCGHCKSLAPTYEKVATAFKLEEDVVIANLDADKYKDLAEKYDVSGFPTLKFFPKGNKAGEEYGGGRDLDDFVAFINEKSGTSRDVKGQLTSQAGIVESLDVLVKEFVAASDEEKKFVFTRMEEEVEKLKGSASRHGKIYLKAAKNYLEKGSDYAKNEIQRLQRILDKSISPAKADELTLKKNILSTYAA*

>GmPDIL4-2 Glyma19g41690.1

MWSSKTTMMLAIAAIALMMFLSSASADDVVALTEETFENEVGKDRAALVEFYAPWCGHCKRLAPEYEQLGASFKKTKSVLIAKVDCDEHKSVCGKYGVSGYPTIQWFPKGSLEPKKYEGARTAEALAAFVNIEAGTNVKIASVASSVVVLSPNNFDEVVFDETKDVLVEFYAPWCGHCKALAPIYEKVAAAFNLDKDVVIANVDADKYKDLAEKYGVSGYPTLKFFPKSNKAGENYDGGRDLDDFVAFINEKCGTYRDGKGQLTSKAGIIASLDDLVKEFVSADSNEKKAVYSRLEEEVKKLKGSSARHGDLYLKLAKKGMEKGADYAKNEIQRLERMLEKSVSPAKADEFTLKKNILSIFA*

>GmPDIL4-3 Glyma10g01820.1

MTPPEIEKKKKKKMEKYQIWSRRIALAAFAFALLFQSASADDVVVLSEDNFEKEVGQDRGALVEFYAPWCGHCKKLAPEYEKLGSSFKKAKSVLIGKVDCDEHKSLCSKYGVSGYPTIQWFPKGSLEAKKYEGPRTAESLVEFVNTEGGTNVKIATVPSNVVVLTPENFNEVVLDEAKDVLVEFYAPWCGHCKSLAPTYEKVATAFKLEEDVVIANLDADKYRDLAEKYDVSGFPTLKFFPKGNKAGEDYGGGRDLDDFVAFINEKSGASRDGKGQLTSQAGIVESLDVLVKEFVAASDEEKKSVFTRLEEEVVKLKGSASRYGKIYLKAAKNYREKGSDYAKNEIQRLQRILDKSISPAKADELTLKKNILSTYAA*

>GmPDIL4-4 Glyma03g39130.1

MWSSKTTLMFGVAAIALMMFLSSASADDVVALTEETFENEVGKDRAALVEFYAPWCGHCKRLAPEYEQLGTTFKKTKSVLIAKVDCDEQKSVCSKYGVSGYPTIQWFPKGSLEPKKYEGARTAEALAAFVNIEAGTNVKIASVPSSVVVLSPDNFDEVVLDETKDVLVEFYAPWCGHCKALAPIYEKVAAAFNLDKDVVMANVDADKYKDLAEKYGVSGYPTLKFFPKSNKAGEDYNGGRDLDDFVAFINEKCGTYRDGKGQLTSKAGIIASLDDLVKEFVSADSNEKKAVYSRLEEEVKKLKGSSARHGDLYLKLAKKGIEKGADYAKNEIQRLERMLEKSISPAKADEFTLKKNILSTFA*

>GmPDIL5-1 Glyma14g05520.1

MPKSQFRTPFLVSFPLLLFIFNLTPSHALYGASSPVLQLTPSNFKSKVLNSNGVVLVEFFAPWCGHCQALTPIWEKAATVLKGVVTVAAIDADAHPSLAQEYGIRGFPTIKVFAPGKPPVDYQGARDVKPIAEFALQQVKALLKDRLSGKATGGSSDKTETSSSVELNSGNFDELVIKSKELWIVEFFAPWCGHCKKLAPEWKKASNSLKGKVKLGHVDCDAEKSLMSRFKVQGFPTILVFGADKDSPIPYEGARTALAIESFALEQLETNVAPPEVTELHSPDVLEEKCGSAAICFVAFLPDILDSKAEGRNIYLQQLLSVAEKFKRSPYSYVWVAAGNQPDLEKNVGVGGYGYPALVALNLKKAVYAPLKSAFELDQIIEFVKEAGRGGKGNLPLQGTPTIVKTEPWDGKDGEIIEEDEFSLEELMGEDASSKDEL*

>GmPDIL5-2 Glyma02g43460.1

MPKSQFRTPFLVSFSLLLFIFNLTPSYALYGASTPVLQLTPSNFKSKVLNSNGVVLVEFFAPWCGHCQALTPIWEKAATVLKGVVTVAAIDADAHPSLAQEYGIRGFPTIKVFAPGKPPVDYQGARDVKPIAEFALQQVKALLKDRLSGKATGGSSEKTETSSSVELNSGNFDELVIKSKELWIVEFFAPWCGHCKKLAPEWKKASNNLKGKVKLGHVDCDAEKSLMSRFKVQGFPTILVFGADKDSPIPYEGARTASAIESFALEQLETNIAPPEVTELYSPDVLEEKCGSAAICFVAFLPDILDSKAEGRNRYLQQLLSVAEKFKRSPYSYVWVAAGKQPDLEKNVGVGGYGYPALVALNLKKAVYAPLKSAFELDQIIEFVKEAGRGGKGNLPIEGTPTIVKTEPWDGKDGEIIEEDEFSLEELMGEDASSKDEL*

>GmPDIL6-1 Glyma15g05050.1

MRTHTIISLILLSLFLRTQSEVITLSSDTFNDKIKEKDTAWFVKFCVPWCKHCKNLGSLWDDLGKAMEGEDEIEVGEVDCGMDKAVCSKVDIHSYPTFKVFYDGEEVARYQGTRDVESMKTFVLEEAEKAAAKALESDKEL*

>GmPDIL6-2 Glyma13g40350.1

MRTHTHTYTYTIFSLILLSLFLRTHSEVITLTSDTFNDKIKEKDTAWFVKFCVPWCKHCKNLGSLWDDLGKAMEGEDEIEVGEVDCGMDKAVCSKVDIHSYPTFKVFYDGEEVARYQGTRDVESMKTFVLEEAEKAAAKALESNKEL*

>GmPDIL7-1 Glyma10g36170.1

MRMKRVFVVYSVLLLLVQFWLGEAETFSVDGKVLVLDESNFDSAIASFDHILVDFYAPWCGHCKRLSPELDAAAPVLATLKEPIIIAKVDADKHTRLAKKYDVDAYPTILLFNHGVPTEYRGPRKADLLVRYLKKFSASDVSILDSDSAVNMFVEEAGTFFPIYIGFGLNSSVLEKFGIKYKKNAWFSVAKDFSEDLMVLHDFDKIPALVSLNPQYNERNTFYGPFEEDFLEDFVRQNLIPLAVPVSYETLKLMKADGRKIVLTIVEDEDEETTRELIKLLKAAASANRDLIFGYVGVKQMEEFAENFDIDTKLPKMVIWDKSDDYLSVVDSETIEGEDQGTQITKFLEGYREGRTIKKTFSGPSLMRFIHRSFDIRMVYIIVFVVAVLMLIQTFSKGGDEYQSVPNQVQTDHAISSVSEAENNEYKPGDKED*

>GmPDIL8-1 Glyma12g34090.1

MISSSKIKSVDFYRKIPRDLTEASLSGAGLSIVAALAMIFLFGMELNSYLSVSTSTQVIVDKSSDGDYLRIDFNISFPALSCEFAAVDVSDVLGTNRLNLTKTVRKFSIDSNLRPTGAEFHSEPAANSIKHDNEVNEESVEGSVVLTTQNFDKYAHQFLITVVNFYAPWCYWSQRLKPSWEKTAKIIKERYDPEMDGRIILGRVDCTEDGDLCRSHHIQGYPSIRIFRKGSDVRSDHGHHDHESYYGDRDTDSLVKTMENLVASLPSESQKLPLEDKSNVATNTKRPAPSTGGCRIDGYVRVKKVPGNLIISARSNAHSFDASQMNMSHVINHLSFGRKVSLRVMSDVKRLIPYVGSSHDRLNGRSFINTHDLGANVTIEHYLQIVKTEVITRKEYKLVEEYEYTAHSSVAQSLHIPVAKFHLELSPMQVLITENQKSFSHFITNVCAIIGGIFTVAGIMDAIFHNTIRLMKKVELGKNF*

>GmPDIL8-2 Glyma13g36450.1

MISSSKIKSVDFYRKIPRDLTEASLSGAGLSIVAALAMIFLFGMELNSYLSVTTSTQVIVDKSSDGDYLRIDFNISFPALSCEFAAVDVSDVLGTNRLNLTKTVRKFSIDSNLRPTGAEFHSEPAANSIKHDNEVNEESVEGSVVLKTQNFDKYAHQFPITVVNFYAPWCYWSQRLKPSWEKAAKIIKERYDPEMDGRIILGRVDCTEDGDLCRSHHIQGYPSIRIFRKGSDVRSNHGHHDHESYYGDRDTDSLVKTMENLVASLPSESQKLPLEDKSDVAKNTERPAPSTGGCRIDGYVRVKKVPGNLIFSARSNAHSFDASQMNMSHVINHLSFGRKVSPRVMSDVKRLIPYVGSSHDRLNGRSFINTHDLGANVTMEHYLQIVKTEVITRKDYKLVEEYEYTAHSSVAQSLHIPVAKFHLELSPMQVLITENQKSFSHFITNVCAIVGGIFTVAGIMDAILHNTIRLMKKVELGKNF*

>GmPDIL8-3 Glyma06g42130.1

MISATKLKSVDFYRKIPRDLTEASLSGAGLSIVAALAMMFLFGMELSSYLSVSTSTSVIVDKSSDGDYLRIDFNISFPALSCEFASVDVSDVLGTNRLNITKTVRKFSIDSNLRPTGAEFHSGTVANAVKHDDEVDEESVEGSFSLTTHNFDKYVHQFPITAVNFYAPWCSWCQRLKPSWEKTAKIMKERYDPEMDGRIILAKVDCTQEGDLCRRNHIQGYPSIRIFRKGTDLRSEHGHHEHESYYGDRDTESLVKFMEDLVTSLPTESQKLALEDKSNASDNAKRPAPSAGGCRVEGYVRVKKVPGNLIISARSDAHSFDASQMNMSHFINNLSFGKKVTPRAMSDVKLLIPYIGSSHDRLNGRSFTNTHDLGANVTIEHYIQIVKTEVVTRNGYKLIEEYEYTAHSSVAHSVDIPAAKFHLELSPMQVLITENQRSFSHFITNVCAIIGGVFTVAGILDSILHNTIRMMKKVELGKNF*

>GmPDIL8-4 Glyma12g16311.1

MISATKLKSVDFYRKIPRDLTEASLSGAGLSIVAALVMMFLFGMELSSYMSVSTSTSVIVDKSSDGDYLRIDFNIRLNITKTVRKFSMDSNLRPTGAEFHSGTVANAVKHDDEVDEESVEGSVSLTTHNFDKYVHQFPVTIVNFYAPWCSWNHIQGYPSIRIFRKGSDLRSEHGHHEHESYYGDRDTESLVKFMEDLVTSLPTESQKLALEDKSNAADNAKRPAPSAGGCRVEGYVRVKKVPGNLIISARSDAHSFDASQMNMSHVINNLSFGKKVTPRAMSDVKLLIPYIGSSHDRLNG

RSFINTRDLGANVTIEHYIQIVKTEVVTRKGYKLIEEYEYTAHSSVAHSLDIPVAKFHLELSPMQVLITENQRSFSHFITNVCAIIGGVFTVAGILDSILHNTIRMVKKIELGKNF*

>PtPDIL1-1 Potri.002G082100.1

MASTVSFWSCIFLLSLIVALSAGEDESKEYVLTLDHSNFNETVSKHDFIVVEFYAPWCGHCKKLAPEYEKAASILSSNDPQVVLAKVDANEDANKEIASQ

YDVKGFPTIVILRKGGKSVQEYKGPREADGIVEYLKKQSGPASAELKSDDDATGFIGDKKVVIVGVFPKFSGEEFENFLAVAEKLRSDYEFGHTLDAKYL

PRGESSVSGPLVRLFKPFDELFVDSKDFNVDALEKFVEESSIPIVTLFNKDPSNHPFVVKYFDSPLAKAMLFMNFSSENGDSIRTKYQEVAGLHKGDGLV

FLLGDVEASQGALQYFGLKEDQVPLIVIQTTDGQKYLKPNLVSDQIAPWLKEYKEGKVPPFKKSEPIPEVNDEPVKVVVADSLDELVTKSGKNVFLEFYA

PWCGHCQKLAPILEEVAISFQSDADVVIAKLDATANDIPSDTYDVKGFPTIFFRSATGKLVQYEGDRTKQDIIDFIEKNRDKIGQQEPAKEEEPAKEQET

AKDEL*

>PtPDIL1-2 Potri.005G179000.1

MMASKVSLWSCIFVFSLVVALSTGEDESKEYVLTLDHSNFTETVTKHDFVVVEFYAPWCGHCQNLAPEYEKAASILSSNDPQIVLAKVNADEKVNQEISE

KYEVQGFPTIKILRKGGTSVNEYKGPRDADGIAEYLKKQTGPASAELKSADDATSFIGDNKVVIVGVFPKFSGEEFESFLAVADKLRSDYEFAHTLDAKH

LPRGESSVSGPLVRLFKPFDELFVDSKDFNVDALEKFIEESSAPIVTVYDDEPSNHPYIVKYFDSPLDKAMLFLNFSGDSADSIKTNYQEVAEQHKGDGL

IFLLGDLEASQSALQYFGLKEDQAPLLVIQTTDGKKYLKSNLESDHIAPWVKEYKEGKVPPFIKSEPIPEANEEPVKVVVADSLDDLVTKSGKNVLLEFY

APWCGHCQKLAPILEEIAVSYQSDADVLLAKLDATANDIPGDTYDVKGFPTVYFRSASGKLVQYEGDKTKQDIIDFIEKNRDKVAQQEPAKDEL*

>PtPDIL2-1 Potri.009G013600.1

MSTRFIFLLSLTALLLFSHLSPSLSKLQNAAAEDDDEDLSFLEEETDAVPHGQGHGHDHDHDHYPDPDQFDEEFDNEDDLDNYSDLDDSELDSYKEPEID

DKDVVVLKEGNFSDFVTKNKFVMVEFYAPWCGHCQSLAPEYAAAATELKAEEVMLAKVDATEENELAQEYDIQGFPTVYFFVDGVHRPYPGPRNKDGIVT

WIKKKIGPGIYNITTVDDAERLLTSETKLVLGFLNSLVGPESEELAAASRLEDEVSFYQTVNPDVAKLFHLDPQAKRPALVMLKKEAEKLSVFDGNFSKS

EIAEFVFANKLPLVTIFTRESAPLIFESTIKKQLLLFAISNDSEKVVPIFQEAARLFKGKLIFVYVEMDNEDVGKPVSEYFGISGTAPKVLAYTGNDDAK

KFVFDGDVTLDKIKAFGEDFIEDKLKPFFKSDPVPESNDGDVKIVVGNNFDEIVLDESKDVLLEIYAPWCGHCQSLEPTYNKLATHLRGIESIVIAKMDG

TTNEHPRAKSDGFPTLLFFPAGNKSFDPITVDTDRTVVAFYKFIKKHASIPFKLQKPASASKAESSDAKDGIESSTRDVKDEL*

>PtPDIL3-1 Potri.001G183500.1

MFPAKPTSRSMLFTFTILLLLSSTIFVTANEDPTVETDNDGADSDLQELIAIDEQEGGGGEEQQQGDQQKEAEVLSKAQRIVLELNSDNARRVIDQNEFVLILGYAPWCARSAELMPQFAEAANKLKELGSPVLMAKLDAERYPKVASTLGIKGFPTLLLFVNGTSQV

YTGGFSGEDIVIWARKKTGVPVIRISSSVEAEDFQKKYHLFVLGLFDKFEGHDYEEFIKAATIDNEIQFVEVSSSAVAKILFPNINAKDNFIGIVKSEPE

KYTAYGGIFEKDTILQFLEYNKFPLVTILTELNSARVYSSPVKLQVIVFADADDFKNLIRPLQEVARKFISKIMFIYIDIADENQAKPFLTLFGIEDSEN

TVVTAFDNRMSSKYLLESNPTSSNIEEFCSRLLHGSLSPYFKSQPIPDNKEKILQVVVGKTLDDLVLSSPKNVLLEVYTPWCISCETTTKQIEKLAKHFK

GVDNLVFARIDASANEHPKLLVDDYPTLLFYPVGDKENPVKLSTKSSSKDLATVIKSLLRAKEDVPKDEL*

>PtPDIL4-1 Potri.002G198300.1

MERCNQIWYAFGTMALLAVSALADDVVVLTEDNFEKEVGQDKGALVEFYAPWCGHCKKLAPEYEKLGSSFKKAKAVLIGKVDCDEHKGVCSKYGVSGYPT

LQWFPKGSLEPKKYEGPRTAEALAEFVNNEGGSNVKIAAVTSSVVVLTADNFNDIVLDENKDVLVEFYAPWCGHCKNLAPIYEKVATAFKSEEDVVVANL

EADKYRDLAEKYGVSGFPTLKFFPKGNKAGEEYEGGRDLDDFVAFINEKAGTSRDGKGQLTSKAGIVESLDALVKEFVAAGDDEKKAVFSRIEEEVEKLK

GSTARHGKIYLKAAKTCMVKGAGYAKNEIERLQRMLEKSISPAKADEFTLKKNILSTFA*

>PtPDIL4-2 Potri.014G122800.1

MEKYHQIWFAFGTLALLAVSALADDVVVLTEDNFEKEVGQDRGALVEFYAPWCGHCKKLAPEYEKLGSSFRKAKTVLIGKVDCDEHKGVCSKYGVSGYPT

LQWFPKGSLEPKKYEGPRTAEALTEYVNTEGGTNVKIAAVPSNVAVLTADNFNNIVLDETKDVLVEFYAPWCGHCKNLAPTYEKVATAFKSEEDVVVANL

DADKHKDLAEKYGVSGFPTLKFFPKGNKAGEDYEGGRDLDDFVAFINEKSGSSRDGKGQLTSKAGIVESLDALVKEFVAAGDDEKKAVFSQIEEEVEKLK

GSAARYGKIYSKAAKNCMAKGDYAKNEIERLQRMLQKTISPAKADEFTLKKNILSTFA*

>PtPDIL5-1 Potri.014G160000.1

MRTQSELLLALSILFFQSNLFCYALYGPSSPVLQLNPSNFKSKVLNSNGVVLVEFFAPWCGHCKALTPTWEKAAAVLKGVATVAALDADAHQSLAQEYGI

RGFPTIKVFVPGNPPVDYQGARDVKPIAEYALKQIKALLKDRLNGKSTGGSSEKSETSLSVELNSRNFDELVLKSKELWIVEFFAPWCGHCKKLAPEWTK

AANNLQGKVKLGHVDCDSEKSLMSRFNVQGFPTILVFGADKDTPIPYEGARTASAIESFALEQLESNVAPPEVTELTGPDVMEEKCGSAAICFVAFLPDI

LDSKAEGRNKYLEQLLSVAEKFKRSPYSYVWAAAGKQPDLENRVGVGGYGYPALVALNAKKGAYAPLKSAFELEHIVEFVKEAGRGGKGNLPLNGNPEIV

KTEPWDGKDGEIIEEDEFSLEELMGEDAGSKDEL*

>PtPDIL6-1 Potri.009G004500.1

MINLNRSVSIWIVLSLFLSLFLSKAEVITLTPETFSDKVKEKDTAWFVKFCVPWCKHCKNLGTLWEEVGKAMEGEDEIEVGEVDCGASKSVCSKADIHSY

PTFKLFFDGEEVAKYQGPRDVESLKAFVLDEAEKAAAKAQLGYDKDL*

>PtPDIL7-1 Potri.019G082400.1

MKTRRSPLILLNTTPLLLLVLLSSSIISSAESTTPPEKINTVLELDESNFDSTISTYDYVFVDFYAPWCGHCKRLAPELDVAAPILAELKKPIVIAKVNA

DKYTRLARKHEVDGFPTLKIYMHGVPTDYYGPRKAELLVCFLRKFVAPDVTILNSDSAIREFVEEAGTHFPIFIGFGLNETVMSNLAIKYKKKAWFSVAS

DFSDDVMVQYDFDKIPALVSIHPSYNDHTVFYGPFEEEFMEEFITQNSLPLAVPINSETLKVLKDDQRKIVLTILEDDSEEKSQNLIKILKAAASANRDL

VFGFVGVKQWEEFTETFGANKETKLPKMIVWDGDEEYLSVIGSESIEEEDQGSQISQFLAGYRGGRTERNRVSGPSLLGYISSLIGIRTVYIIVFLVAML

MFIQHISKEEPLRVGTRDQAEPATSSKAESSEYRPEDKQD*

>PtPDIL7-2 Potri.013G111400.1

MKTTRSSLILILSIIISATESTRADKINTVLELDESNFDSTIAAYDYVFVDFYAPWCTHCKRLAPELDVAAPILAELKKPIVIAKVNADKYTRLARKHEV

DGYPTLKIYMHGVPTEYYGPRKAELLVRFLRKFVAPDVVVLNSDSAIREFVEEAGTHFPIFIGFGLNETLISNLAIKYKKKAWFSVASDFSDDVMVQYDF

DKIPTVVSIHPSYDDHSIFYGPFEEEFLEEFIEQNFLPLAVPINYDTLKVLKDDQRKIVLTILEDESEEKSQKLIKTLKAAASANRNLVFGYVGVKQWAE

FAETFGAKGTKLPKMIVWDGGEEYLSVIGSESIEEEDQGSQISQFLAGYREGKTERNRISGPSLMGYLNSLIGVRTVYIIVFLVAMLILIRHISKEEPLT

VGTGDQVEHATSSEAESSDYRPGDKQD*

>PtPDIL8-1 Potri.001G419300.1

MVSTNKLKSVDFYRKIPRDLTEASLSGAGLSIVAALAMVFLFGMELNNYLTVNTSTSVIVDNSSDGEFLRIDFNLSFPSLSCEFASVDVSDVLGTNRLNI

TKTIRKFSIDHDLKPTGSEFHSGPVLHHINHGDEVHEEGSEGSVSLKAHNFDQYTHQYPILVVNFYAPWCYWSNRLKPSWEKAAKIIRERYDPEIDGRIL

LAKVDCTEEGDLCRRNHIQGYPSIRIFRKGSDLRDDHGHHDHESYYGDRDTDSLVKTMEGLVAPIAMESQRHALEHKPENATEHVKRPAPSAGGCRIEGY

VRVKKVPGNLVISARSGAHSFDSAQMNLSHVISHFSFGMKVLPRVMSDVKRLIPHIGRSHDKLNGRSFINHRDVGANVTIEHYLQVVKTEVVTRRSSAEH

KLIEEYEYTAHSSLAQTVYMPTAKFHFELSPMQVLITENPKSFSHFITNVCAIIGGVFTVAGILDSILHNTFRMMKKVELGKNF*

>PtPDIL8-2 Potri.011G135500.1

MVSTNKLKSVDFYRKIPRDLTEASLSGAGLSIVAALAMMFLFGMELNNYLTVNTSTTVIVDNSSDGEFLRIDFNISFPSLSCEFASVDVSDVLGTNRLNI

TKTIRKFSIDHDLKPTGSEFHSGPVLHQIKHGDEVDEEGGEGSVSLKAHNFDQYSHQYPILVVNFFAPWCYWSNRLKPSWEKAAKIIRERYDPEMDGRIL

LAKVDCTEEGDLCRRNHIQGYPSIRIFRKGSNLREDHGRHDHESYYGDRDTESLVKTMEALVAPIAMESQRQALEHKPENATQHVKRPAPSAGGCRIEGY

VRVKKVPGNLMISALSGAHSFDSKQMNLSHVISHFSFGMKVLPRVMSDVKRLLPYIGRSHDKLNGRSFINHRDVGANVTIEHYLQVVKTEVVTRRSSSER

KLIEEYEYTAHSSLSQTVYMPTAKFHFELSPMQVLITENSKSFSHFITNVCAIIGGVFTVAGILDSILHHTVRMMKKVELGKNF*

>BrPDIL1-1 Bra016405

MAMRGYALFSILALSLLASSVRSEETATETTKEFVLTLDHTNFTDTVNKHDFIVVEFYAPWCGHCKQLAPEYEKAASELSSHVPPVVLAKIDASEETNRE

FATQYEVQGFPTIKIFRNGGKAVQEYNGPREADGIVTYLKKQSGPASFEIKAAEDASEFDKKVIVVGVFPKLSGSEFDSFLATAEKLRSDYDFAHTSDAK

LLPRGESVTGPVVRLFKPFDELFVDSKDFDGEALEKFVKESSIPLITVFDKDPNNHPYVIKFFDSSNTKAMLFINFTGEGAESLKSKYREVATSYKGQGL

SFLLGDAENSQGAFQYFGLEESQVPLIIIQTVDDKKYLKTNIEIDQIESWVKDFKDGKVAPHKKSQPIPTENNEPVKVVVAESLDEMVFNSGKNVLLEFY

APWCGHCQKLVPILDEVAVSYQSDPSVVIAKLDATANDFPNDTFDVKGFPTIYLRSASGNIVLYDGDRTKEDIISFIDKNKDTAGEPKKEETTTEAVKDEL*

>BrPDIL1-2 Bra012293

MAAMRRGYALFSILALSLLASSVRSETKEFVLTLDHSNFTDTINKHDFIVVEFYAPWCGHCKQLAPEYEKAASELSSNVPAVVLAKIDASEETNKEFATK

YEVQGFPTIKIFRNGGKAVQEYKGPREADGIVSYLKKQSGPASFEIKSGDDVVGDKKVVVVGVFPKLAGSEFDSFLATAEKLRSDYDFAHTSDAKLLPRG

ESVTGPVVRLFKPFDELFVDSKDFDGEALEKFVKESSIPLITVFDKDPNNHPYVIKFFDSPNTKAMFFINFTGESAETLKSKYREVATSNKGQGLSFLLG

DAENSQGAFQYFGLEESQVPLIIIQTADDKKYLKTNVEVDQIGSWIKDFKDGKVSPHKKSQPIPTENNEPVKVVVGESLDDMVFNSGKNVLLEFYAPWCG

HCQKLVPILVEVAVSYQSDPSVVIAKLDATANDFPRDTFDVKGFPTIYFRSASGNVVLYEGDRTKEDFISFIDKNKDTAGEPKTEDKTAEATKDEL*

>BrPDIL1-3 Bra017948

MAMKGYTLCSILVFSLFASCVRSKETKEFVLTLDHTNFTETINKHDFIVVEFYAPWCGHCKQLAPEYEKAASELSSHVPPVVLAKIDASEETNKEFATKY

SVQGFPTIKILRNGGKAVQEYNGPREADGIVTYLKKQSGPASLEIKSADAASEVVGDKNVVAVGVFPKLSGAEFDSFMATAEKLRSDYDFAHTTDAKLLP

RGESVTGPVVRLFKPFDELFVDFRDFVGEALEKFVKESSIPLITVFDSDPNNHPYVLKFFEIPNTKALFFLNFNGEGAETLKSKYREVAASNKGHGLSFL

LGDAKNSEEALQHYGVEQRQLPLIILQTVDDKKYLKTNVEVDQIESWINDFKDGKASPYKKSQPIPGENNEPVKVVVAENLDEMVFSSGKNVLLEFYAPW

CGHCQNLVPILDEVAVSYQSDPSVVIAKFDATANDFPHDTFDVKGFPTIYLRSANGNIVLYKGDRTKEDIISFIDKNKDTAGETKTEEKKTKEVKDEL*

>BrPDIL1-4 Bra015665

MASNGFAMLSILVLALFASSIRSEETETKEFVLTLDHSNFTDTINKHDFIVVEFYAPWCGHCKSLAPEYEKAAAELSSQSPPIFLAKIDASEESNKGIAN

DYKIQGFPTIKILRKGGKSIQDYNGPREAAGIVTYVKKQSGPASAEIKSADGAGEVIGEKSVVAVGVFPKLSGEEFDSFMALAEKLRADYDFAHTLDAKL

LPRGDSSVAGPVVRLFKPFDELFVDSKDFNGEALEKFVKESSIPLVTVFDKDPSNHPYVSKFFDNPATKVMMFVNFTGETAESLKSKFREVATSSKGQDL

AFLVGDAESSQGALQYFGLEESQVPLIIIQTPDSKKYLKANVVVDQIESWMKDFKDGKVAAHKKSQPIPAENNEPVKVVVAESLDEMVFNSGKNVLIEFY

APWCGHCQKLAPILDEVALAFQNDPSVIVAKLDATANDIPSDTFDVKGFPTIYFRSADGKVVVYEGSRTKEDFISFIEKNKPASHSEESSTTVRSGEHKT

EESAAKDEL*

>BrPDIL1-5 Bra008311

MAFKGFALFSIVVLSIFASSRSEETETKEFVLTLDHSNFTETINKHDFIVVEFYAPWCGHCKSLAPEYEKAASELITHNPPLVLAKIDASEESNKGIANE

YKIQGFPTIKILRNGGKSIQDYNGPREAPGIVSYVKKQSGPASSEIKTAADAAEVVGEKNVVAVGVFPKLSGEEFDSFIALAEKLRGDYDFAHTLDAKLL

PRGDSSVAGPVVRLFKPFDELFVDSKDFNGEALEKFLKESSIPLVTVFDSDPSNRPYVASFFDSSATKVMMFVNFTGESAESLKSKFRKVATSYKGQDLS

FLVGDAEGGKGALEYFGVEESQVPLVIIQTPDSKKYLKANVVVEEIESWMKDFKDGKVDVFKKSQPIPAENNEPVKVVVAETLDDIVLKSGKNVLIEFYA

PWCGHCQKIAPILDEVALAFKNDPSVIIAKLDATANDIPSEPFDVKGFPTIYFRSVSGTVVAYEGNRTKEDFISFIEKNKPTTSHVEDTTSSTKTEEPKK

IDDASDTKDEL*

>BrPDIL2-1 Bra020239

MASRVFLLLSLTALLIFSAVSPSLSADVDDEEDLSFLEDLTEEVKAPAKPLTDDFEGGEDDDDEEEDGEHFSDVSNQDSDPFPLSDVDEKDVVVVKERNF

TDVIENNEYVMVEFYAPWRGHCQSLAPEYAAAATELKGDGVVLAKIDATVENELAHQYSVQGFPTILFFVDGEHKLYTGGRTKETIVTWVKKKIGPSVYN

LTTLDDAEKVLTSGNKVVLGYLNSLVGVEHDQLAAASKAEDDVNFYQTVNPDVAKMFHIDPESKRPALVLVKREEEKISHFDGEFVKSGLVSFVSANKLP

LVTVFTPESSQEIFESAIKKQLLLFATENGSEKVLQEFEEAATLFKGKLIFVSVDVDNEDYGKPVAEYFGVSSSNAPKLVAFTGNEDPQKHYFEGEIKSD

KIKIFGEEFLSDKLKPFYKSDPIPEKNDGDVKIVVGDNFDEIVLDESKDVLLEVYAPWCGHCQALEPMYNKLAKHLRSIDSVVIAKMDGTTNEHPKAKAE

GFPTVLFFPAGNKTSSEPITVDADRTVVAFYKFLRKHATIPFKLEKPAASTESPTAAESTPKVETTETKGKLESTTTKSTESDSKDEL*

>BrPDIL2-2 Bra002464

MAFRVFLLLSLTALLIFSAVSPSFSTSDVDDEDLSFLEDPKEEHDPTKPLTSTESELDEFNEGEEEDPEMYEGDDEEEGEDLSDLGNPDSDPFPTPDVDE

KDVVVVKERNFTDVIENNQYVMVEFYAPWCGHCQSLAPEYAAAATELKGDGVVLAKIDATEENELAHQYSVQGFPTILFFVDGEHKPYTGGRTKDTIVTW

VKKKIGPSVYNLTTLDDAEKVLTSGNKVVLGYLNSLVGVEHDQLAAASKAEDDVNFYQTVNPDVAKLFHIDPEAKRPAVVLVKREAEKISHFDGEFVKSD

LASFVSANKLPLVSVFTRESAPEIFESAIKKQILLFVTQNGSEKVLPEFEEAAKSFKGKLIFVSVDLDNEDYGKPVAEYFGVSGNGPKLIAYTGNEDPKK

HFFDGEIKSDKIKTFAEEFLSDKLKPFYKSDPIPEKNDGDVKIVVGDNFDDIVLDESKDVLLEVYAPWCGHCQALEPMYNKLAKHLREIDSLVIAKMDGT

TNEHPKAKAEGFPTILFFPAGNKTAEPITVDTDRTVVAFYKFLRKHATIPFKLEKPAASTESPKTAKSTPKVETTETKGNPQSTTKSTESDLKDEL*

>BrPDIL2-3 Bra007120

MASSTSMSLLFLLSFLLLATSRAENAANGSDLDEELAFLAAEESKEEQQHHANSHHDQYRDFENYEDLEQGGEFHHGEHEGGGEYHEEEPQLPIVDEKDV

AVLTKDNFTEFVGNNSFAMVEFYAPWCGACQALAPEYAAAATELKGVAALAKIDATEEGDLAQKYEIQGFPTVFLFVDGEMRKTYEGERTKDGIVTWMKK

KASPSIHNITTVEEAERVLSAEPKVVLAFLDSLVGSESAELAAASRLEDDLSFYQTTSPDIAKLFEIETEVKRPALVLLKKEEEKLARFDGNFTKAAISE

FVSANKSPLVINFTREGASLIFENSVKNQLILFATTNESEKHLPTLREVAKSFKGKFVFVYVQMDNEDYGEAVSGFFGVTGTAPKVLVYTGNEDMRKFIL

DGELTVNNIKTLAEDFLADKLKPFYKSDPVPETNDGDVKIIVGNNFDEIVLDESKDVLLEIYAPWCGYCQSFEPIYNKLGKYLKGIDSLVVAKMDGTTNE

HPRAKADGFPTILFFPGGNKSFDPITVDVDRTVVELYKFLKKHASVPFKLAKPSATPEQVITTKKADEKTESDGAKDEL*

>BrPDIL3-1 Bra018958

MSINPKPQSSLLTFILLLLLTSAAYSSSNHPGSDEESDDLEQLLAVDEQLQQDLPLHHQQSEAETVSRAQRIVLELSGDNARRVVGGNEFVMVLGYAPWC

ARSADLMPKFSEAATALKEIGSPVVMAKIDGDRYGKVASEMEIKGFPTLLLFVNGTSKAYTGGFSAEEIVIWVQKKTGAPIVTVNTVDEAQRFLKKYHTF

VVGLFNKFEGSEYNEFVKAAKSDDEIQFVETSDSEVAKLLFPEIKTSDVFIGMVKTEAERYTSYAGSYKMENILEFLSKNKFPLITKLSESNTAWVYSSP

VKLQVMIFAKADDFQNMAQPLENFARRFKSKLMFIYIDITNENLAMPFLTLFGIEHANKTVVAAFDNKLNSKYLLESDPSPTNIEDFCSGLADGTIPQYY

RSEPVPDNENASIVTVVGKTFDELVLNSQENVLLEVHTPWCVNCEAMSKQVVKLAKHFKGFENLVFARIDASTNEHAKLQVNDYPTILLYKSGEKEKPLK

ISTKLSAKDMAVFINEELKPRGGSAKDEL*

>BrPDIL3-2 Bra014319

MSMNPKLSVSTFILLLLLTFLLIPSHSSSSDEESDDDLEQLLAVDEQSQEDRPQHQQSEAETVSKAQRIVLELTGDNAKRVVDGNEFVLVLGYAPWCARS

ADLMPRFSEAATGLKEIGSSVLMAKIDGDRYGKVASELEIKGFPTLLLFVNGTSQPYSGGFSAEDIVIWVQKKTGSPIITVNTLDEAQIFLNKYHTFVVG

LFHKFEGSEYNEFVKAAKSDNEIQFVETSDNDVAKLLFPQLKTNTVFIGLVKPEAERYTAYDGPFKMEKLLEFLGNNKFPLITRLTESNTVWVYSSPVKL

QVMLFSKAYVFQSLAQPLEDLARKFKSKLMFIYVDIANENLAMPFLTLFGIEHANKTVDFCSGLADGTVSRYYRSEPVPDNVDDFPTILLYKSGEKEKPV

ITFLIEQSYKPSM*

>BrPDIL4-1 Bra004455

MAKSQIWFGLALVALLVVSAVADDVVVLTEDSFEKEVGKDKGALVEFYAPWCGHCKKLAPEYEKLAASFKKAKSVLIAKVDCDEHKGVCTKYDVSGYPTI

KWFPKGSLEPQKYEGPRNAEALAEFVNKEGGTNVKLAAVPQNVVVLTPDNFDEIVLDQNKDVLVEFYAPWCGHCKSLAPVYEKVATVFKQEDGVVIANLD

ADAHKSLGEKYGVSGFPTLKFFPKDNKAGQDYDGGRDLDDFVTFINEKVGTSRDSKGQLTSKAGVVESLDALVKELVAASEDEKKAILSRIEEEASNLKG

STARYGKLYSSLAKKYIEKGSGYATKEAERLGRVLSKSMSPVKADELTLKRNILNTFVASS*

>BrPDIL4-2 Bra000454

MAKSQIWFGLASLVALLVVSAVADDVVVLTDDSFEKEVGKDRGALVEFYAPWCGHCKKLAPEYEKLGASFKKAKSILIAKVDCDEHKSVCTKYGVSGYPT

IQWFPKGSLEPQKYEGARNAEALAEYVNKEGGTNVKLAAAPQNVVVLTPDNFDEIVLDQNKDVLVEFYAPWCGHCKSLAPVYEKVATVFKQEEGVVIANL

DADAHKSLGEKYGVSGFPTLKFFPKDNKAGQDYEGGRDLDDFVGFINEKVGTSRDSQGQLTSKAGIVESLDALVKELVAASEDEKKTILSRIEEEASNLK

GSTTRYGKLYSKLAKSYIEKGSAYATKEVERLGRVLGKSISPVKADELTLKKNILSTFVASS*

>BrPDIL5-1 Bra005546

MQNKSPLTLLTLLCLSLGFLNLTNALYGSSSPVVQLTASNFKSKVLNSNGVVLVEFFAPWCGHCKALTPTWEKVASVLKGVATVAAIDADAHQSAAQDYG

IQGFPTIKVFVPGKPPVDYQGARDAKSIANFAYKQIKALLSDRLEGKSKPSGGGSSEKKSEPSASVELNSSNFDELVIKSNDLWIVEFFAPWCGHCKKLA

PEWKRAAKNLKGKVKLGHVNCDVEQSIMSRFKVQGFPTIMVFGVDKSSPYAYDGARSASAIESFATELVEASAGPVEVTELTGPDVMEKKCGSAAICFVS

FLPDILDSKAEGRNKYLEMLLSVAEKFKRHPYSFVWVAAVTQPDLEKRVNVGGYGYPAMVAMNVKKGVYAPLKSAFELQHLLEFVKDAGAGGKGNVPMNG

TPEIVETKAWDGKDGEVMEEDEFSLEELMGGDDDANVGTKDEL*

>BrPDIL5-2 Bra015375

MNKTRVFTILSLVFAFSFDLSNALYGSSSPVLQLTPSNFKSKVINSNGVVLVEFFAPWCGHCKSLTPTWEKVATTLKGIATVAAIDADAHKSVSQDYGVR

GFPTIKVFVPGKPPIDYQGARDAKAISQFAIKQIKALLKDRLDGKTTGTTTGGGSSEKKSEPSASVELNSSNFDELVTESKDLWIVEFFAPWCGHCKKLA

PEWKKAAKNLKGKVKLGHVDCDADKAIQSRFKVKGFPTILVFGADKSSPLPYEGARSASAIESFALEQLEANAGPAEVTELTGPDAMEEKCGPAAICFVS

FLPDILDSKAEGRNKYLEMLLSVAEKFKKDPISFVWVAAGKQPDLEKRVGVGGYGYPAMVALNAKKGAYAPLKSGFEVKHLIEFVKEAQKGGKGNLPIDG

TLEIVKTEAWDGKDGEVVDADEFSLEELMADD*

>BrPDIL6-1 Bra018672

MKLGARLIAFILLLSLTIVLTKAEVITLTPETFSDKVKEKDTAWFVKFCVPWCKHCKKLGNLWEELGNAMEGDDEIEIGEVDCGKSRDVCTKVEIHSYPT

FKLFYNGEEVSKYQGKRDVESLKTFVVEETEKAAEKAQLEDKEL*

>BrPDIL7-1 Bra034408

MRSLGLMYWWISFLALSISLSASSDDQFTIDGTVLELTDSNFESAISTFDCVFVDFYAPWCGHCKRLNPELDAAAPILAKLKQPIIIAKLNADKYSRLAR

KLEIDAFPTLMLYNHGVPMEYYGPRKADLLVRYLKKFVAPDVAVLESNSHVKDFVEDSGTSFPVFIGFGLNQSLISGLGRKYKKKAWFAVAKDASEDVMV

SYDFDKAPALVAQHPAYNEHSVFYGPFEDGFLEEFVKQNFLPLILPINHDTLKLLKDDERKMVLTIVEDETHESMGKLIKALRAAAHANRDLVFGYVGVE

QFEEFADSFHADKKAKLPKIVVWDGDEEYEQVNGIETVSHEEDHLTQVSRFLEGYREGKTEKKRIKGPSFMGFINSMIGIRSVYIIVFLVAVIMMLRSLG

QVEEPARVRTAASDGQATSVLEGETSEHKPRDKED*

>BrPDIL8-1 Bra001793

MVSPTKLKSVDFYRKIPRDLTEASLSGAGLSIVAALVMMLLFGMELSSYLEVTTTTAVVVDKSSDGDFLRIDFNISFPALSCEFASLDVNDVLGTNRLNI

TKTVRKFPIDPHLKATGGEFHSGLASHHINHGEEIKQEFPDGAIQLTNGGFQSLSHHFPLLIVNFNAPWCYWSNRLKPSWEKAATIIKQRYNPDTDGRVL

LGSVDCTEEPALCRRNHIQGYPSIRIFRKGNDLKEDHGHHEHESYYGDRDTESIVKMVDELVAPIHPETHKLALDWGISNDTAKLLKKAPVTGGCRVEGY

VRVKKVPGNLVISAHSGAHSFDSSQMNMSHVVTHLSFGRMIDTRLLTDLKRLLPYLGQSHDKLDEKAFINQHEFGANVTIEHYLQIVKTEVITRRYGQEH

SLTEEHEYTAHSSITQTYYLPVAKFHFELSPMQILITENPKSFSHFITNLCAIIGGVFTVAGIIDSVLHNTIRLIKKVELGKNI*

>BrPDIL8-2 Bra035770

MVSPTKLKSMDFYRKIPRDLTEASLSGAGLSIVAALVMMLLFGMELSSYLAVNTTTAVVVDKSADGDFLRIHFNISFPALSCEFASVDVSDVLGTNRLNI

TKTIRKFPIDPHLKTTGEEFHSGHGSHDINHGEETKEEIPDGSVPLVSSSFDSFSKHFPLLIVNFNAPWCYWSNRLKPSWEKASSIIYHKYNPETDGRVL

LGSVDCTEEAELCKRNHIQGYPSIRIFRKGSDLKEDHGHHEHESYYGDRDTDSIVKMVDELVAPIHPETHKLDLDGISNKTLKHLKKAPVTGGCRVEGYV

RVKKVPGNLIISAHSGAHSFDSSKMNMSHVVSHLSFGRMFSPRLLTDMRRLLPYIGQSHDKLNEKAFINQHEFGANVTIEHYLQVVKTEVITRRTAQEHS

LVEEYEYTAHSSIAQTYYLPVAKFHFELSPMQIMITENPKSFSHFITNLCAIIGGVFTVAGILDSIFHNTIRLVKKVELGKNF*

>BrPDIL8-3 Bra030465

MVSTTKIKSVDFYRKIPRDLTEASLSGAGLSIIAALAMVFLFGMELSTYLAVTTNTSVIVDNSSDGDFLRIDFNVSFPSLSCEFASVDVSNVLGTKRLNL

TKTIKKVPIDPYLRATGAEVHSTSGLHLINHGDEDHGNNTYAAIPLTGATFDKFSHHFQILVVNFYAPWCYWSNRLKPSWEKAAEITRQRYNPETDGRVL

LGSVDCTEETTLCKRNHIQGYPSIRIFRKGSDLKEDHGHHEHESYHGDRDTESILKMVEELLKPIKKEDHKLALDGKTDNVVSGIKKAPVSGGCRIVGYV

RAKKVPGEIIISAHSGAHSFDASQMNMSHYVSHLTFGKMISERLLTDMKRLMPYLGLSHDRLNSKWFVNEGQFAANVTIEHYLQVVKTEVVSRRFGQEHS

VIEEYEYTAHSSVAHGYYYPVAKFRFDLSPMQVLISENPKSFSHFITNVCAIIGGVFTVAGILDSIFQNTFRLVKKIELGKNI*

>BrPDIL8-4 Bra010413

MISPRKIKSVDFYRKIPRDLTEASLSGAGLSIVAALSMLLLFGMELSSYLTVSTTTSIIIDRSSDGDFLRMDFNISFPSVSCEFASVDVSDVLGTNRLNV

TKTIRKFSIDSNLRPTGSEFHSGEFLSRVNHGDESAEELVEGSVSLGARNFDTFLHQYPISVVNFYAPWCYWCNLLKPSWEKAANQIKERYDPEMDGRVI

LAKVDCTQEADLCRRNHIQGYPSIRIFRKGSDLRDDNAHHDHESYYGDRDTESLVKMVIGLVEPIHLEPHKLALEDKSGNASKTLKKAPSTGGCRIEGYM

RVKKVPGNLMVSARSESHSFDTSQMNMSHVVNHLSFGKRILPEAFSDLKRLAPYLGGSHNRLDDRSFINQHDLGPNVTIEHYLQIVKTEVLKSNGHAMIE

EYEYTAHSSVAHTYYLPVAKFHFELSAMQVLITENSKSFSHFITNVCAIIGGVFTVAGILDSILHQTMTLMKKIELGKNF*

>BrPDIL8-5 Bra019071

MVSTSRIKSVDFYRKIPRDLTEATLSGAGLSIVAALSMLFLFGMELNNYLAVSTTTSIIVDRSSDGDFLRMDFNISFPSLSCEFASVDVSDVLGTNRLNV

TKTIRKFSIDSNLRPTGSEFHSGEVLSHVNHDEAGEEVVEDSVSLTSRNFDTLLHQFPISVVNFYAPWCYWCNLLKPSWEKAAKQIKERYDPEMDGRVIL

AKVDCTQEADLCRKNHIQGYPSIRIFRQGSDLKDNAHHDHESYYGDRDTESLVKMVIGLVEPIHLEPHKLALEDKSDNASKTLKKAPSTGGCRIEGYIRV

KKVPGNLMVSARSGSHSFDSTQMNMSHVVNHLSFGRKILPQTFTDLKRLSPYLGQSHDRLNGRPFINQRDLGPNVTIEHYLQIVKTEVLKSNGHAMVEEY

EYTAHSSVAHSYYLPVAKFHFELSPMQVLITENSRSFSHFITNVCAIIGGVFTVAGILDSILHQTMTLMKKIELGKNF*

>BrPDIL8-6 Bra018881

MVSTTKIKSVDFYRKIPRDLTEASLSGAGLSIIAALAMMFLFGMELSTYLAVTTQTSVVVDNSSDDDFLQIDFNVSFPALSCEFATFEVSDVLSTNRLNL

TKTIKKVPIDPHLRDTGEEYHPTPDSDLINHGDEHHDDNTYAAIPLSGGTFDKISHKFPILVVNFYAPWCYWSSRLRPSWEKAAEITRQKYGPENDGRVL

LGSVDCTEEPTLCTKYHIQGYPSIRIFHNGSDLRGDDGHQEHDSYHGNRDTESLVKMVEELLRPIKKFDGTTNHAASRIRKAPVSGGCRIEGYVRAKKVP

GELVISAVSGSHSFDASRMNMTHFVNHLSFGRLISDRLLTDMKRLLPYLGLSHDRLNGKWFVNEGKFAANVTIEHYLQVVKTEVVSRRFGQEHSVIEEYE

YTAHSSVAHGYYYPVAKFHFDLSPMQVLISENPKSFSHFITNVCAIIGGVFTVAGILDSIFQSTYGIMKKVELGKNF*

>BdPDIL1-1 Bradi4g23180.1

MAICNKAWISLLLALAVVLAAPAARAEEAAAAEEAAPAAGEEAVLTLGTDNFDDAIAKHPFIVVEFYAPWCGHCKSLAPEYEKAAQLLSKHDPPIVLAKV

DANDEKNKPLAAKYEIQGFPTLKIFRNQGKNIQEYKGPREAEGIVDYLKKQVGPASKEIKAPEDASHLEDGKIHIVGVFAELSGPEFTNFLEVAEKLRSD

YDFGHTVHANHLPRGETAVERPLVRLFKPFDELVVDTKNFEVSALEAFIEASSTPKVVTFDKNPDNHPYLLKFFQGNSAKVMLFLNFSTGPYESFKSAYY

GAVEDFKDKEVKYLIGDIEASQGALQYFGLNADQAPLILIQDAESKKFLNSNIEADQIVSWLKEYFDGKLTPFRKSEPIPEANNEPVKVVVADNLDDVVF

KSGKNVLIEFYAPWCGHCKKLAPILDEAATTLQSEADVVIAKMDATANDVPGDFDVQGYPTLYFVTPSGKKVAYDGGRTADDIVEYIKKNKETAGQEAAA

ATEKAADPAATESLKDEL

>BdPDIL1-2 Bradi5g10610.3

MAVPLALPSAAIIIVVLLLSSGLTTAEVEVAAVLEEAVLTLDVSNFSEVVGKLQFIVVEFYAPWCGHCKELAPEYEKAASMLRKHDPPVVLAKVDAYDEG

NKELKDKYEVHGYPAIKIIRNGGSDVSGYAGARNADGIVEYLKKQVGPASIELRSALDATRSIGDKGVVLVGIFPEFAGVEYENFMAVADKMRSDYDFFH

TSDASILPHGDQNVKGPLVRLFKPFDELFVDSQDFDKDAIKKFIEVSGFPTVVTFDDEPTNHKFLERYYSTPSAKAMLFLRFSDDRVEAFKSQMHEAARQ

LSGNNISFLIGDVSAAERAFQYFGLKESDIPLLLVIASTGKYLNPTMDPDQLIPWMKQYIYGNLTPYVKSEPIPKVNDQPVKVVVADNIDDIVFNSGKNV

LLEFYAPWCGHCRKLAPILEEVAVSFRNDEDIVIAKMDGTANDVPTDFVVEGYPALYFYSSSGGEILSYKGARTAEEIISFIKKNRGPKAGALEEVTQTD

AVQEEVTSTSSPSESVKDEL

>BdPDIL2-1 Bradi3g00210.1

MVSPRSLLLLLLLASPFLLLLLHASSDEDLDYIIHNAAADDLPADDEWLQEGSDDDQEESDPFHQGDIDETHVFLLTAANFSDFLSSRRHVMVEFYAPWC

GHCQALAPDYAAAASQLALLHQDVVALSKVDATEDADLAQKYDVQGFPTILFFIDGVPKDYTGERTKEAIVAWINKKLGPGVHNVTTVDEAEKIITGEDK

AVLAFLDSLSGAHSNELAAASRLEDTINFYQTSNPDVAKLFHIDPAAKRPSVVLLKKEEEKLTIYEGEFRASAIADFVSANKLPLITILTQETGPSIFDN

PIKKQILLFAVANESSEFLPIFKEVAKPFKGKLLFVFVERDNEEVGEPVANYFGITGQETTVLAYTGNEDAKKFFLDGEMSLDNIKKFAQDFLEDKLTPF

YKSEPIPEPNDEDVKIIVGKNLDQIVLDESKDVLLEIYAPWCGHCQSLEPTYNKLAKHLRGIDSLVIAKMDGTTNEHPRAKPDGFPTILFYPAGKKSFEP

MTFEGDRTVVEMYKFIKKHASIPFKLKRPDSSAARTERAESSGSTEGEKSSGSNLKDEL

>BdPDIL3-1 Bradi1g48460.1

MRAWRRLAVVALLAALLAVSSAAARLDLGEDDDSEVLEALLAVDEEEEDEAPEGAKRAGGAEAVRRTQSMVLVLDNENARRAVEEHAELLLLGYAPWCER

SAQLMPRFAEAAAALRAMGSAVAFAKLDGERYPKAAADVGVSGFPTVLLFVNGTEHAYTGLHTKDALVTWVRKKTGAPVIRLQSRDSAEEFLKKDQTFAI

GLFKNYEGADHEEFVKAATTENEVQFVETNDRNVAKILFPGIASEEQFLGLVKSEPEKFEKFDGAFEENAILQFVELNKFPLITVFTDLNSGKVYGSPIK

LQVFTFAEAYDFEDLESLLQEVARGFKTKIMFIYVDTAEEKLAKPFLTLYGLEGDKPTVTAFDTSKGSKYLMEADINAKNLKEFCSGLLDGTLPPYFRSE

PVPQEKGLIGKVVGRTFDSSVLESPHNVFLEAHAPWCVDCEAISKNVEKLAKHFSGLDNLKFARIDASVNEHPKLQVNDYPTLLLYPAEDKSNPIKVSKK

LSLKDMAKFIKVKLHISDVDIKEKEPASDVEAVAATDSVKDEL

>BdPDIL4-1 Bradi2g12560.1

MAIRQISRQTLALLFVVAAASFAALVFADGDDVVVLTEGTFEKEVGQDRGALVEFYAPWCGHCKKLAPEYEKLGASFKKARSVMIAKVDCDEHKSVCSKY

GVSGYPTIQWFPKGSLEPKKYEGQRTAEALAEFVNKEGGTNVKLATIPSSVVVLTPETFDSVVLDETKDVLVEFYAPWCGHCKHLAPIYEKLASAFKLDD

GVVIANVDADKYKDLGEKYGVTGFPTLKFFPKGNKAGEDYDGGRDLGDFTKFINEKCGTSRDTNGQLTSEAGRIASLDTLAKEFLSVASDKRKEVLSSIE

EEVAKLSGSAAKHGKVYVTIAKKILDKGNDYTKKETERLHRILEKSISPSKADEFIIKKNVLSTFSS

>BdPDIL4-2 Bradi2g35020.1

MATPQISRKTLALVLLLVAAAAAALSPAAAEGDEVLALTESTFDKEVGQDRAALVEFYAPWCGHCKKLAPEYEKLAASFKKAKSVLIAKVDCDEHKSVCS

KYGVSGYPTIQWFPKGSLEPKKYEGQRTAEALAEYVNSEAATNVKIAAVPSSVVVLTEETFDSVVLDETKDVLVEFYAPWCGHCKSLAPVYEKVASAFKL

EDGVVIANLDADKHTSLAEKYGVSGFPTLKFFPKGNKAGEEYEGGRDLEDFVKFINEKSGTSRDSKGQLTSEAGLVASLDALVKEFHSAADDKRKEVLSK

IEEEAAKLSGSAAKHGKIYVNAAKKIIEKGSDYTKKETERLHRMLEKSISPSKADEFVIKKNILAIFSS

>BdPDIL5-1 Bradi4g31830.1

MHPAFLAALLLLFAAAASPAAALYSAGSPVLQLNPNNFKKVLNANGVVLVEFFAPWCGHCKQLTPTWEKAAGVLKGVATIAALDADAHKELAQQYGIQGF

PTIKVFIPGKPPVDYEGARDVKPIVNFALQQVKSLLKDRLDGKTSGGSSGKTSGGSSEKKTDTNESIELNSSNFDELVIKSKDLWIVEFFAPWCGHCKKL

APEWKRAAKNLKGQVKLGHVDCDSDKSLMSKYKVEGFPTILVFGADKESPFPYQGARAASAIESFALEQLEANSAPPEVSELTSSDVMEEKCASAAICFV

SFLPDILDSKAEGRNKYLELLLSVAEKFKKSPYSFVWTGAGKQADLEKQVGVGGYGYPAMVALNVKKGAYAPLRSAFQRDEIIEFVKEAGRGGKGNLPLD

GAPTVVQSGPWDGKDGEVIEEDEFSLEELMGDNSPPNDEL

>BdPDIL6-1 Bradi1g65710.1

MDPALRRRSRLPIHMLVLVVAVLVVLAARSGAEVITLTEETFTDKVKEKDTVWFVQFCVPWCKHCKSLGTLWEDLGKVIEGTDEIEIGKVDCGASKPVCS

KVDIHSYPTFKVFYDGEEVAKYKGPRNVESLKNFVLNEAEKAGEARLQDEL

>BdPDIL7-1 Bradi5g10380.1

MATTLPPLPLLCLLPLLLVAFATAGGGSGGGGGENFPRDGRVIDLDDSNFEAALSSIDFLFVDFYAPWCGHCKRLAPELDEAAPVLAGLSEPIMVAKVNA

DKYRKLGSKYGVDGFPTLMLFIHGVPIEYTGSRKADLLVRNLKKFVAPDVSILESDSAIKSFVENAGTSFPMFIGFGVNESLITEYGGKYKKRAWFAIAQ

DFSEELMMAYGFDKAPALVALHPKYNEQSVFYGPFEGRFLEDFIRQSLLPLTVPINTETLKLLDDDDRKVVLAILEDDSDENSAQLVTVLRSAANANRDL

VFGYVGVKQWEEFVETFDVSKSSQLPKLLVWDRNEEYEQVDGSERLEEGDQASQISQFLEGYRAGRTTKKKVSGPSFMGFMHSLVSMNSLYILMFVVALL

GVMLYFSGQDDTPQLRRIHDE

>BdPDIL7-2 Bradi3g45540.1

MAAGKPLSLRRLLPLLALVLVLVLLPTTCVSSGGGEPAGFQIPQDGSVVELDDSNFEAAAAAVDFLFVDFHAPWCGHCKRLSPQLDEAAPVLAGLSTPVV

VAKVDAEKYKKLGSKYGVDGFPTLMLFDHGVPTEYTGSRKADLLIQSLKKLVAPDFSVLGSDSAIKSFVQDAGVGFPLFIGFGVDESSIVEYGTRYKRKA

WFAAAKDFSEDMMVVYDFDKIPALVSLNPKYNEQSVFYGPFEGTFLEDFIRQSLLPLTVPINAETVKMLKDDERKVVLTVLEDESDENSMQLIKVLRSAA

NANHDLVFGYVGVKQWEEFTEPFHDSESSRLPRMVVWDRNEEYEVVQGSENLEDGDHGSQISRFLEGYRAGRTTKKKLGGRSPTILGVNAMYILLFLVAV

LVVLMYFSGQGEEDRWPARAHQE

>BdPDIL8-1 Bradi1g25977.3

MISSSKLKSVDFYRKIPRDLTEASLSGAGLSIVAALAMVFLFGMELSSYLAVNTTTSVIVDRSSDGEFLRIDFNMSFPALSCEFASVDVSDVLGTNRLNI

TKTVRKFSIDRNLVPTGSEFHSGPIPTVNKHGDDVEEYHADGSVALSSRNFDSYSHQYPILVVNFYAPWCYWSNRLKPSWEKTAKIIKERYDPEMDGRIL

LAKVDCTEEGELCKRHHIQGYPSIRIFRKGSDMKENQGHHDHESYYGERDTDSLVAAMETYVGNLPKEAHMLALDDKSNKTVDPAKRPAPMTSGCRVEGF

VRVKKVPGSVIISARSGSHSFDPSQINVSHYVTQFSFGNRLSPNMFSELKRLIPYVGGHHDRLAGQSYIVKHGDNNANVTIEHYLQIVKTELVTLRSSKE

LKVFEEYEYTAHSSLVHSFYVPVVKFHFEPSPMQVLVTELPKSFSHFITNVCAIIGGVFTVAGILDSILHNTLRLVKKVELGKDI

>ZmPDIL1-1 GRMZM2G091481_T01

MAIRSKAWISLLLALAVALSARAEEEPAAAAEGEAVLTLDVDSFDEAVAKHPFMVVEFYAPWCGHCKKLAPEYENAAKALSKHDPPIVLAKVDANEEKNR

PLATKYEIQGFPTIKIFRDQGKNIQEYKGPREADGIVDYLKKQVGPASKEIKSPEDATALIDDKKIYIVGIFAEFSGTEFTNFMEVAEKLRSDYDFGHTL

HANHLPRGDAAVERPLVRLLKPFDELVVDSKDFDVAALMKFIDASTIPRVVTFDKNPDNHPYLMKFFQSSAPKAMLFLNFSTGPFDSFKSAYSAAAEEFK

DKEIKFLIGDIEASQGAFQYFGLKEDQTPLILIQDGDSKKFLKVHVEADQIVAWLKEYFDGKLTPFRKSEPIPEVNNEPVKVVVADNVHDFVFKSGKNVL

IEFYAPWCGHCKKLAPILDEAATTLQSDEEVVIAKMDATANDVPSEFDVQGYPTLYFVTPSGKVTSYDSGRTADDIVDFIKKSKETAGAATTTTTQAPPA

SEKAAAAEPVKDEL*

>ZmPDIL1-2 GRMZM2G163421_T01

MAIRSKAWISLLLALAAVLSARAEEPAAAEAEAVLTLDVDSFDEAVAKHPFMVVEFYAPWCGHCKNLAPEYENAAKELSKHDPPIVLAKVDANEEKNRPL

ATKYEIQGFPTLKIFRNQGKNIQEYKGPREADGIVDYLKKQVGPASKEIKSAEGVAAHFDDKKIYIVGIFKEFSGTEFTNFMELAEKLSSDYDFGHTLHA

NHLPRGDASVEGPLIRLLKPFDDLVVDSKDFDVAALEKFIDASSTPRVVTFDNNPDNHPYLMKFFQSSAPKAMLFLNFSTGPLDSFKSVYYAAAEEFKDK

EIKFLIGDIEASQGAFQYFGLKEDQTPLILIQDGDSKKFLKDHIEADQIVSWLKEYFDGKLTPFKKSEPIPEVNNEPVKVVVADNIHDVVFKSGKNVLIE

FYAPWCGHCKKLAPILEEAATTLLSDEEVVIAKMDATANDVPSEFEVQGYPTMYFVTPSGKVTSYDSGRTADDIVDFINKSKETASAVQATATASGKAAD

AAEKTEPVKDEL*

>ZmPDIL2-1 GRMZM2G134889_T01

MGSTTMSPPSFPVVLLLLLLATIAAAAGSNMDEEVVDDLQYLIDNSDDIPTNDPDGWPEGDYDDDDLLFQDQDQDLTGHQPEIDETHVVVLAAANFSSFL

ASSHHVMVEFYAPWCGHCQELAPDYAAAAAHLAAHHHQAHLALAKVDATEETDLAQKYDVQGFPTILFFIDGVPRGYNGARTKEAIVDWINKKLGPAVQN

VTSVDEAQSILTGDDKAVLAFLDTLSGAHSDELAAASRLEDSINFYQTSTPDVAKLFHIDAAAKRPSVVLLKKEEEKLTFYDGEFKASAIAGFVSANKLP

LVTTLTQETSPSIFGNPIKKQILLFAVASESTKFLPIFKEAAKPFKGKLLFVFVERDSEEVGEPVADYFGITGQETTVLAYTGNEDARKFFLDGEVSLEA

IKDFAEGFLEDKLTPFYKSEPVPESNDGDVKIVVGKNLDLIVFDETKDVLLEIYAPWCGHCQSLEPTYNNLAKHLRSVDSLVVAKMDGTTNEHPRAKSDG

YPTILFYPAGKKSFEPITFEGERTVVDLYKFIKKHASIPFKLKRQESRTESTRAEGVKSSGTNSKDEL*

>ZmPDIL2-2 GRMZM2G033829_T01

MGSTRTSHPSFPVLLLFLLATAAAAGSNKAEEVDDLQYLIDNSEDIPPNDPDGWPEGGGGGDYDDDLLFQDQDQDLPDYEPQIDETHVVVLTAANFSSFL

AATRHVMVEFYAPWCGHCRELAPEYAAAAAHLAVHHNQTDLALAKADATEETDLAQRYDVQGFPTIILFIDGVPKDYNGARTKDAIVDWINKKLGPAVQD

VTSVHEAERILTGDDKAVIAFLDTLTGAHSDELAAASRLEDSINFYQTSIPDVAKLFHIDPAAKRPSIVLLKKEEEKLTFYDGKFKASAIADFVSANKLP

LVTTLTQETSPSIFGNAIKKQILLFAVASESSKFLSIFKEAAKPFKGKLLFVFVERDNDEVGEPVANYFGLTGQETTVLAYTGNEDARKFFLDGEVSLEA

IKDFAEGFLEDKLTPFYKSEPVPESNDGDVKIVVGKSLDVIVLDESKDVLLEIYAPWCGHCQSLEPTYNKLAKHLSGVDSLVIAKMDGTTNEHPRAKSDG

YPTILFYPAGKKSFEPVTFEGERTVVDMYRFIKKHASIPFKLKRQESRRESIQTDGVKDEL*

>ZmPDIL3-1 GRMZM2G014076_T01

MRARWAVTLLLLAVLALTASAARLDLDDDDDSGVLDELLAIDEEAERGGLLDAEGAGEAVRRAQSMVLALDNDNARRAVEDHAELLLLGYAPWCERSAQL

MPRFAEAAAALRAMGSAVAFAKLDGERYPKAAAAVGVKGFPTVLLFVNGTEHAYHGLHTKDAIVTWVRKKTGVPIIRLQSKDSAEEFLKKDMTFVIGLFK

NFEGADHEEFVKAATTDNEVQFVETSDTSVAKVLFPGITSEEKFVGLVKSEPEKFEKFDGKFEEKEILRFVELNKFPLITVFTELNSGKVYSSPIELQVF

TFAEAYDFEDLESMVEEIARAFKTKIMFIYVDTAEENLAKPFLTLYGLESEKKPTVTAFDTSNGAKYLMEADINANNLREFCLSLLDGTLPPYHKSEPLP

QEKGLIEKVVGRTFDSSVLESHQNVFLEVHTPWCVDCEAISKNVEKLAKHFSGSDNLKFARIDASVNEHPKLKVNNYPTLFLYLAEDKSNPIKLSKKSSV

KDMAKLIKEKLQIPDVETVAAPDNVKDEL*

>ZmPDIL4-1 GRMZM2G128171_T03

MAISQISRIFLAILLLAAAFAAAPAALADGDDVVALTESTFEKEVGKDRGALVEFYAPWCGHCKKLAPEYERLGASFKKAKSVLIAKVDCDEHKSLCSKY

GVSGYPTIQWFPKGSLEPKKYEGQRTAEALAEFLNTEGGTNVKLATIPSSVVVLTPETFDSIVLDETKDVLVEFYAPWCGHCKSLAPTYEKVASVFKLDE

GVVIANLDADKHRDLAEKYGVSGFPTLKFFPKGNKAGEDYDGDRDLVDFVKFINEKSGTSRDTKGQLTSEAGRIASLDVLAKEFLGASGDKRKEVLSSME

EEADKLSGSAARHGKVYVTIAKKILEKGNEYTEKETKRLDRILEKVGNAYLARCLMKHPLLGQLTVQI*

>ZmPDIL4-2 GRMZM2G159369_T01

MAFPQISRRALGLLLVIAAAAAIVSPATADEVVALTEADFEKEVGQDRGALVEFYAPWCGHCKKLAPEYEKLGASFKKAKSVLIAKVDCDEHKSVCSKYG

VSGYPTIQWFPKGSLEPKKYEGQRSVEALAEFVNSEAGTNVKIAAIPSSVVVLTSETFDSIVLDETKDVLVEFYAPWCGHCKHLAPIYEKLASVFKQDDG

VVIANIDADKHTDLAEKYGVSGFPTLKFFPKGNKAGEDYDGGRDLDDFVKFINEKCGTSRDPKGHLNQEAGLVPSLNPLVKEFLNAADDKRKEVLSKIEE

DVAKLSGSAAKHGKIYVTAAKKIIDKGSDYTKKETERLHRMLEKSISPSKADEFIVKKNILSIFSS*

>ZmPDIL5-1 GRMZM2G389173_T01

MRPAVVTVLLLVAAAASPAAALYSAGSPVLQLNPNNFKSKVLNSNGVVLVEFFAPWCGHCKQLAPAWEKAAGVLKGVATVAALDADAHQALAQEYGIKGF

PTIKVFSPGKPPVDYQGARDVKPIVEFALSQVKSLLRDRLSGKASAGSNGKTSGGSSEKSEPSASVELNSRNFDELVVKSKDLWIVEFFAPWCGHCKKLA

PEWKKAAKNLKGQVKLGHVDCDAEKSLMSKYKVEGFPTILVFGADKESPFPYQGARVASAIESFALEQLEANSGPAEVSELTGPDVMEEKCASAAICFVS

FLPDILDSKAEGRNKYLELLLSVAEKFKKSPYSFVWTAAGKQANLENQVGVGGYGYPAMVALNVKKGAYAPLRSAFQRDEIIEFVKEAGRGGKGNLPLND

APTVVASEPWDGKDGEVIEEDEFSLDELMGDSSSANDEL*

>ZmPDIL6-1 GRMZM2G073628_T01

MDLGAPARRRLPIRLLLVSLTVLVVLTARSSAEVITLTEETFSDKIKEKDTVWFVQFCVPWCKHCKNLGTLWEDLGKVMEGADEIEIGQVDCGVSKPVCS

KVDIHSYPTFKVFYEGEEVVKYKASMDYTRTNCSVVGTLRTRTSPAATRSRTKCRSISTCFVR*

>ZmPDIL7-1 GRMZM2G176443_T02

MAARVLPPPPLPLVLLLLLLPLSARDTVAAGEDFPRDGRVIDLDESNFEAALGVIDFLFVDFYAPWCGHCKRLAPELDEAAPMLAGLSEPIVVAKVNADK

YRKLGSKYGVDGFPTLMLFIHGVPIEYTGSRKADQLVRNLKKFVAPDVSILESDSAIKNFVENAGTSFPMFLGFGVNDSLIAEYGRKYKKRAWFAVAKDF

SEDVMVAYEFDKVPALVAIHPKYKEQSLFYGPFEENFLEDFVRQSLLPLVVPINTETIKMLNDDQRKVVLTILEDDSDENSTQLVKILRSAASANRDLVF

GYVGIKQWDEFVETFDVSKSSQLPKLLVWDRNEEYELVDGSERLEEGTDQASQISQFLEGYRAGRTTKKKISGPSFMGFLNSLVSLTSLYILIFVIALLF

VMVYFAGQDDTPQPRRIHEE*

>ZmPDIL7-2 GRMZM2G007385_T01

MAMALRRLLLPLLLLVLLGLRPQSCVASGGGGGEPAEFEIPRDGSVLELDESNFEAAVRAAEFLFVDFYAPWCGHCKRLAPQLDEAAAVLAGLSTPVLVA

KVNADKYKKLGSKYGVDGFPTLMFFDHGVPSEYTGSRKADVLVENLKKLVAPDVSVLESDSSINGFVQAAGINFPLFIGFGMDESLIVEYGAKYKKKAWF

STAKDFSEDVMVVYDFDKVPALVSVNPKYNEQSVFYGPFEGTFLEDFIRQSLLPATVPINRETVKLLKDDGRKVVLTILEDESDESSLQLIKVLRSAANA

NHDLVFGYVGVKQWEEFTETFDVKVSQLPKIVVWDTKEEYEVVEGSESFIEGDYGSQVSRFLEGYREGRTTKKKVGRGSPTLLGLNAVYILVLLVAVLVV

LMYFSAQGEEDHQPRRAHED*

>ZmPDIL8-1 GRMZM2G067063_T01

MISSSKLKSVDFYRKIPRDLTEASLSGAGLSIVAALAMVFLFGMELSSYLAVNTTTSVIVDRSSDGEFLRIDFNMSFPALSCEFASVDVSDVLGTNRLNI

TKTVRKYSIDRNLVPTGSEFHPGPIPILNKHGDDVEEDHVDGAFSLSSRNFDSFSHQYPVLVVNFYAPWCYWSNRLKPSWEKTAKIMRERYDPEMDGRIL

LGKVDCTEEVELCRRNHIQGYPSIRVFRKGSDIKENQGHHDHESYYGERDTESLVAAMETYVANIPKEAHALEDKSNKTVDPAKRPAPMASGCRIEGFVR

VKRVPGSVVISARSGSHSFDPSQINVSHYVTQFSFGKRLSPRMLHEFIRLTPYLRGYHDRLAGQSYTVKHGEVNANVTIEHYLQVVKTELVTQRSSKELK

VLEEYEYTAHSSLVHSFYVPVVKFHFEPSPMQVLVTEVPKSFSHFITNVCAIIGGVFTVAGILDSIFHNTLRMVKKIELGKNI*

>AePDIL1-1

MAICKAWISLLLALAVVLSAPAARAEEAAAAEEAAAAPEAVLTLHADNFDDAIAKHPFILVEFYAPWCGHCKSLAPEYEKAAQLLSKLDPAIVLAKVDANDEKNKPLASKYEVQGFPTLKIFRNGGKNIQEYKGPREAEGIVEYLKKQVGPASKEIKAPEDATYLEDGKIHIVGVFTEFSGTEFTNFLEVAEKLRSDYDFGHTVHANHLPRGDAAVERPLVRLFKPFDELVVDSKDFDVSALEKFIDASSTPKVVTFDKNPDNHPYLLKFFQTNAPKAMLFLNFSTGPFESFKSAYYGAVEEFSGKDVKFLIGDIEASQGAFQYFGLKEDQAPLILIQDSDSKKFLKEQVEAGQIVAWLKDYFDGKLTPFRKSEPIPEANNEPVKVVVADNVHDVVFKSGKNVLIEFYAPWCGHCKKLAPILDEAAATLQSEEDVVIAKMDATANDVPSEFDVQGYPTLYFVTPSGKKVSYEGGGTADEIVDYIKKNKETAGQAAAADTEKAAEPAATEPLKDEL*

>AePDIL2-1

MAAMPMPRSLLLILLLATPLLILPLAAAAVPTSNPDIDLEYLIKNAGLDDPTPATTATDPEDDGAPDFPGLDADYDDEDLFGDDDGPEEDSSHPSAADEAHVLLLTAANFTSVLAARRHVMVEFYAPWCGHCRALAPHYAAAAAALAEQGVDVALAKVDATEDHDLAQAHGVQGYPTLLFFIDGVPRDYAGERTKDAIVAWISKKLGPAVQNLTTADEAEKIVTGDDVAVLAYLDHLSGAHSDELAAASRLEDTISFYQTTSPDVAKLFHIDPEAKRPSVVLLKKEEEKLTVFDGEFRASAIAEFVSANKIPLITTLTQETAPAIFDNPIKKQILLFAVAKESSKFLPIIKETAKSFKGKLLFVFVERDNEEVGEPVANYFGITGNETTVLAYTGNEDAKKFFFSGEISLDTIKEFAQDFLEDKLTPSYKSDPVPESNDEDVKVVVGKSLDQIVLDESKDVLLEVYAPWCGHCQSLEPIYNKLAKYLRGIDSLVIAKMDGTNNEHPRAKPDGFPTILFYPAGKKSFEPITFEGDRTVVEMYKFLKKHAAIPFKLKRPDSSAARTDGPGSTTEGEKSSGSNPKDEL*

>AePDIL4-1

MATPQIYRKTLLPVLLLLAAAALYPAAADGDEVLALTESTFEKEVGQDRGALVEFYAPWCGHCKKLAPEYEKLAASFKKAKSVLIAKVDCDEHKSVCSKYGVSGYPTIQWFPKGSLEPKKYEGQRTAEALTEYVNSEAATNVKIAAVPSSVVVLTEETFDSVVLDETKDVLVEFYAPWCGHCKSLAPIYEKVASVFKQDEGVVIANLDADKYTSLAEKYGVSGFPTLKFFPKGNKAGEEYESGRELDDFVKFINEKSGTSRDSKGQLTSEAGLVASLDALVKEFHGAADDKRKEILSKIEEEAAKLSGPAVKHGKIYVNVAKKILQKGSDYTKKETERLHRLLEKSISPSKADEFAIKKNILSAFSS*

>AePDIL5-1

MRPAILAAVLLLLAAAASPAAALYSAGSPVLQLNPNNFKKVLNANGVVLVEFFAPWCGHCKQLTPIWEKAAGVLKGVATVAALDADAHKELAQQYGIQGFPTIKVFLPGKPPVDYEGARDVKPIVNFALSQVKGLLRDRLDGKTSGGSSGKTSGGSREKKTEPNESVELNSSNFDELVVKSKDLWIVEFFAPWCGHCKKLAPEWKRAAKNLKGQVKLGHIDCDSDKSLMSKYKVEGFPTILVFGADKESPFPYQGARAASAIESFALEQLEANAAPPEVSELTSADVMEEKCASAAICFVSFLPDILDSMAEGRNKYLELLLSVAEKFKKSPYSFVWAGAGKQADLEKQVGVGGYGYPAMVALNVKKGAYAPLRSAFELAEITEFVKEAGRGGKGNLPLEGAPTVVQSEPWDGKDGEVIEEDEFSLEELMADSSAPNDEL*

>AePDIL6-1

MDPARRSRLPIHLLLVAVTLLAALAARSDAEVITLTEETFSDKIKEKDTVWFVQFCVPWCKHCKSPGTLWEDLGKVIEGTDEIEIGEVDCGASKPVCSKVDIHSYPTFKVFYDGEEVAKYKGPRDVESLMTFVLNVAEKAGEVRLEDEL*

>AePDIL7-1

MPAMAVDKQRLLPLFVLALVTACLVSGGEEPARFQIPRDGSVVELDEGNFEAAVAAVDFLFVDFHAPWCGHCKRLSPQLDEAAPVLAGLSTPIVVAKVNAEKYKKLRSKYGVDGFPTLMLFDHGVPTEYTGSRKAGQLVESLRKLVAPDVSVLKSDAAIKSFVQEAGVGFPLFIGFGVDESSIAEYGARYKKKAWFSTAKDFSEDLMAVYDFDKIPALVSLNPKYNEQSVFYGPFEGTFLEDFIRQSLLPMTVPINAETVKMLKDDDRKVVLAVLQDDSDETSMRLIKILRSAANANHDLVFGYVGVNQWEEFTEPFHDSKSSQLPKLVVWDKDEKYEVVEGLEKLEEGDHGSQISRFLEAYRAGRTIKKTFGRRFPTLLGVNALYILLLLVAVLVVLMFFSGQGEEDRQPTRAHQE*

>AePDIL8-1

MISSSKLKSVDFYRKIPRDLTEASLSGAGLSIFAALAMVFLFGMELSSYLAVNTTTSVIVDRSSDGEFLRIEFNLSFPALSCEFASVDVSDVLGTNRLNITKTVRKFSIDRNLVPTGSEFHAGPIPTVNKHGDDVEEYHGDGSVALSSRNFDSYSHLYPVLVVNFYAPWCYWSNRLKPSWEKAAQIIRERYDPEMDGRILLGKVDCTEEVELCKRHHIQGYPSIRIFHKGSDMKENQGHHDHDSYYGERDTESLVAAMETYVANIPKEAHVLALEDKSNKTVDPAKRPAPMTGGCRIEGFVRVKKVPGSVVISARSGSHSFDPSQINVSHYVTTFSFGKRLSSKMFNELKRLFPYVGGHHDRLAGQSYIVKHGDVNANVTIEHYLQIVKTELVTLRYSKELKVLEEYEYTAHSSLVHSFYVPVVKFHFEPSPMQVLVTELPKSFSHFITNVCAIIGGVFTVAGILDSILHNTLRLVKKVELGKDI*
