# Supplementary figures and images for: Molecular Characterization and Expression Profiling of the Protein Disulfide Isomerase Gene Family in Brachypodium distachyon L
Source: PLoS One. 2014 Apr 18;9(4):e94704. doi: 10.1371/journal.pone.0094704 (PMC3991636; doi:10.1371/journal.pone.0094704)

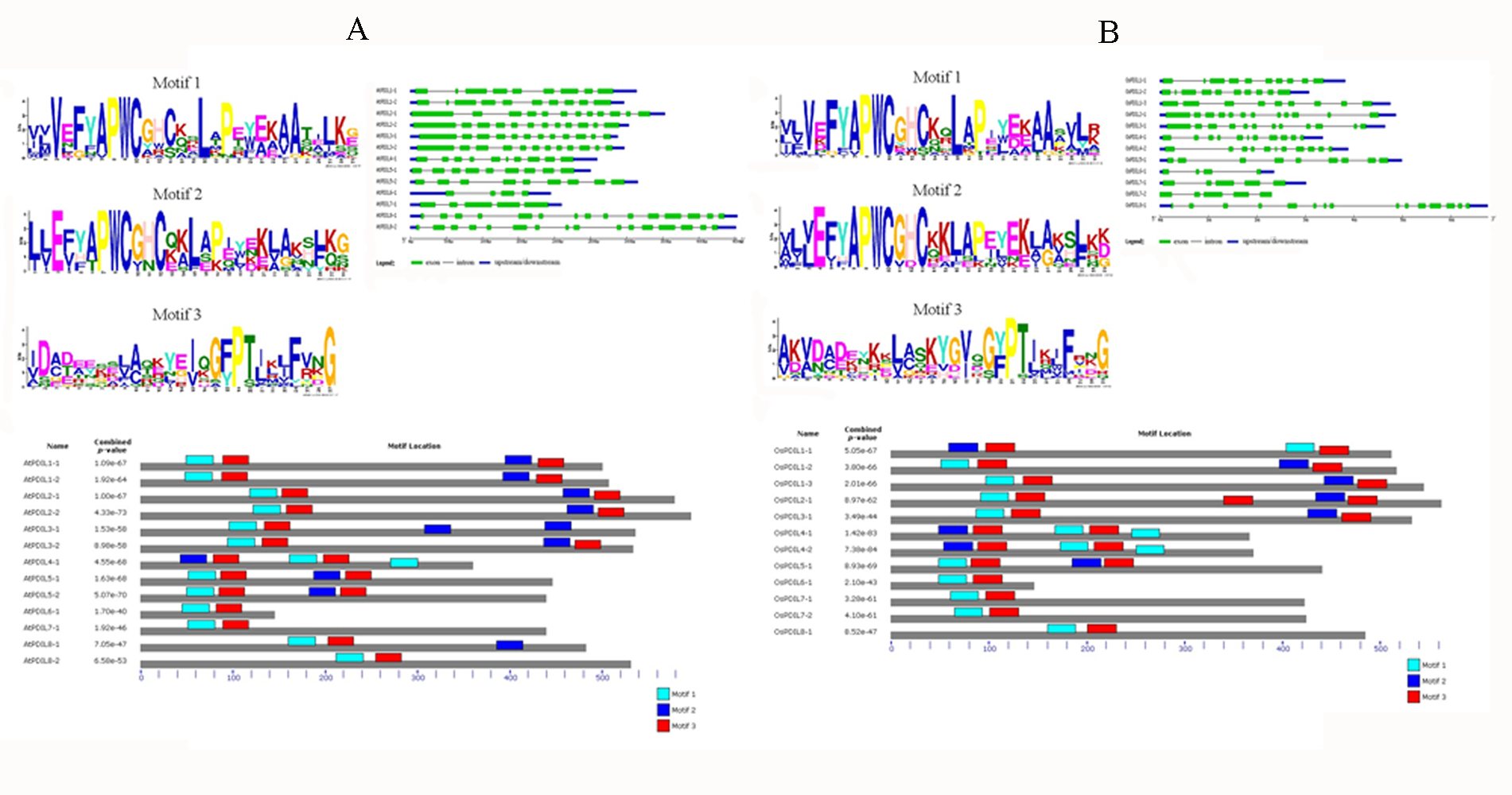

Supplement: File S2 — Motif, exon/intron structure organizations, and locations in respective sequences of PDI and PDIL family members in Arabidopsis thaliana (A) and Oryza sativa (B). (TIF) [file pone.0094704.s002.tif]

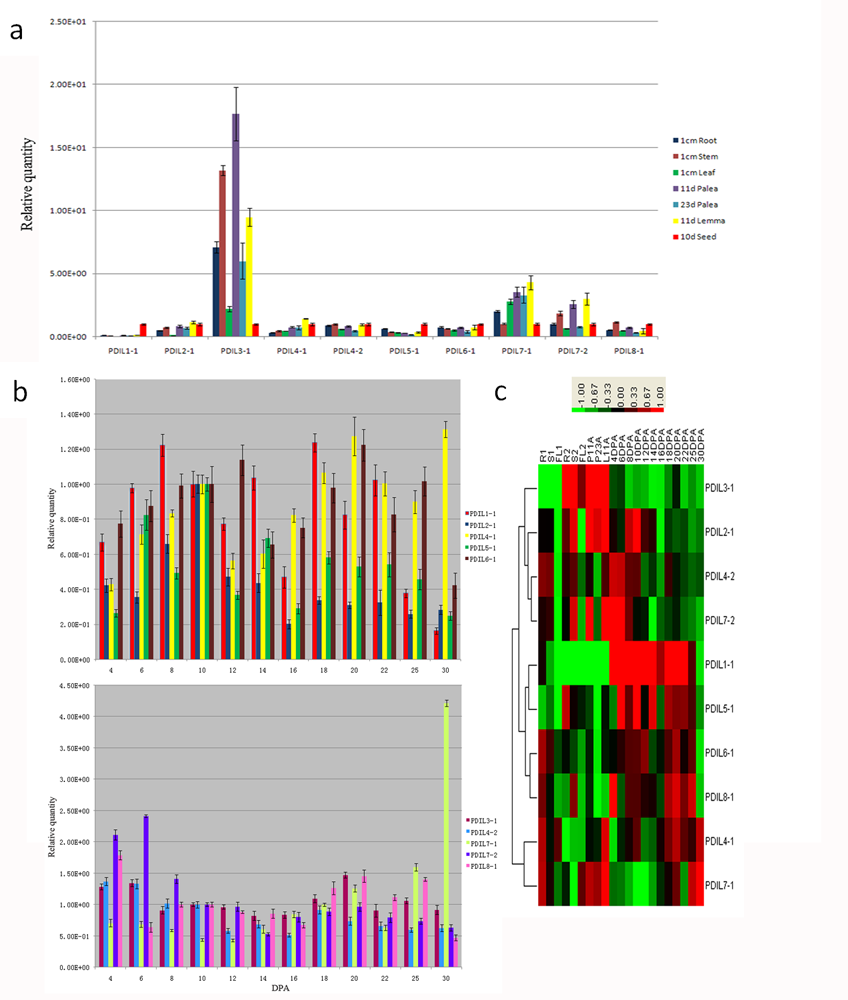

Supplement: File S4 — Organ-specific expression of Brachypodium PDI and PDIL genes in roots, stems, leaves, paleas, lemmas and developing caryopses. The relative expression levels at 10 DAP were set to value 1 as the calibrator in the pictures a and b. (TIF) [file pone.0094704.s004.tif]

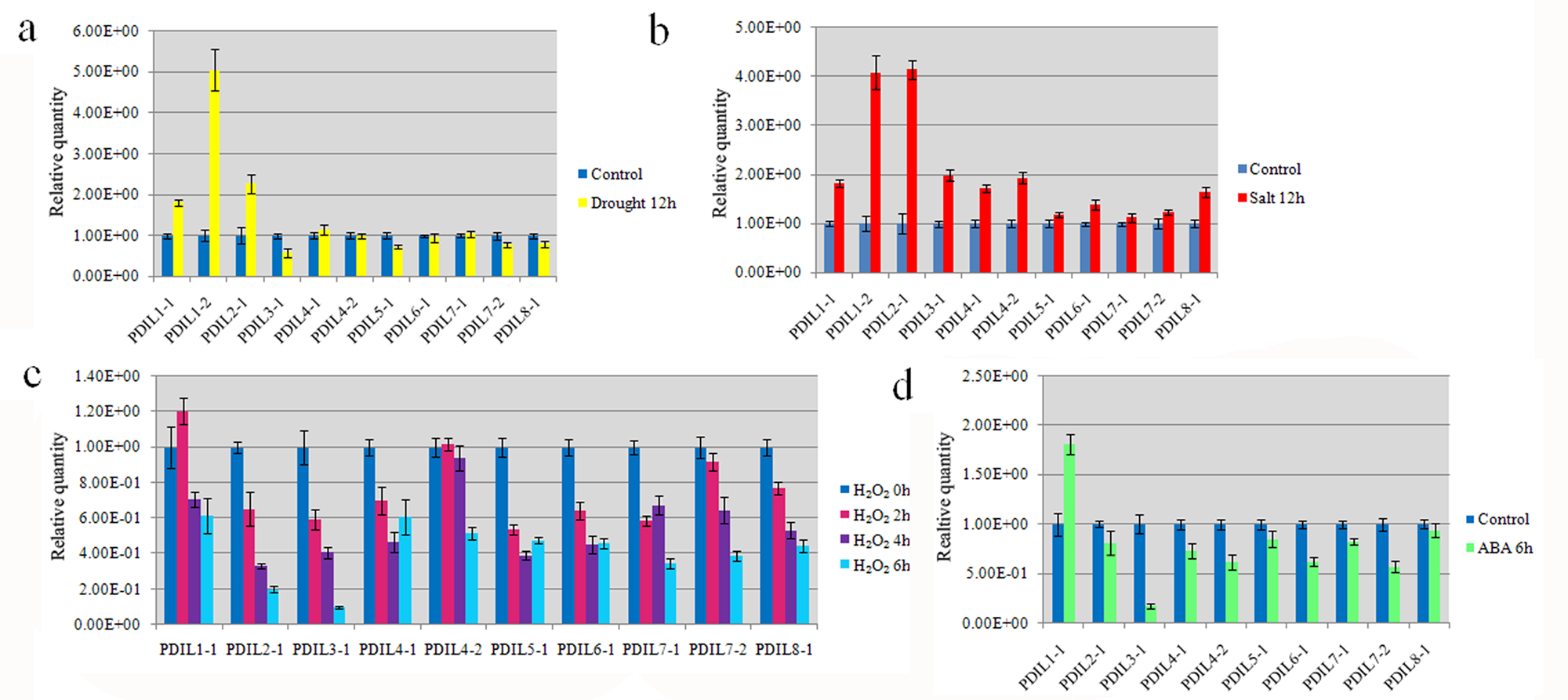

Supplement: File S5 — Relative expression levels of BdPDI family members in the leaves of B. distachyon in response to drought (a), salt (b), H2O2 (c) and ABA (d) stress were analyzed using quantitative RT-PCR and compared with well-watered control plants. The right side of illustrations indicated the treatment time (hour) under corresponding abiotic stresses. Error bars represented the standard deviations of three biological replicates. (TIF) [file pone.0094704.s005.tif]

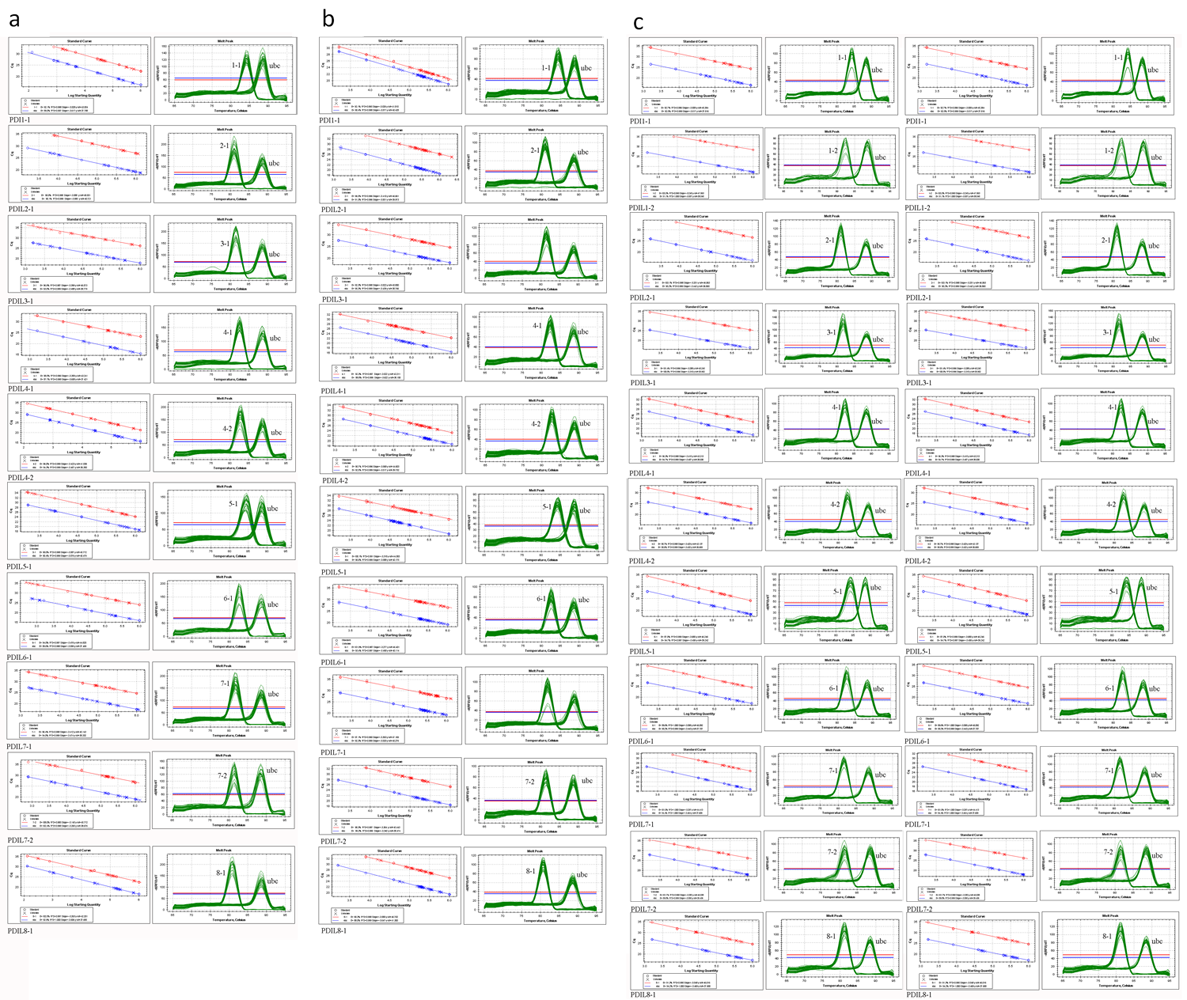

Supplement: File S7 — qRT-PCR optimization design: double standard curve and dissolution curve of BdPDI and BdPDIL genes in different developing organs (a) developing caryopses (b) and leaves under drought, salt, H2O2 and ABA treatments (c). One of the red standard curves represented BdPDI genes and other blue standard curve represented the reference gene. The dissolution curves of different genes were indicated. (TIF) [file pone.0094704.s007.tif]
