# Supplementary material for: Molecular Characterization and Expression Profiling of the Protein Disulfide Isomerase Gene Family in Brachypodium distachyon L
Source: PLoS One. 2014 Apr 18;9(4):e94704. doi: 10.1371/journal.pone.0094704 (PMC3991636; doi:10.1371/journal.pone.0094704)
Supplement: File S6 — Primer sequences used for real-time quantitative RT-PCR (qRT-PCR). (DOCX) [file pone.0094704.s006.docx]

**File S6. Primer sequences used for real-time quantitative RT-PCR (qRT-PCR)**

| Gene | Primer name | Primer sequence | PCR product size |
| --- | --- | --- | --- |
| *BdPDIL 1-1* | BdPDIL 1-1F | CAGGGGAAGAACATTCAGGA | 164bp |
|  | BdPDIL 1-1R | AATTCGGCAAAGACACCAAC |  |
| *BdPDIL 1-2* | BdPDIL 1-2F | GATGCGCTCGGATTATGATT | 210bp |
|  | BdPDIL 1-2R | TTGGTCGGTTCATCATCAAA |  |
| *BdPDIL 2-1* | BdPDIL 2-1F | TTGTGGAGCGAGACAATGAG | 214bp |
|  | BdPDIL 2-1R | TCGGGTATTGGCTCAGACTT |  |
| *BdPDIL 3-1* | BdPDIL 3-1F | GCCAAGCATTTCAGTGGTTT | 233bp |
|  | BdPDIL 3-1R | GAAGCTGGCTCCTTCTCCTT |  |
| *BdPDIL 4-1* | BdPDIL 4-1F | TGCCCCTGAATATGAAAAGC | 181bp |
|  | BdPDIL 4-1R | AGTGCGCTGTCCCTCATACT |  |
| *BdPDIL 4-2* | BdPDIL 4-2F | TAGCGGCAGTTCCTTCAAGT | 160bp |
|  | BdPDIL 4-2R | TTGAAAGCAGAAGCCACCTT |  |
| *BdPDIL 5-1* | BdPDIL 5-1F | CTGCTGCCATTTGCTTTGTA | 218bp |
|  | BdPDIL 5-1R | CTTCACGTTCAGAGCAACCA |  |
| *BdPDIL 6-1* | BdPDIL 6-1F | ACCCTCACCGAAGAGACCTT | 189bp |
|  | BdPDIL 6-1R | TACTGGTTTGCTTGCACCAC |  |
| *BdPDIL 7-1* | BdPDIL 7-1F | GGATCGAAATACGGAGTGGA | 184bp |
|  | BdPDIL 7-1R | TGCCAGCATTCTCAACAAAG |  |
| *BdPDIL 7-2* | BdPDIL 7-2F | TTTATTGGATTTGGGGTGGA | 191bp |
|  | BdPDIL 7-2R | TCGAATGGGCCATAGAAAAC |  |
| *BdPDIL 8-1* | BdPDIL 8-1F | CCCAGTTCTCATTTGGCAAT | 242bp |
|  | BdPDIL 8-1R | GCTGTGCGCTGTGTATTCAT |  |
| *Ubi4* | Ubi4F | TGACACCATCGACAACGTGA | 142bp |
|  | Ubi4R | GAGGGTGGACTCCTTCTGGA |  |
